# Revealing the association between East Asian oral microbiome and colorectal cancer through Mendelian randomization and multi-omics analysis

**DOI:** 10.3389/fcimb.2024.1452392

**Published:** 2024-09-17

**Authors:** Yuheng Gu, Lai Jiang, Min Shui, Honghao Luo, Xuancheng Zhou, Shengke Zhang, Chenglu Jiang, Jinbang Huang, Haiqing Chen, Jingyi Tang, Yiping Fu, Huiyan Luo, Guanhu Yang, Ke Xu, Hao Chi, Jie Liu, Shangke Huang

**Affiliations:** ^1^ School of Clinical Medicine, The Affiliated Hospital, Southwest Medical University, Luzhou, China; ^2^ Department of Oncology, Chongqing General Hospital, Chongqing University, Chongqing, China; ^3^ Department of Radiology, Xichong People’s Hospital, Nanchong, China; ^4^ Department of Specialty Medicine, Ohio University, Athens, OH, United States; ^5^ Department of general surgery, Dazhou Central Hospital, Dazhou, China; ^6^ Department of Oncology, The Affiliated Hospital, Southwest Medical University, Luzhou, China

**Keywords:** oral microbiome, colorectal cancer, Mendelian randomization, single-cell RNA sequencing, therapeutic targets

## Abstract

**Background:**

Colorectal cancer (CRC) poses a global health threat, with the oral microbiome increasingly implicated in its pathogenesis. This study leverages Mendelian Randomization (MR) to explore causal links between oral microbiota and CRC using data from the China National GeneBank and Biobank Japan. By integrating multi-omics approaches, we aim to uncover mechanisms by which the microbiome influences cellular metabolism and cancer development.

**Methods:**

We analyzed microbiome profiles from 2017 tongue and 1915 saliva samples, and GWAS data for 6692 CRC cases and 27178 controls. Significant bacterial taxa were identified via MR analysis. Single-cell RNA sequencing and enrichment analyses elucidated underlying pathways, and drug predictions identified potential therapeutics.

**Results:**

MR identified 19 bacterial taxa significantly associated with CRC. Protective effects were observed in taxa like RUG343 and Streptococcus_umgs_2425, while HOT-345_umgs_976 and W5053_sp000467935_mgs_712 increased CRC risk. Single-cell RNA sequencing revealed key pathways, including JAK-STAT signaling and tyrosine metabolism. Drug prediction highlighted potential therapeutics like Menadione Sodium Bisulfite and Raloxifene.

**Conclusion:**

This study establishes the critical role of the oral microbiome in colorectal cancer development, identifying specific microbial taxa linked to CRC risk. Single-cell RNA sequencing and drug prediction analyses further elucidate key pathways and potential therapeutics, providing novel insights and personalized treatment strategies for CRC.

## 1 Introduction

Colorectal cancer (CRC) remains one of the most prevalent cancer types globally. According to the 2020 GLOBOCAN statistics, CRC is ranked as the third most commonly diagnosed cancer worldwide, accounting for 10% of cases, and the second leading cause of cancer death, responsible for 9.4% of mortality (Sung et al., 2021). Furthermore, projections suggest a significant increase of approximately 3.2 million new CRC cases by 2040, posing substantial challenges to global healthcare systems (Xi and Xu, 2021). The incidence of CRC is higher in highly developed nations and is on the rise in middle- to low-income countries due to Westernization (Kwong et al., 2018).

Over the past decade, dysbiosis in the oral microbiome has enhanced our understanding of the pathogenesis of oral cancers and other diseases in distant organs. Porphyromonas gingivalis, a primary pathogen in periodontal disease (Mysak et al., 2014), is also implicated in other cancers such as pancreatic cancer (Öğrendik, 2017). Additionally, Candida albicans infections can activate oncogenes, overexpress inflammatory signaling pathways, and induce DNA damage, contributing to the progression of oral cancer and the onset of gastric cancer (Zaura et al., 2014; Engku Nasrullah Satiman et al., 2020). Studies have also indicated a link between the oral microbiome and colorectal cancer (Collins et al., 2011; Warren et al., 2013; Davey Smith and Hemani, 2014).

Based on these literary evidences, the oral microbiome may exert a distal influence on the onset and progression of colorectal cancer. We will employ Mendelian Randomization (MR), a powerful tool for causal inference in epidemiology (Bowden and Holmes, 2019; Weith and Beyer, 2023). Unlike conventional observational studies, MR as a genetic variation-based method for causal inference, effectively addresses the limitations of observational studies. Traditional observational studies often struggle with confounding factors and reverse causation, making accurate causal inference challenging. MR uses genetic variants, such as single nucleotide polymorphisms (SNPs), as instrumental variables. These variants are randomly allocated according to Mendel’s laws, ensuring that they are independent of confounding factors. This approach minimizes bias inherent in traditional observational studies, enabling more accurate identification of causal relationships between exposures and diseases, thereby enhancing the reliability and validity of research finding (Davey Smith and Hemani, 2014; Li et al., 2024). MR leverages genetic variants as instrumental variables to assess the causal relationship between the oral microbiome and colorectal cancer. This approach will aid in determining the true role of the oral microbiome in the development and progression of colorectal cancer. Moreover, this study will integrate single-cell transcriptomics and bulk RNA sequencing technologies to elucidate the underlying mechanisms of the oral microbiome in CRC development comprehensively (Yazar et al., 2022). Single-cell transcriptomics offers high-resolution insights into cell types and functional characteristics, facilitating a better understanding of the interactions between the oral microbiome and colorectal cancer (Jiang et al., 2024). In contrast, bulk RNA sequencing provides an overview of gene expression, further validating and complementing the findings from single-cell transcriptomics. Additionally, our research will analyze and align potential therapeutic drugs to explore new treatment strategies for colorectal cancer. By combining the regulatory mechanisms of the oral microbiome with existing drug databases, we can identify potential therapeutic agents and further validate their efficacy and safety. Our aim is to more accurately assess the relationship between the oral microbiome and colorectal cancer, uncover the mechanisms involved, and provide new insights and strategies for personalized treatment of colorectal cancer.

## 2 Materials and methods

### 2.1 MR design

The interplay between the oral microbiome and colorectal cancer is an area of growing scientific inquiry. There is increasing evidence that suggests oral bacteria may influence the development of colorectal cancer through mechanisms such as microbial dysbiosis and systemic inflammation.

This study adheres to the STROBE-MR guidelines, which are part of the broader Strengthening the Reporting of Observational Studies in Epidemiology (STROBE) initiative, to ensure high-quality reporting of observational data.

To ensure the integrity of the Mendelian Randomization analysis, the genetic variants serving as instrumental variables must satisfy three critical assumptions: (1) the variants must show strong associations with specific taxa of the oral microbiome, thereby clearly defining the exposure variable in our study; (2) the variants must be independent of unmeasured confounders that could potentially bias the results, ensuring that observed associations are not affected by factors such as lifestyle or genetic background; (3) the impact of these variants on the risk of colorectal cancer must occur exclusively through alterations in the oral microbiome, thus excluding any alternative mediating pathways and emphasizing the unique role of the microbiome (Emdin et al., 2017).

### 2.2 Data source

The exposure data for this study were sourced from CNGBdb and encompassed comprehensive microbiome profiles from an East Asian cohort. This dataset included 309 tongue dorsum microbiomes with a total of 2,017 samples and 285 salivary microbiomes comprising 1,915 samples (Liu et al., 2021). Notably, these samples represent the first large-scale collection of its kind, featuring high-depth whole genome sequencing. Rigorous criteria were employed to ensure data quality, including a variant calling rate of no less than 98%, a mean sequencing depth exceeding 20x, and the absence of population stratification as confirmed by principle component analysis (PCA). Additionally, related individuals were excluded based on pairwise identity by descent estimates. The study further implemented strict selection protocols, requiring a minimum mean depth of 8x, Hardy-Weinberg equilibrium (HWE) values greater than 10^-5, and a genotype calling rate above 98%. Following these stringent quality control measures, a robust cohort of 2,984 participants was established, comprised of 2,017 individuals with tongue dorsum samples and 1,915 with salivary samples. The dataset maintained for analysis included approximately 10 million genetic variants, both common and low-frequency, with a minor allele frequency (MAF) of at least 0.5%.

In contrast, the genome-wide association study (GWAS) data for colorectal cancer were obtained from the Biobank Japan (BBJ), which included a sample size of 33,870, encompassing 6,692 patients and 27,178 controls from the general population. This comprehensive data collection facilitated the exploration of genetic correlations and potential causative links between the oral microbiome and colorectal cancer within this population. Detailed information could be viewed in **Table 1**.

**Table 1:** This table summarizes the dataset characteristics for the oral microbiome and colorectal cancer studies.

Exposures/Outcomes	Consortium	Ethnicity	Sample sizes	N. SNPs	Year
Oral microbiome	CNGBdb	East Asian	2948	Tongue N = 8426	2021
			Tongue N = 2017	Saliva N = 8009	
			Saliva N= 1914		
Colorectal cancer	BioBank Japan	East Asian	33870	7492477	2019

For the oral microbiome, data were sourced from the China National GeneBank (CNGBdb) and included 2,948 samples from East Asian individuals, further divided into 2,017 tongue and 1,914 saliva samples, with respective SNP counts. The colorectal cancer data, obtained from Biobank Japan (BBJ), included 33,870 East Asian participants, comprising 6,692 patients and 27,178 controls, with a total of 7,492,477 SNPs collected in 2019.

### 2.3 Genetic instruments selection

Prior to data analysis, several criteria were established to optimize the selection of instrumental variables. Firstly, a significance threshold was set with p-values greater than 1x10^-5, allowing a liberal inclusion of SNPs to enhance statistical power. Linkage disequilibrium (LD) was calculated using reference populations such as the 1000 Genomes European panel, selecting SNPs with a low LD threshold (r^2 < 0.001, 10,000 kb) and prioritizing those with lower p-values. Only SNPs with an effect allele frequency (EAF) greater than 0.01 were retained, ensuring the variants’ prevalence. Specific SNPs, including palindromic SNPs and those with an F-statistic below 10, were excluded to avoid weak instrumental variables and reduce bias, where the F-statistic is calculated using the formula: F = (beta/se)^2 (Lv et al., 2023). Finally, Steiger filtering was conducted to retain SNPs where the exposure’s R-squared was greater than that of the outcome, ensuring the instrumental variables did not exhibit reverse causality (Hemani et al., 2017).

### 2.4 MR analysis

In our study, the inverse variance-weighted (IVW) method (Burgess and Thompson, 2015) served as the primary analytical technique for assessing the causal impacts of oral microbiome taxa on colorectal cancer. This approach aggregates the effects associated with all SNPs to produce a comprehensive estimate. To further explore the robustness and validity of the instrumental variables, additional Mendelian Randomization methods such as MR Egger (Bowden et al., 2015), weighted median (Bowden et al., 2016), and weighted mode (Hartwig et al., 2017) were implemented. To address the possibility of reverse causation, positive MR findings were subjected to the Steiger directionality test.

We applied multiple testing correction using the false discovery rate (FDR) method to adjust p-values. This was performed separately for saliva and tongue microbiome data, and grouped by Phylum, Class, and Order to enhance the detection of significant associations while maintaining rigorous statistical standards. This stratified approach allows for more refined detection of significant associations, balancing rigorous statistical control with the exploratory nature of our study to identify biologically relevant signals.

To address the possibility of reverse causation, positive MR findings were subjected to the Steiger directionality test.

To evaluate the presence of horizontal pleiotropy, our analysis incorporated the MR-PRESSO and MR-Egger regression tests. Each SNP underwent the MR-PRESSO (Verbanck et al., 2018) outlier test to ascertain its significance concerning pleiotropic effects, with each test generating a distinct p-value. The overarching pleiotropy was then assessed using the MR-PRESSO global test, which recalibrated the global test p-value following the sequential removal of SNPs, starting with those displaying the lowest outlier test p-values. This iterative removal continued until the global test p-value surpassed the threshold of 0.05, suggesting an absence of significant pleiotropic influences. The resulting set of SNPs, cleared of pleiotropic biases, was utilized in the further stages of the MR analysis. The Cochran Q analysis (Shen et al., 2022) is used to assess heterogeneity among the instrumental variables (IVs) in a Mendelian Randomization (MR) study. If the p-value from the Cochran Q test is above 0.05, indicating no evidence of significant heterogeneity, a fixed-effects inverse variance-weighted (IVW) method is employed as the primary analytical approach. Conversely, if significant heterogeneity is detected (p < 0.05), a random-effects IVW method is utilized to accommodate the variability among the IVs. is used to assess heterogeneity among the instrumental variables (IVs) in a Mendelian Randomization (MR) study. If the p-value from the Cochran Q test is above 0.05, indicating no evidence of significant heterogeneity, a fixed-effects inverse variance-weighted (IVW) method is employed as the primary analytical approach. Conversely, if significant heterogeneity is detected (p < 0.05), a random-effects IVW method is utilized to accommodate the variability among the IVs.

In addressing the potential issue of reverse causation within our Mendelian Randomization study, we subjected all positive findings to rigorous scrutiny using the Steiger directionality test. This methodological step ensures that the observed associations are not a result of the outcome influencing the exposure, thereby reinforcing the credibility of our causal inferences.

All statistical computations were conducted using R software version 4.1.3 (R Foundation for Statistical Computing, Vienna, Austria). The analyses employed the “TwoSampleMR” package version 0.5.8, designed specifically for MR investigations.

### 2.5 SNP annotation

For the annotation of SNPs, we utilized the VarNote database (Huang et al., 2020), which is distinguished by its innovative indexing system and a parallel random-sweep searching algorithm. This system enables VarNote to deliver substantial enhancements in performance, accelerating processing by two to three orders of magnitude compared to existing solutions. VarNote supports both region-based and allele-specific annotations and offers advanced functionalities for the flexible retrieval of detailed annotations, making it well-suited for complex genomic analyses.

In our study, the parameters set for SNP annotation through VarNote were tailored to optimize the relevance and precision of the data. The annotations were specifically geared towards tissue/cell type-specific epigenomes, with a focus on E127 (NHEK-Epidermal Keratinocyte Primary Cells: CellLine). For the prioritization of variants, prediction scores such as FitCons2, FUNLDA, GenoNet, and GenoSkylinePlus were utilized. The population reference was set to European (EUR), ensuring the relevance of the data to the demographic of interest. Additionally, a linkage disequilibrium (LD) cutoff of 0.8 and an LD window of 100KB were applied, with gene annotations referencing the Ensembl database.

### 2.6 Gene function enrichment

To analyze the biological pathways and processes significantly associated with our identified genes, we conducted an enrichment analysis using the clusterProfiler R package (version 4.4.4) (Wu et al., 2021). First, gene identifiers were accurately mapped to human genes (Homo sapiens) using the org.Hs.eg.db package. This step ensured the precision of our molecular data before proceeding with the enrichment analysis. The clusterProfiler facilitated a detailed exploration into the biological pathways that were significantly enriched, focusing on those related to fatty acid metabolism and their influence on blood glucose levels.

For a clear and intuitive presentation of these results, we utilized the sangerbox tool to create enrichment circle plots, as detailed by Shen et al. (2022) (Shen et al., 2022). These plots provided a visual summary of the key pathways enriched in our study, offering a user-friendly way to interpret the complex interactions and implications of the identified genes. This method of visualization helped emphasize the most pertinent biological processes and pathways involved in our analysis.

### 2.7 Clustering and annotation of single-cell RNA sequencing data

Single-cell analysis employed nine cancer samples from dataset GSE205506 and four normal control samples from GSE231993, all of which were downloaded from the Gene Expression Omnibus (GEO) database (www.ncbi.nlm.nih.gov/geo).

To process a dataset of single-cell RNA sequencing from colorectal cancer, the Read10X function is utilized to import the data into an R environment, where it is subsequently converted into a Seurat object using the “Seurat” package(version 4.3.0.1). Quality control metrics are calculated by assessing the proportion of mitochondrial and ribosomal genes, as well as erythrocyte content within each cell. Cells with gene counts below 4000 or above 200, and those with a mitochondrial gene proportion exceeding the allowable threshold of 20% (pctMT = 20), are excluded to remove low-quality cells from the dataset. Subsequently, the NormalizeData function is applied to standardize the merged dataset. For the analysis of single-cell RNA sequencing (scRNA-seq) data, Principal Component Analysis (PCA) is employed for dimensionality reduction and clustering. Initially, the FindVariableFeatures function identifies the top 2000 highly variable genes. Principal component scores from 1 to 8 are assigned for the dimensionality reduction step. Finally, t-distributed Stochastic Neighbor Embedding (t-SNE) is generated to visualize the resulting unsupervised cell clusters. To annotate the cell type of each cluster, marker genes from previous studies are used. We employed the `ggplot2` package(version 3.4.3)in R for heatmap generation, a robust tool for data visualization. The `ggplot2` package, based on The Grammar of Graphics, allows for complex, layered visualizations.

### 2.8 Drug accessibility analysis using DSigDB

The Drug Signatures Database (DSigDB) is an extensive repository (Freshour et al., 2021) that contains 22,527 gene sets and 17,389 different compounds, covering 19,531 genes. It plays a crucial role in bridging drugs and other chemical entities with their target genes. By inputting genes relevant to specific diseases or those that show significant expression changes under certain biological conditions, DSigDB facilitates the prediction of potential small molecule drugs. This prediction is based on the connections between the inputted genes and known drug target genes as well as drug sensitivity genes, providing a valuable tool for understanding drug-gene interactions and enhancing drug discovery processes.

### 2.9 Molecular docking

Obtain the three-dimensional crystal structure of the target protein in PDB format through the RCSB PDB database (https://www.rcsb.org/) and the two-dimensional structure of the active component in SDF format through the Pubchem database (https://pubchem.ncbi.nlm.nih.gov/), saving the small molecule in mol2 format. Use PyMOL software to preprocess the target protein by removing solvents and ligands, etc. Use AutoDockTools-1.5.7 software to preprocess the target protein by removing water, adding hydrogens, and calculating charges, and preprocess the active component by adding hydrogens and setting torsion angles, etc., before performing molecular docking and calculating the binding energy (affinity). Visualization software: Rymol 2.6.0.

## 3 Results

### 3.1 Research workflow

**Figure 1:**
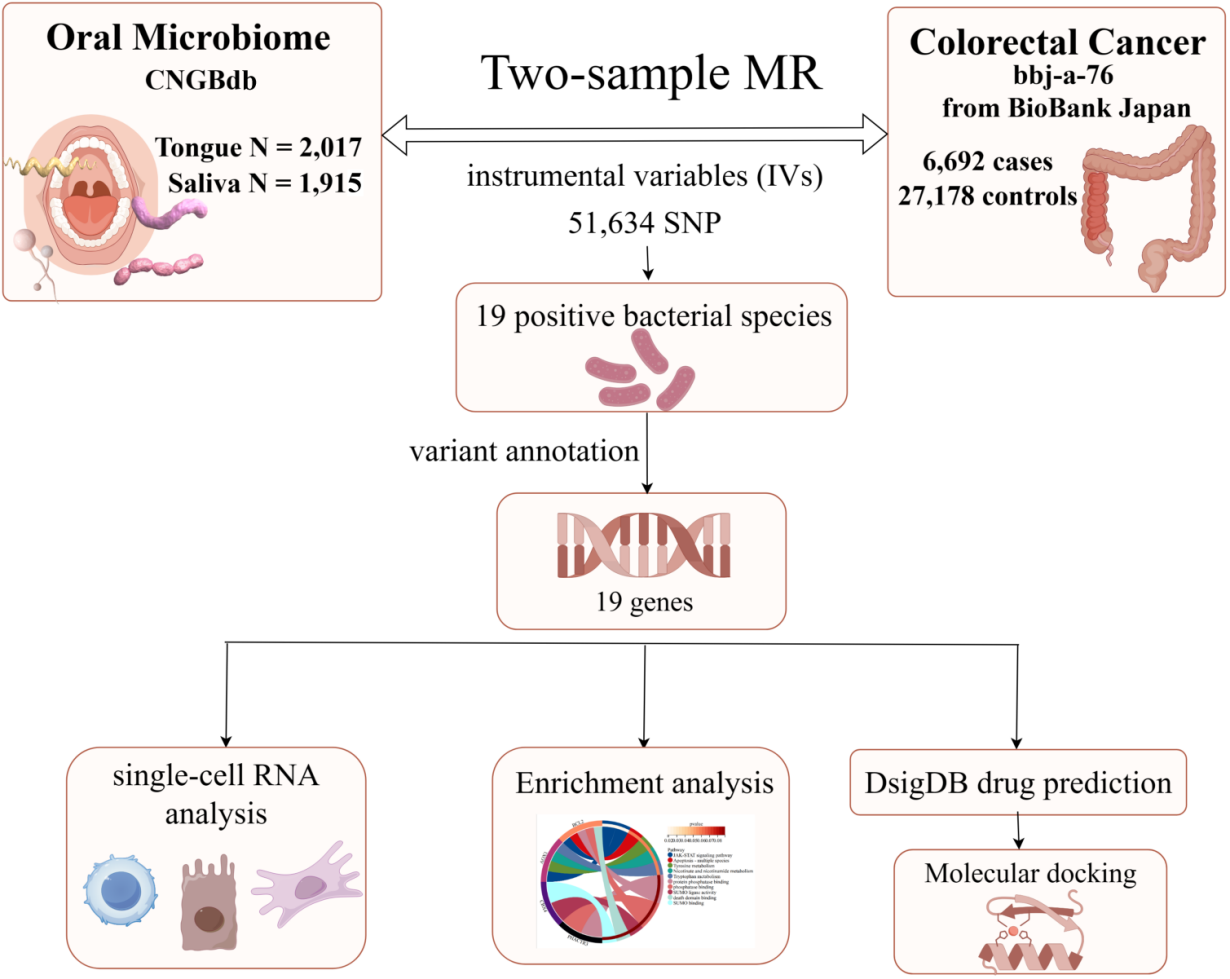
The flowchart of Mendelian randomization analysis. IVW, inverse variance weighted; MR, Mendelian randomization; MVMR, multivariable Mendelian randomization; SNPs, single nucleotide polymorphisms; IVs, instrumental variables; CNGBdb, China National GeneBank DataBase; BBJ, BioBank Japan Project.

Firstly, **Figure 1** (By Figdraw) illustrates the steps of our study, which commenced with data acquisition from the China National GeneBank (CNGBdb). The data comprises oral microbiomes from tongue samples (2,017 instances) and saliva samples (1,915 instances). Utilizing this data, we conducted two-sample Mendelian Randomization (MR) analyses, identifying instrumental variables (IVs) for colorectal cancer through 58,904 SNPs. This analysis confirmed 19 bacterial species positively associated with colorectal cancer and further annotated 19 genes related to these bacterial species. Subsequently, single-cell RNA analysis and enrichment analyses were employed to investigate the roles and interactions of these genes in the disease context. In the final phase, potential therapeutics associated with these genes were predicted using the Drug Signatures Database (DsigDB), and their interactions with target proteins were examined through molecular docking techniques, aiming to discover new methods for treating colorectal cancer. Additionally, our study incorporates data on 6,692 colorectal cancer cases and 27,178 controls from the BioBank Japan, enhancing the universality and accuracy of our findings.

### 3.2 Causal impact of the oral microbiome on colorectal cancer development

**Figure 2:**
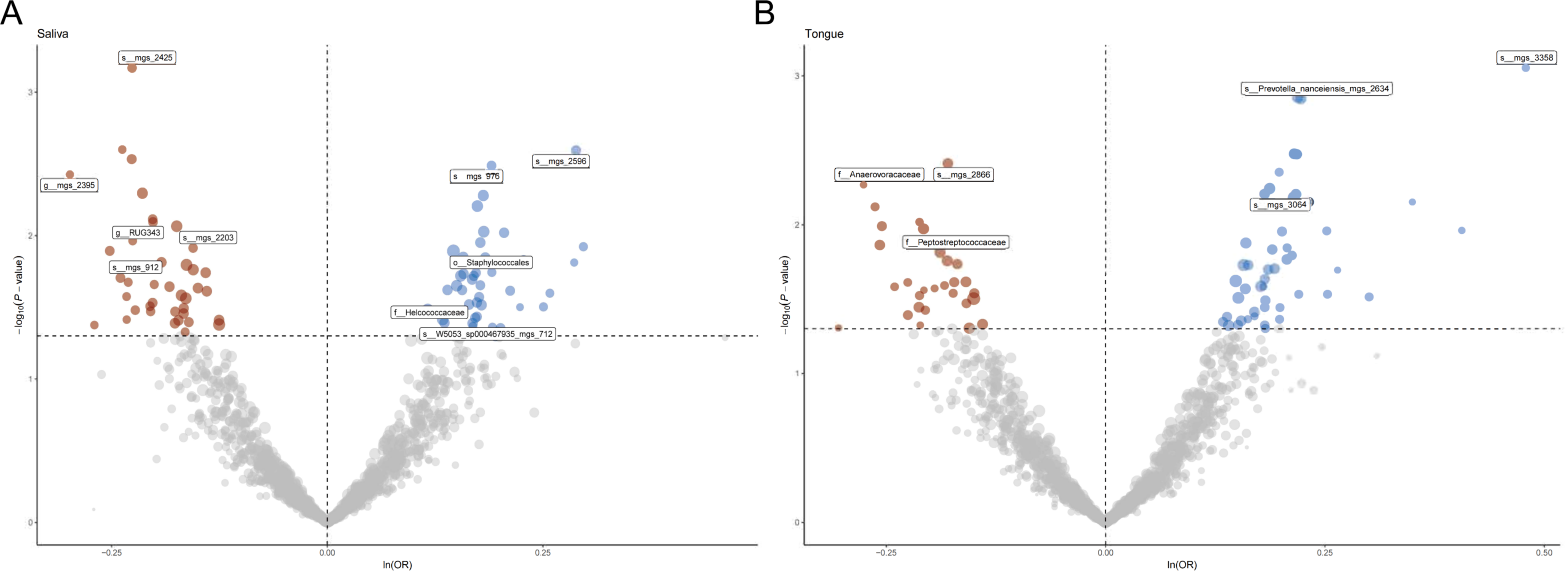
**(A)** Volcano plot illustrating the effect of single nucleotide polymorphisms (SNPs) on various taxa in saliva samples. Points are color-coded to indicate statistical significance: significant positive associations are shown in blue, significant negative associations in red, and non-significant associations in gray. Labeled taxa represent those with the most significant associations. **(B)** Volcano plot illustrating the effect of single nucleotide polymorphisms (SNPs) on various taxa in tongue a samples. Points are color-coded to indicate statistical significance: significant positive associations are shown in blue, significant negative associations in red, and non-significant associations in gray. Labeled taxa represent those with the most significant associations.

**Figure 3:**
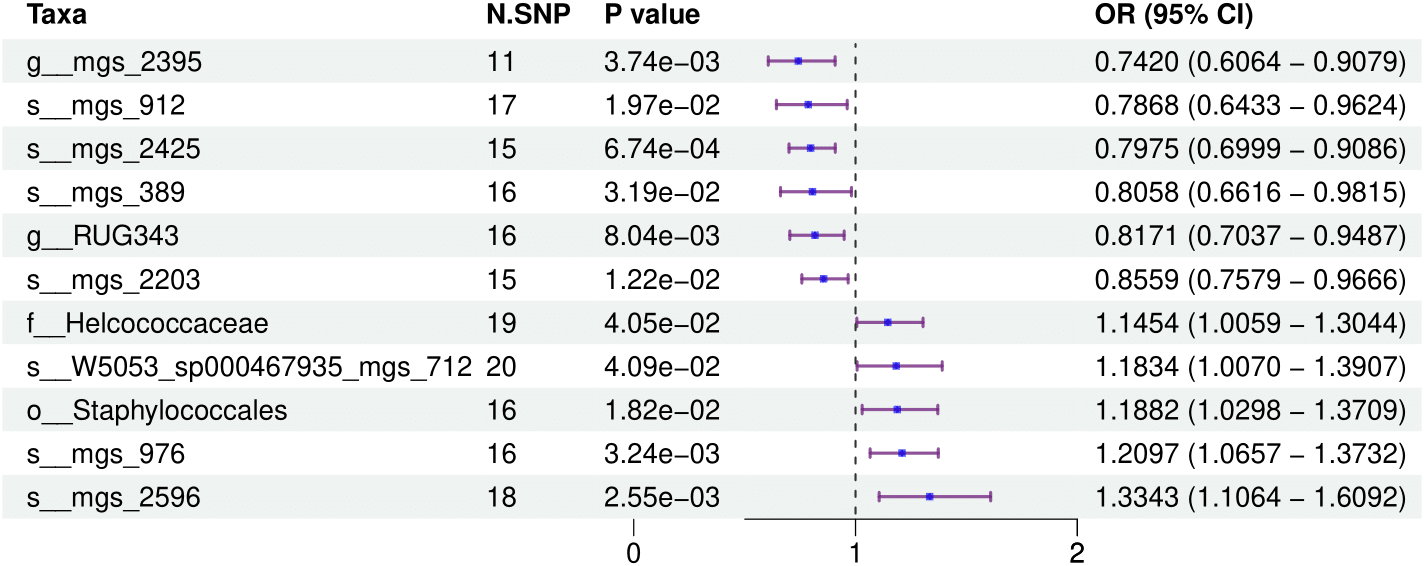
This forest plot illustrates the odds ratios (ORs) and 95% confidence intervals (CIs) for the impact of single nucleotide polymorphisms (SNPs) on various taxa within saliva samples, highlighting the statistically significant associations that affect health outcomes.

**Figure 4:**
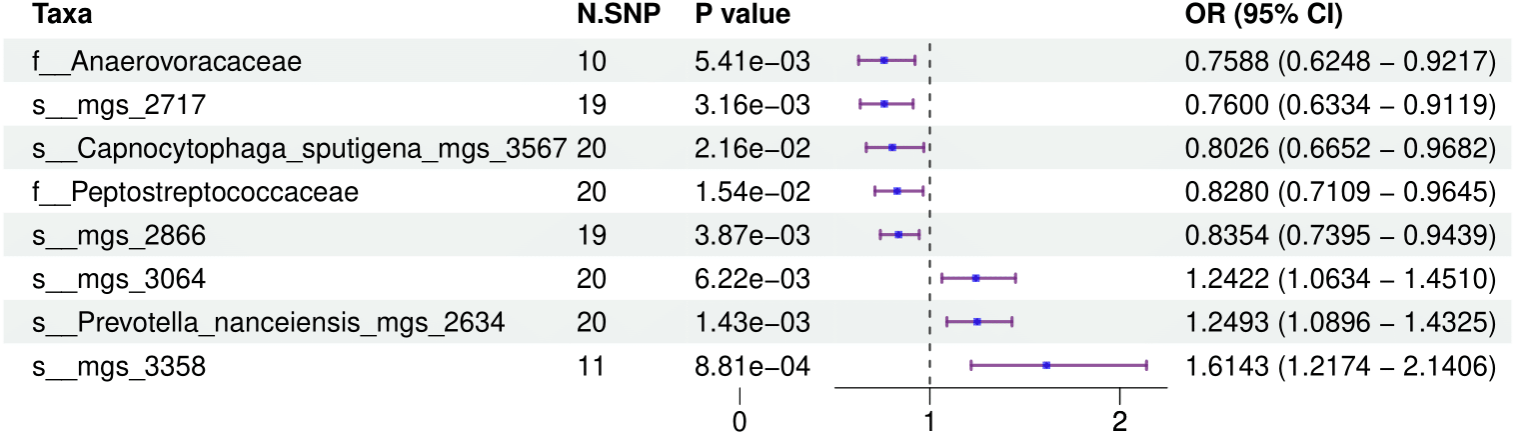
This forest plot displays the effects of SNPs on taxa within tongue samples, showing detailed ORs and 95% CIs. It identifies both positive and negative associations that these genetic variations have with health outcomes.

**Figure 5:**
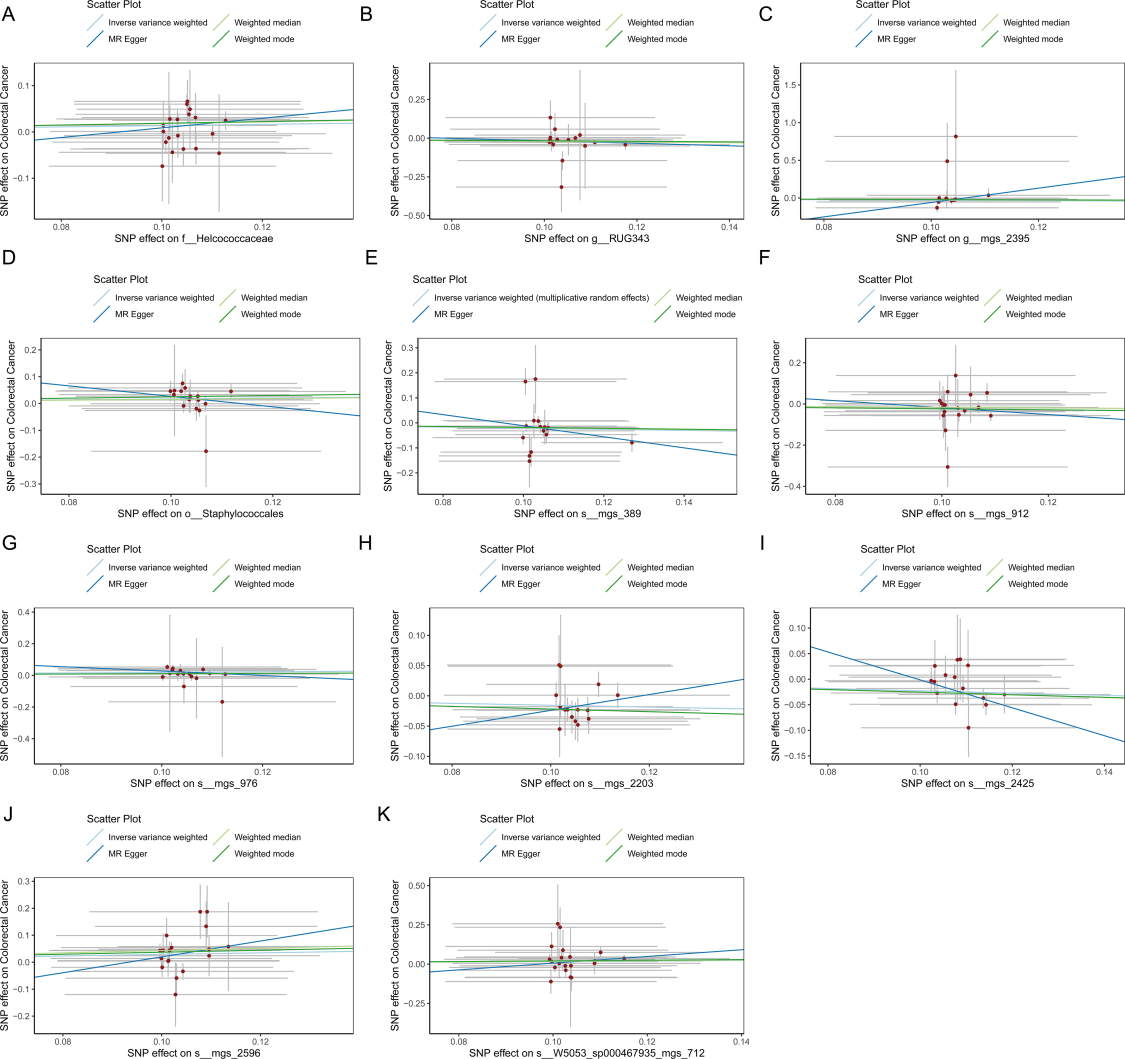
**(A)** Scatter plot depicting the relationship between single nucleotide polymorphisms (SNPs) effect on the family Helcococcaceae and their corresponding effect on colorectal cancer. **(B)** Scatter plot depicting the relationship between single nucleotide polymorphisms (SNPs) effect on the genus RUG343 and their corresponding effect on colorectal cancer. **(C)** Scatter plot depicting the relationship between single nucleotide polymorphisms (SNPs) effect on the genus g__Lachnospiraceae and their corresponding effect on colorectal cancer. **(D)** Scatter plot depicting the relationship between single nucleotide polymorphisms (SNPs) effect on the order Staphylococcales and their corresponding effect on colorectal cancer. **(E)** Scatter plot depicting the relationship between single nucleotide polymorphisms (SNPs) effect on the species mgs_389 and their corresponding effect on colorectal cancer. **(F)** Scatter plot depicting the relationship between single nucleotide polymorphisms (SNPs) effect on the species mgs_912 and their corresponding effect on colorectal cancer. **(G)** Scatter plot depicting the relationship between single nucleotide polymorphisms (SNPs) effect on the species mgs_976 and their corresponding effect on colorectal cancer. **(H)** Scatter plot depicting the relationship between single nucleotide polymorphisms (SNPs) effect on the species mgs_2203 and their corresponding effect on colorectal cancer. **(I)** Scatter plot depicting the relationship between single nucleotide polymorphisms (SNPs) effect on the species mgs_2425 and their corresponding effect on colorectal cancer. **(J)** Scatter plot depicting the relationship between single nucleotide polymorphisms (SNPs) effect on the species mgs_2596 and their corresponding effect on colorectal cancer. **(K)** Scatter plot depicting the relationship between single nucleotide polymorphisms (SNPs) effect on the species W5053_sp000467935_mgs_712 and their corresponding effect on colorectal cancer.

**Figure 6:**
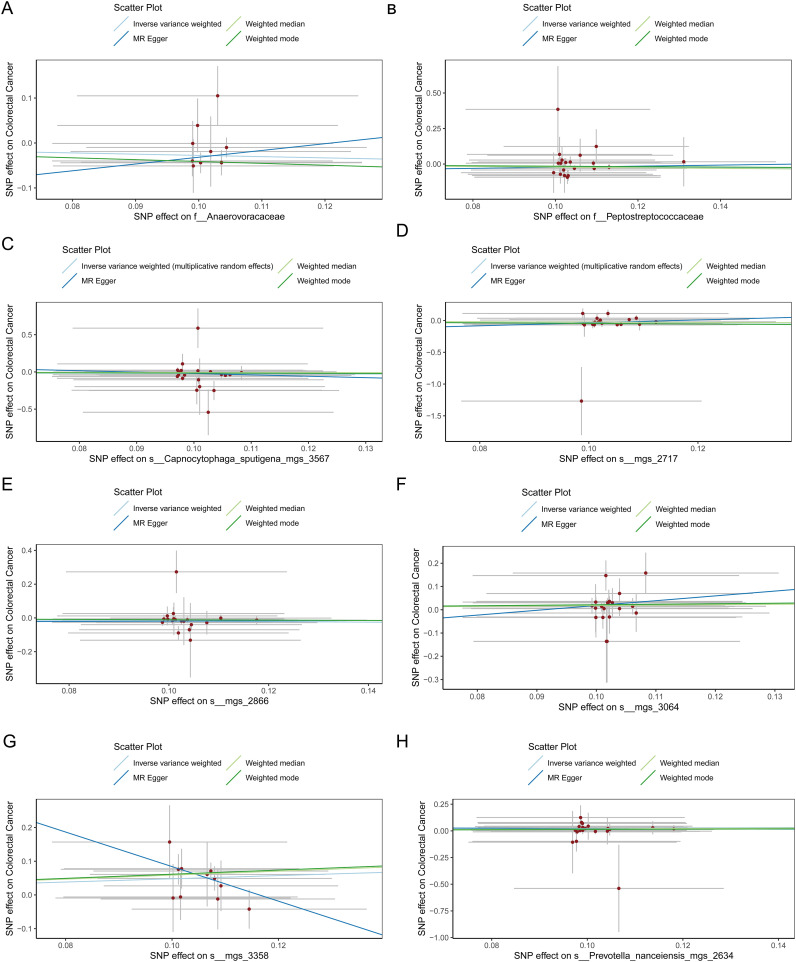
**(A)** Scatter plot depicting the relationship between single nucleotide polymorphisms (SNPs) effect on the family Anaerovoracaceae and their corresponding effect on colorectal cancer. **(B)** Scatter plot depicting the relationship between single nucleotide polymorphisms (SNPs) effect on the family Peptostreptococcaceae and their corresponding effect on colorectal cancer. **(C)** Scatter plot depicting the relationship between single nucleotide polymorphisms (SNPs) effect on the species Capnocytophaga sputigena_mgs_3567 and their corresponding effect on colorectal cancer. **(D)** Scatter plot depicting the relationship between single nucleotide polymorphisms (SNPs) effect on the species mgs_2717 and their corresponding effect on colorectal cancer. **(E)** Scatter plot depicting the relationship between single nucleotide polymorphisms (SNPs) effect on the species mgs_2866 and their corresponding effect on colorectal cancer. **(F)** Scatter plot depicting the relationship between single nucleotide polymorphisms (SNPs) effect on the species mgs_3064 and their corresponding effect on colorectal cancer. **(G)** Scatter plot depicting the relationship between single nucleotide polymorphisms (SNPs) effect on the species mgs_3358 and their corresponding effect on colorectal cancer. **(H)** Scatter plot depicting the relationship between single nucleotide polymorphisms (SNPs) effect on the species Prevotella nanceiensis_mgs_2634 and their corresponding effect on colorectal cancer.

**Figure 1** provides a comprehensive overview of the entire MR analysis process. Prior to further analysis, SNPs affected by linkage disequilibrium and those indicative of weak instrumental variables were removed. Ultimately, 25,488 SNPs associated with the salivary microbiome and 26,146 SNPs linked to the tongue microbiome were retained for analysis—with a significance threshold of p < 1x10^-5. The F-statistic ranged from 19.601 to 56.906, with all SNPs exceeding the threshold of 10, thus indicating no evidence of weak instrument bias (see **Supplementary Table 1**). Based on the inverse variance-weighted (IVW) MR analysis with a significance threshold of p < 0.05, a total of 161 taxa were initially identified as having a causal association with colorectal cancer. A comprehensive overview of these results is visually represented in the volcano plot (see **Figure 2**; **Supplementary Table 2**). Following FDR correction for multiple testing, 19 taxa were ultimately determined to exhibit a causal impact on colorectal cancer, including 11 taxa from tongue samples and 9 from saliva samples. In saliva samples, the taxa RUG343 and Streptococcus_umgs_2425 demonstrated protective effects against colorectal cancer, with odds ratios (ORs) of 0.817 (95% confidence interval [CI]: 0.704–0.949; p = 0.008) and 0.797 (95% CI: 0.700–0.909; p = 0.001), respectively. Conversely, taxa such as HOT-345_umgs_976 and W5053_sp000467935_mgs_712 were associated with an increased likelihood of negative health outcomes, with ORs of 1.210 (95% CI: 1.066–1.373; p = 0.003) and 1.183 (95% CI: 1.007–1.391; p = 0.041), respectively. Scatter plots illustrating the associations between individual SNPs and the outcomes for these taxa can be found in **Figure 3**, while a forest plot detailing the MR analysis results for colorectal cancer risk across different taxa is presented in **Figure 4**. In tongue samples, taxa such as Campylobacter_A_umgs_3358 and HOT-345_umgs_3064 showed significant negative associations with beneficial health outcomes, with ORs of 1.614 (95% CI: 1.217–2.141; p = 0.001) and 1.242 (95% CI: 1.063–1.451; p = 0.006), respectively. In contrast, increased abundance of the Anaerovoracaceae family was negatively correlated with health, exhibiting an OR of 0.759 (95% CI: 0.625–0.922; p = 0.005). Corresponding scatter plots showing the relationships between each SNP and the outcome for these taxa are displayed in **Figure 5**, with a forest plot summarizing the MR results and ORs for colorectal cancer across different taxa in **Figure 6**. For each positive result, the corresponding funnel plots and forest plots can be found in **Supplementary Files 1, 2**, respectively. Except for s:unclassified_mgs_2717, s:Capnocytophaga_sputigena_mgs_3567, and s:unclassified_mgs_389, the MR-Egger regression intercepts demonstrated no significant deviations from zero, indicating a lack of horizontal pleiotropy across most taxa (all intercepts > p 0.05), as detailed in **Supplementary Table 3**. The MR-PRESSO analysis further supported these findings, with all examined taxa showing no evidence of outliers, affirming the robustness of the data (refer to **Supplementary Table 4**). Moreover, Cochran’s Q test highlighted some heterogeneity specifically for s:mgs_389, s:mgs_2717, and s:Capnocytophaga_sputigena_mgs_3567, with Q-values falling below 0.05, suggesting variability in the effects across these taxa (see **Supplementary Table 5**). The leave-one-out analysis revealed consistent causal estimates across the different oral microbiome taxa, with no single SNP disproportionately influencing the overall results related to colorectal cancer (details in **Supplementary Table 6**; **Supplementary File 1**). The Steiger directionality test also found no evidence of a causal relationship between these diseases and the specific oral microbial taxa, as documented in **Supplementary Table 7**.

### 3.3 Genes and functionality

**Figure 7:**
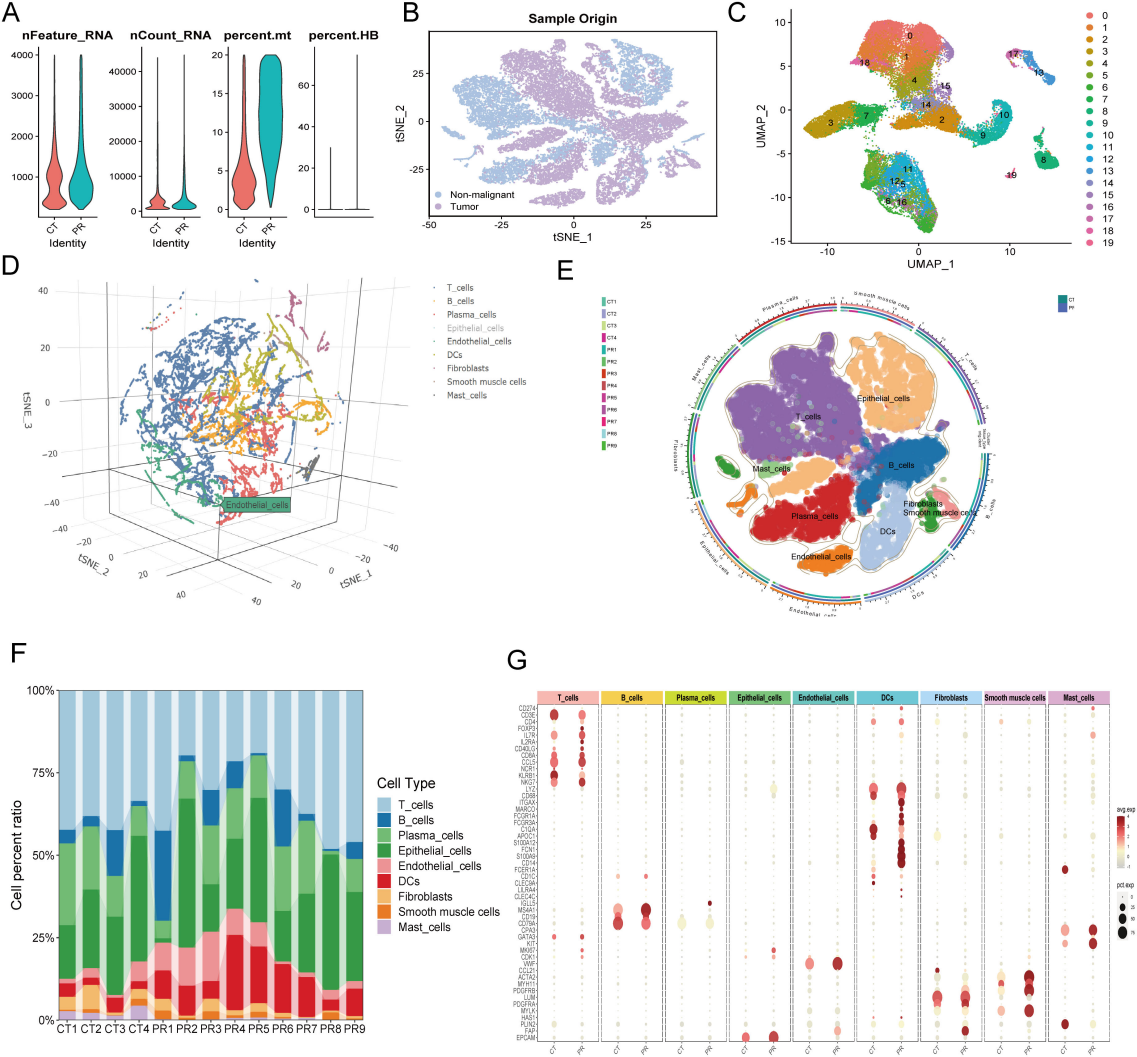
**(A)** Quality control metrics distribution for CT and PR samples showing nFeature_RNA, nCount_RNA, percent.mt, and percent.HB. **(B)** t-SNE visualization displaying single-cell sample origin, with blue indicating non-malignant cells and purple indicating tumor cells. **(C)** UMAP plot displaying 20 distinct clusters from single-cell RNA sequencing, each identified by a unique color. **(D)** Three-dimensional t-SNE plot illustrating the distribution of various cell types identified in the single-cell RNA sequencing data. **(E)** UMAP diagram showing diverse cell types and their distribution across different patient samples, delineated by color coding for cell types and an outer ring for sample origins. **(F)** Stacked bar chart representing the cell type composition across different patient samples (CT1-CT4, PR1-PR9). Each color in the chart corresponds to a specific cell type such as T cells, B cells, plasma cells, among others, illustrating their relative abundance in each sample. **(G)** Dot plot showing the expression levels of key markers across various cell types identified in the samples. Dot size indicates the percentage of cells expressing the gene (pct.exp), and color intensity reflects the average expression level (avg.exp).

The correspondence between SNPs and genes, along with their functions, is detailed in **Supplementary Table 8**. **Figure 7B** presents a circular plot derived from the enrichment analysis of gene interactions with biological pathways. This figure highlights the associations of key genes (e.g., BCL2, AOX1, CBX4, PHACTR3) with multiple biological pathways. Each color in the plot represents a different biological pathway, and the connections between genes and pathways illustrate the involvement of these genes in specific pathways (details in **Supplementary Table 9**). The JAK-STAT signaling pathway is linked to all four genes, suggesting its central role in the biological processes regulated by these genes. Notably, the connections between BCL2 and AOX1 with the JAK-STAT pathway are particularly significant, indicating that these genes might play roles in cellular signaling via this pathway. The apoptosis pathways are primarily associated with BCL2 and PHACTR3, consistent with BCL2’s function as a major anti-apoptotic protein, while the involvement of PHACTR3 might suggest novel regulatory mechanisms. Tyrosine metabolism shows a strong association with AOX1, reflecting its role in amino acid metabolism. Additionally, both CBX4 and PHACTR3 also exhibit associations with the tyrosine metabolism pathway, albeit to a lesser extent. Niacin and nicotinamide metabolism, as well as tryptophan metabolism, are enriched in AOX1 and PHACTR3, indicating that these metabolic pathways might play significant roles in regulating the functions of these genes. Furthermore, the plot reveals enrichment for pathways such as protein phosphatase binding, phosphatase binding, SUMO ligase activity, death domain binding, and SUMO binding. Notably, CBX4 is significantly enriched in SUMO-related pathways, potentially elucidating its role in protein modification and signal transduction. Overall, this enrichment diagram effectively reveals the participation of oral microbiome-associated genes in multiple key biological pathways, providing clues for further research into the roles of these genes in biological processes. These results emphasize the complex interplay of intracellular signaling and metabolic pathways in cellular functionality.

### 3.4 Single-cell global analysis results

**Figure 8:**
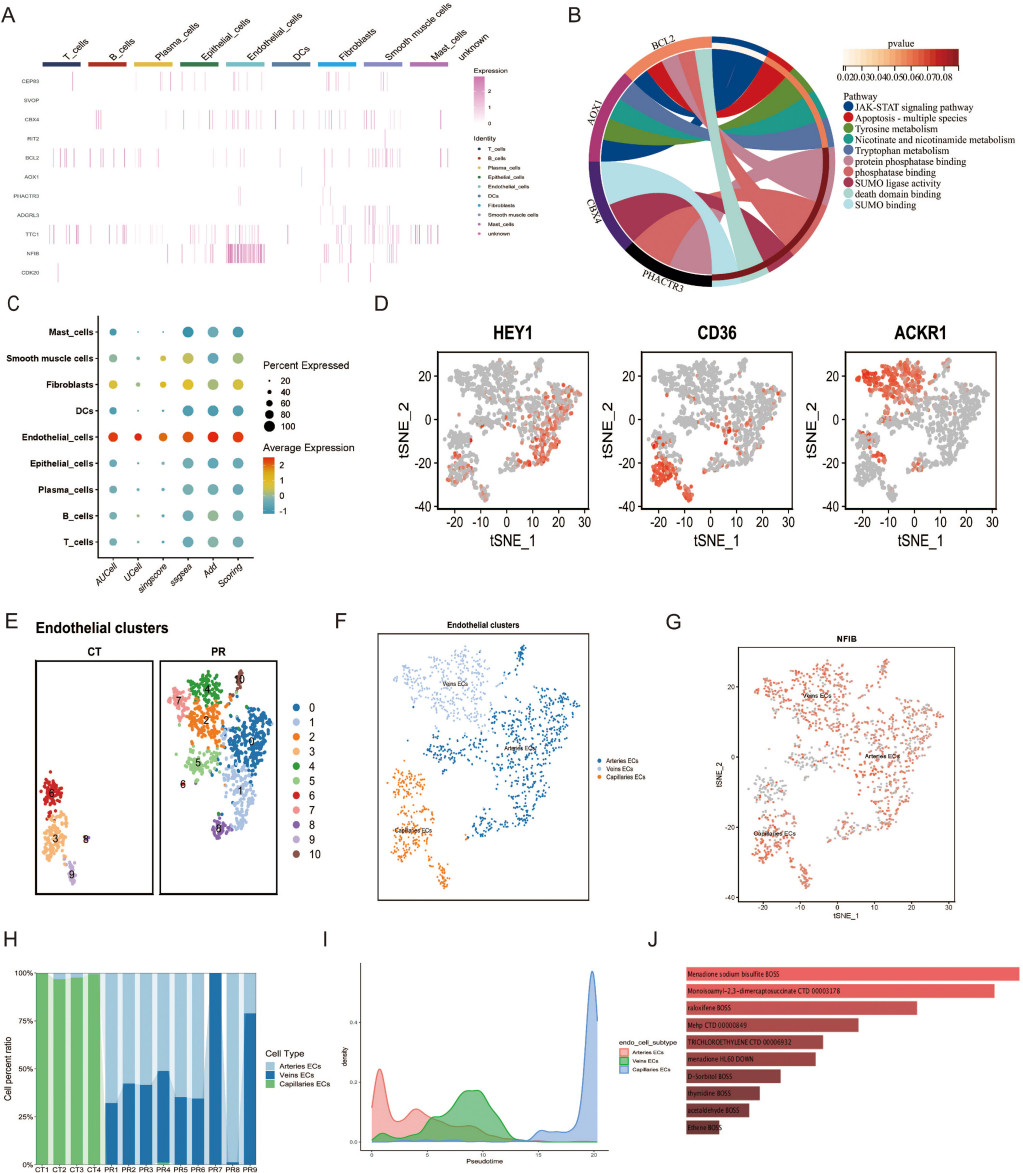
**(A)** Heatmap displaying the expression levels of selected genes across various cell types. **(B)** Chord diagram illustrating the functional pathways associated with selected genes. **(C)** Dot plot illustrating the scoring of a gene set across various cell types, based on different scoring metrics such as AUCell, UCell, singscore, ssgsea, and Add Scoring. Dot size indicates the percent of cells expressing the gene, and color scale reflects the scoring intensity. **(D)** t-SNE plots highlighting the expression of marker genes (HEY1, CD36, ACKR1) used for subtyping endothelial cells in single-cell data. Cells expressing each gene are highlighted in red, showing their distribution within the endothelial cell subpopulations. **(E)** UMAP plot displaying epithelial cell clusters across two sample groups, CT and PR. Each cluster is distinguished by a unique color, representing 11 distinct cell populations. **(F)** UMAP visualization of endothelial cell subtypes, categorized into arteries ECs (blue), veins ECs (light blue), and capillaries ECs (orange), demonstrating the distinct spatial distribution of these subtypes. **(G)** Feature plot of the NFIB gene expression across the endothelial cell subtypes in a t-SNE plot. Each dot represents a cell, with color intensity indicating levels of NFIB expression in arteries ECs, veins ECs, and capillaries ECs. **(H)** Stacked bar chart showing the distribution of endothelial cell subtypes (arteries ECs, veins ECs, capillaries ECs) across different patient samples, displayed as CT1 to CT4 and PR1 to PR9. **(I)** Density plot representing the pseudotime trajectory analysis of endothelial cell subtypes, illustrating the developmental progression of arteries ECs, veins ECs, and capillaries ECs. **(J)** Bar chart displaying drugs predicted to target specific genes identified through Mendelian randomization analysis from endothelial cell subtypes, with the bar length indicating the strength of the predicted association based on the analysis.

Initially, quality control was performed on the single-cell data, with **Figure 7A** depicting the distribution of cells across several QC parameters, including RNA feature numbers (Feature_RNA), ribonucleic acid molecule counts (nCount_RNA), mitochondrial gene expression ratio (percent.mt), and hemoglobin gene expression ratio (percent.HB). Violin plots illustrate significant differences among various cell types in these parameters, revealing heterogeneity and cell-specific characteristics within the samples. Subsequently, a t-SNE technique provided a two-dimensional spatial distribution of cell samples, differentiating between tumor and non-tumor cells. **Figure 7B** offers an intuitive view of the cellular composition within the tumor microenvironment, displaying fundamental differences in cell components between tumors and surrounding tissues. Clustering of colorectal cancer single-cell data using the UMAP algorithm identified eight cell populations (**Figure 7C**), including T cells, B cells, plasma cells, epithelial cells, endothelial cells, fibroblasts, smooth muscle cells, and mast cells. This detailed clustering underscores the diversity of complex cell types and states at the single-cell level. **Figure 7D** presents a three-dimensional t-SNE scatter plot labeling various cell types, such as T cells, B cells, plasma cells, epithelial cells, and endothelial cells. This plot demonstrates the spatial relationships among cell types within the samples, providing a foundation for further analysis of intercellular interactions. A pie chart (**Figure 7E**) further subdivides the cell distribution in UMAP, where each segment represents a specific cell subtype, aiding in the identification of cellular compositions and functional regions within the microenvironment. A stacked bar graph (**Figure 7F**) shows the proportions of cell types across different patient samples, revealing the relative abundance of cell types within each sample and comparing cell composition under different samples or treatment conditions. **Figure 7G**, through a scatter plot, displays the expression of key genes across cell types. The size and color depth of the dots reflect the abundance and statistical significance of gene expression, providing molecular-level evidence for understanding the functional states and interactions of various cell types. A critical heatmap (**Figure 8A**) illustrates the expression of selected genes across different cell types. This heatmap depicts the variance in gene expression levels among cell groups, highlighting specific genes like NFIB that are highly expressed in endothelial cells. In summary, these results not only reveal cellular heterogeneity within the tumor microenvironment but also provide valuable data resources for further studying the cellular dynamics and interactions during tumor progression. These findings are significant for deepening the understanding of tumor biological mechanisms and developing targeted therapeutic strategies.

### 3.5 Single-cell analysis of endothelial cell populations

We utilized Mendelian Randomization (MR) to infer specific gene sets from SNP data, subsequently assessing their expression at the single-cell level through various scoring metrics, including AUCell, UCell, singscore, ssgsea, and Add Scoring. **Figure 8C** illustrates the gene set scoring across various cell types—including mast cells, smooth muscle cells, fibroblasts, dendritic cells, endothelial cells, epithelial cells, plasma cells, B cells, and T cells—demonstrating the percentage and average intensity of gene expression within different cell types. Notably, endothelial cells exhibited significant gene expression activity, underscoring their importance in our study. Further subclassification of endothelial cells distinguished between arterial endothelial cells (Arteries ECs), venous endothelial cells (Veins ECs), and capillary endothelial cells (Capillaries ECs). **Figures 8E, F** display the UMAP analysis utilized to differentiate these endothelial subpopulations. These diagrams, through color coding, provide an in-depth view of the spatial distribution of various subgroups, offering insights into the diversity of cell types and the complexity of the microenvironment. Classification was based on spatial distributions derived from t-SNE methodology, where differential expression of specific genes such as HEY1, CD36, and ACKR1 among the endothelial subtypes served as the basis for classification (**Figure 8D**). A feature plot of the NFIB gene was employed to elucidate its expression patterns, providing clues to its functional roles across different cell types. **Figure 8H** presents a stacked bar graph showing the proportions of endothelial cell subtypes across different patient samples, facilitating comparisons of cellular composition among samples. A density plot (**Figure 8I**) illustrates the pseudotemporal analysis results of the endothelial cell subgroups, displaying the distribution of arterial endothelial cells, venous endothelial cells, and capillary endothelial cells along the predicted developmental timeline. Arterial endothelial cells displayed a density peak at earlier hypothetical time points, while capillary endothelial cells showed peaks at later time points, suggesting that these cell types may undergo different developmental paths and timings. The distribution of venous endothelial cells was relatively uniform, indicating a steady presence throughout the time series. Through such analyses, we observed the dynamic changes of different endothelial cell types during tumor progression, revealing their potential developmental trajectories and functional evolution. This provides vital insights into how endothelial cells adapt and evolve within the tumor microenvironment.

### 3.6 Drug prediction

In our study, Mendelian Randomization was applied in conjunction with the Drug Signatures Database (DsigDB) to predict potential therapeutics targeting genes identified from top SNPs. The following drugs demonstrated significant associations with specific genes: Menadione Sodium Bisulfite (p-value = 3.91×10^-4, OR = 80.94), predicted by genes BCL2 and AOX1; Monosomy1-2-3-dimercaptosuccinate (p-value = 1.74×10^-4, OR = 71.12), associated with genes BCL2 and CDK20; Raloxifene (p-value = 1.01×10^-4, OR = 47.86), also predicted by BCL2 and AOX1; MehP (p-value = 0.00187, OR = 35.45) and D-Sorbitol (p-value = 0.00400, OR = 23.87), both linked to BCL2 and AOX1; Trichloroethylene (p-value = 0.00265, OR = 29.64), affecting NFIB and BCL2; Menadione (p-value = 0.00285, OR = 28.55), impacting BCL2 and CPEB3; Thymidine (p-value = 0.00491, OR = 21.45), associated with BCL2 and CDK20; Acetaldehyde (p-value = 0.00544, OR = 20.32) and Ethene (p-value = 0.00727, OR = 17.42), the former related to BCL2 and AOX1, and the latter to RIT2 and BCL2. These findings provide robust molecular evidence for drug development targeting specific cell subtypes within the tumor microenvironment, emphasizing the importance of gene-targeted drug screening.

### 3.7 Molecular docking

**Figure 9:**
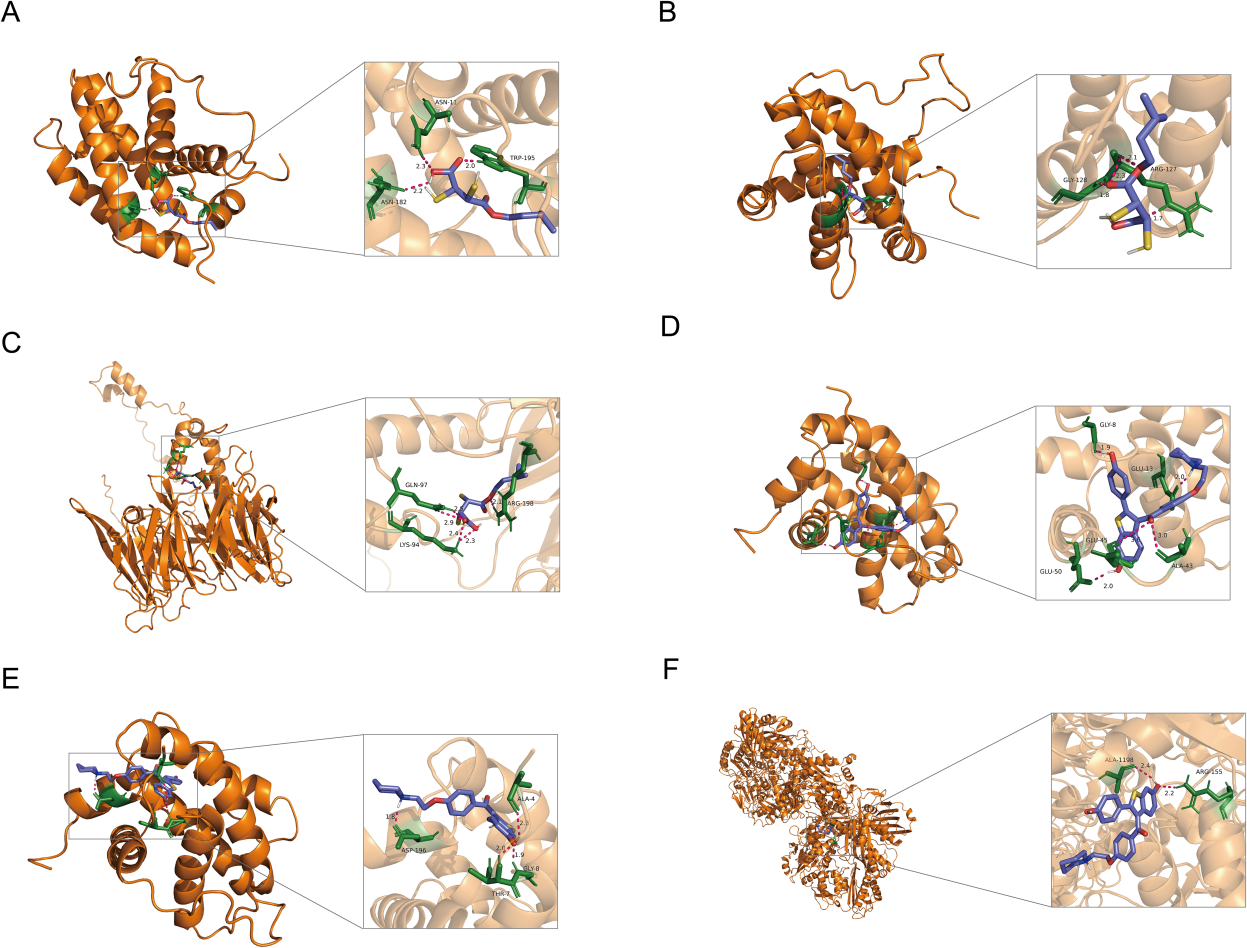
**(A)** BCL2 (PDB ID: 1G5M) docked with Monoisopropyl-2,3-dimercaptosuccinate. **(B)** BCL2 (PDB ID: 1GJH) docked with Monoisopropyl-2,3-dimercaptosuccinate. **(C)** CDK20 (no PDB ID) docked with Monoisopropyl-2,3-dimercaptosuccinate. **(D)** BCL2 (PDB ID: 1G5M) docked with Menadione sodium bisulfite. **(E)** BCL2 (PDB ID: 1GJH) docked with Menadione sodium bisulfite. **(F)** AOX1 (PDB ID: 8EMT) docked with Menadione sodium bisulfite.

In this study, we conducted a detailed analysis of the binding affinities and interactions between six different small molecules and their target proteins using molecular docking methods (**Figures 9A–F**). We observed that the binding affinities of BCL2 protein with two different ligands (PDB IDs: 1G5M and 1GJH) were -7.91 kcal/mol and -10.11 kcal/mol, respectively, indicating strong binding capabilities. This robust affinity is attributed to multipoint hydrogen bonding and van der Waals interactions with key residues such as Gly181, Ser184, Arg143, and Thr141, ensuring efficient and stable ligand binding at the active site. In contrast, the binding affinity with CDK20 protein was lower (-2.17 kcal/mol), primarily due to relatively weaker interactions with residues Lys33 and Ala35. Additionally, the binding affinity of AOX1 protein with its ligand was -4.08 kcal/mol, characterized by moderate binding strength through hydrogen bonds with the residue Arg400. Detailed analyses of these binding characteristics and interactions have been visualized using Pymol 2.6.0 software and are thoroughly documented in the **Supplementary Files** of the study (**Supplementary File 4**; **Table 2**), providing a crucial molecular basis for future drug development and optimization.

**Table 2:** This table presents molecular docking results showing the interaction of various drugs with specific targets.

Target	PDB ID	Pubchem iD	Binding energy	Drug
BCL2	1G5M	164448	−3.56	Monoisoamyl-2,3-dimercaptosuccinate
BCL2	1GJH	164448	−3.26	Monoisoamyl-2,3-dimercaptosuccinate
CDK20	–	164448	−2.17	Monoisoamyl-2,3-dimercaptosuccinate
BCL2	1G5M	5035	−7.91	Menadione sodium bisulfite
BCL2	1GJH	5035	−10.11	Menadione sodium bisulfite
AOX1	8EMT	5035	−4.08	Menadione sodium bisulfite

It includes the Protein Data Bank (PDB) ID, PubChem ID of the drugs, and their binding energy values indicating the strength of interaction. The table lists multiple interactions for BCL2, CDK20, and AOX1 targets, with corresponding drugs such as Monoisamyl-2,3-dimercaptosuccinate and Menadione sodium bisulfite, highlighting potential therapeutic implications.

## 4 Discussion

The investigation into the interrelationship between the oral microbiome and colorectal cancer reveals significant gaps in existing research. Compared to the extensively studied gut microbiome, research on the oral microbiome is relatively scarce. This disparity in research not only limits our comprehensive understanding of the relationship but also hinders the discovery of potential preventative and therapeutic approaches. Traditional microbiome studies, reliant on sequencing technologies, face limitations due to technological and sampling constraints, resulting in significant heterogeneity in findings. Our research aims to explore the causal relationship between the oral microbiome and colorectal cancer, emphasizing the role of oral microbes in the oncogenic process. Moreover, our study delves deeper into colorectal cancer at the single-cell level, identifying significant expression of oral microbiome-related genes in colorectal cancer cells, which led to drug prediction and molecular docking analysis. This innovative approach enhances the robustness of our results and advances precise inference of the causal relationship between microbiomes and colorectal cancer.

Utilizing summary statistics from GWAS meta-analyses of oral microbiomes and colorectal cancer provided by the MiBioGen Consortium, we conducted a two-sample MR analysis to evaluate their causal relationship. The bidirectional MR analysis aims to comprehensively understand the complex interactions between the oral microbiome and colorectal cancer. This method not only assesses how the microbiome influences the onset of colorectal cancer but also systematically considers reverse causality, thereby revealing potential changes in the oral microbiome induced by colorectal cancer. The study highlights that controlling for confounders through common genetic factors ensures reliable causal inference.

In our study, we identified various oral microbial taxa and analyzed their correlation with colorectal cancer (CRC). By examining saliva and tongue samples, we found that the enrichment of Absconditabacterales (in saliva), Campylobacter_A (in tongue), Prevotella (in tongue), and Catonella (in saliva) is associated with an increased risk of CRC, suggesting these microbes may act as risk factors. Conversely, the presence of Capnocytophaga, Gemella (in both saliva and tongue samples), Anaerovoracaceae (in tongue), Peptostreptococcaceae (in tongue), Streptococcus, Centipeda, and Lachnospiraceae (in saliva) appears to exert a protective effect, potentially inhibiting cancer development. These findings not only enhance our understanding of the role of oral microbial diversity in the onset of CRC but may also provide a theoretical basis for developing microbiome-based preventive or therapeutic strategies. Campylobacter_A is a principal cause of bacterial colon infections globally and has shown a robust capacity to survive under various stressful conditions by interacting with certain intestinal pathogens (Kim et al., 2020). Studies indicate that Campylobacter jejuni promotes the occurrence of colorectal tumors through multiple mechanisms. Notably, it produces a genotoxin known as cytolethal distending toxin (CDT), which has been proven to cause DNA damage. Animal model studies suggest that introducing C. jejuni into a germ-free environment can significantly alter the gut microbiota and enhance tumor formation, indicating complex interactions between this bacterium, the gut microbiome, and the carcinogenic process (He et al., 2019). Further human clinical data and animal studies reveal higher abundance of Campylobacter species, including C. jejuni, in CRC tissues compared to normal tissues, suggesting that the presence of Campylobacter may influence the development of CRC by altering microbial community structures (Costa et al., 2022). Additionally, a comprehensive review of the mucosal microbiota of CRC patients found that Campylobacter and several other bacteria are more common in cancerous tissues compared to healthy controls, supporting the hypothesis that Campylobacter and specific bacteria are related to CRC pathology (Butzler, 1982). In a pioneering study conducted in Iran, researchers analyzed the overall microbiome of saliva and fecal samples from CRC patients and healthy controls. The study identified significant changes in the abundance of certain bacterial genera, including Catonella, potentially linking these bacteria to CRC development. Furthermore, differences in microbial diversity between the saliva samples of healthy controls and CRC patients suggest that oral microbiota may be relevant for the early detection and prevention of CRC (Rezasoltani et al., 2022). Research has also highlighted that Prevotella abundance is significantly higher in Indian populations compared to Western populations, suggesting a relationship with dietary habits and gut health status (Vishnu Prasoodanan et al., 2021). Moreover, the presence of Prevotella in CRC patients is linked to prognosis, with specific species’ relative abundance in pre-operative fecal samples correlating with clinical outcomes, serving as potential prognostic biomarkers (Huh et al., 2022). Further, Prevotella plays a role in modulating immune responses, with studies showing a positive correlation between Prevotella and the expression of intestinal inflammatory markers like IL-9, which may promote the pathological process of CRC (Niccolai et al., 2020). Despite these microbial groups showing potential to increase the risk of CRC, our research has also revealed other taxa that may inhibit the progression of colorectal cancer. The Anaerovoracaceae family, for instance, may influence the response to cancer immunotherapy by affecting T-cell function (Blumenberg et al., 2021). These preliminary findings suggest that the gut microbiome, including the Anaerovoracaceae, could play a role in the tumor immune environment, thereby impacting the efficacy of immunotherapies, consistent with our results. Lachnospiraceae may play a significant role in the development of CRC. The negative correlation of this family with CRC risk offers critical insights into preventing and treating CRC by regulating the gut microbiota and controlling inflammatory factors (Ma et al., 2024). Specifically, bacteria from the Lachnospiraceae family are thought to reduce colorectal tumor formation by altering the tumor immune microenvironment. For instance, studies suggest that fiber-rich Lachnospiraceae may reduce CRC incidence by modulating immune responses (Almeida et al., 2021). Additionally, the metabolic products of Lachnospiraceae bacteria, particularly short-chain fatty acids (SCFAs) like butyrate, play roles in gut health and cancer prevention. SCFAs promote the health of colon epithelial cells and have anti-inflammatory effects, which are crucial in the anticancer process (Coker et al., 2022). Existing research also shows that the Streptococcus genus is associated with the development and progression of CRC (Wang et al., 2021; Quaglio et al., 2022). Findings reveal geographical and racial differences in the association rates between S. bovis/gallolyticus and colorectal tumors. Additionally, the link between S. bovis/gallolyticus-related colonic lesions and bloodstream infections (bacteremia/endocarditis) suggests a unique pathway for these bacteria to enter the bloodstream via the portal venous system (Alozie et al., 2015). In a prospective study targeting low-income and African-American populations, analyzing the oral microbiome and subsequent CRC risk revealed that several bacterial taxa, including the Streptococcus genus, were associated with reduced CRC risk, although these associations were not significant after multiple testing corrections (de Almeida et al., 2018).

To better understand the biological functions of these genes in disease, we conducted GO and KEGG analyses. We discovered that the JAK-STAT signaling pathway, an essential intracellular signaling system, is ubiquitous across a variety of organisms, from humans to fruit flies. This pathway is primarily initiated by extracellular signals such as cytokines and growth factors, which transmit signals through receptors on the cell membrane to the intracellular Janus kinase (JAK), subsequently triggering the phosphorylation of signal transducer and activator of transcription (STAT). Phosphorylated STAT proteins form dimers that regulate the expression of specific genes by entering the nucleus (Hu et al., 2023). The JAK-STAT pathway plays a crucial role in cellular processes such as growth, differentiation, apoptosis, and immune regulation. For instance, it is vital in determining the fate of T-helper cells, influencing the differentiation of various cell types such as Th1, Th2, Th17, and regulatory T cells (Seif et al., 2017). However, aberrant activation of this pathway is often associated with various diseases, particularly in cancer. For example, the activation of STAT3 is closely linked to the occurrence of multiple tumors, the formation of drug resistance, and the maintenance of cancer stem cells (Rah et al., 2022). Therefore, the JAK-STAT signaling pathway is a significant target in cancer therapy, and a deeper understanding of it could lead to the development of more effective treatment methods.

In mammals, the intrinsic pathway of apoptosis primarily revolves around mitochondria, involving several key proteins such as members of the Bcl-2 family, which maintain cell survival by directly or indirectly inhibiting pro-apoptotic proteins (like BAK and BAX). When cells are overwhelmed by stress or developmental signals, this survival signal is overridden, triggering the initiation of apoptosis (Cavalcante et al., 2019). Additionally, studies have indicated that miRNAs also play a crucial role in regulating apoptosis-related genes, and any imbalance in these mechanisms could lead to the development of various diseases (Cavalcante et al., 2019). Apoptosis is not just a form of cell death; it involves numerous finely regulated molecular mechanisms, significantly impacting the understanding and treatment of various diseases.

Tyrosine metabolism is a complex biochemical process primarily occurring in the liver, involving multiple enzymes and metabolic pathways. In diseases like hepatocellular carcinoma, abnormalities in tyrosine metabolism can affect the regulation of the cell cycle and cellular proliferation. Studies have shown that the expression of tyrosine metabolism enzymes is decreased in hepatocellular carcinoma, closely associated with poor prognosis. Furthermore, abnormalities in tyrosine metabolism can also activate the cell cycle, promoting cell proliferation (Wang et al., 2022). Niacin and nicotinamide metabolism are crucial biochemical processes related to cellular energy production and repair. Studies have found that niacin and nicotinamide metabolism, through their metabolites such as NAD+ and 1-methyl nicotinamide, play a significant role in treating various diseases. For instance, nicotinamide mononucleotide (NMN), a vital precursor of NAD+, has been shown to combat aging and enhance cellular metabolic status (Song et al., 2023; Malik et al., 2024). Moreover, niacin and nicotinamide metabolism are also closely linked to the development of many diseases, such as diabetic peripheral neuropathy and the inflammatory processes in rheumatoid arthritis. Regulating this metabolic pathway can influence the progression and treatment outcomes of diseases (Malik et al., 2024; Ye et al., 2024). Tryptophan metabolism plays a key role in various physiological and pathological processes, particularly in digestive system tumors, including gastric and colorectal cancers. The expression levels of tryptophan and its metabolites are closely associated with the clinical characteristics of tumors. For example, in gastric cancer, increased expression of the tryptophan-metabolizing enzyme TDO2 is negatively correlated with tumor aggressiveness and overall patient survival (Ye et al., 2021; Yu et al., 2024). Additionally, tryptophan metabolism pathways, such as the kynurenine pathway, also play roles in regulating gut inflammation and brain health, demonstrating potential therapeutic applications (Chen et al., 2021; Roth et al., 2021; Michaudel et al., 2023).

In our study, we utilized the DsigDB database to predict potential drug candidates targeting genes identified through Mendelian randomization from top SNP data. Among these, the vitamin K derivative Menadione sodium bisulfite (MSB) has demonstrated potential value in cancer therapy. Existing research indicates that MSB can inhibit tumor cell growth by depleting the pool of acid-soluble thiols such as glutathione, particularly evident in mouse leukemia L1210 cells (Akman et al., 1985). Additionally, when used in combination with vitamin C, MSB significantly enhances the cytotoxicity against prostate cancer cells and can synergize with anticancer drugs like bortezomib to reduce toxicity and enhance antitumor effects (sodium ascorbate study). These studies suggest that MSB impacts cancer cell survival and proliferation through multiple mechanisms, warranting further exploration and validation of its application in cancer treatment (Astakhova et al., 2018). Other potential drug candidates include the heavy metal chelator Monoisomy1-2-3-dimercaptosuccinate, which may facilitate the removal of heavy metals from cells; Raloxifene, a selective estrogen receptor modulator that may influence endothelial cells via the estrogen receptor pathway; and MEHP, which could affect cellular behavior through endocrine disruption. Additionally, the industrial solvent Trichloroethylene may induce oxidative stress and cytotoxicity affecting cellular physiology, while Menadione acts by influencing redox reactions and pathways related to cellular apoptosis. D-Sorbitol, as an osmotic diuretic, may regulate intracellular osmotic pressure and carbohydrate metabolism; Thymidine affects cell cycles by impacting DNA synthesis; Acetaldehyde may induce DNA damage and stress responses; and Ethene could influence hormonal signaling and cellular response mechanisms (Janakiram et al., 2009; McGuire et al., 2012; Shih et al., 2023). These analytical results not only highlight the potential of drug screening based on single-cell data but also provide valuable insights for developing treatment strategies targeting specific cell subtypes within the tumor microenvironment. This study presents several notable advantages. Firstly, we utilized the latest genome-wide association study (GWAS) data related to the oral microbiome and employed Mendelian Randomization (MR) as a methodological approach to establish causative relationships. This method allows us to assess potential links between the components of the oral microbiome and colorectal cancer from a genetic perspective. Secondly, we innovatively integrated single-cell transcriptome analysis, precisely identifying endothelial cell populations through cell-specific scoring techniques. Enrichment analysis further revealed the functional characteristics of the gene sets we identified. Lastly, through drug prediction and molecular docking techniques, we explored several genes as potential therapeutic targets and predicted potential therapeutic drugs associated with these targets. This series of studies provides new insights into the development of personalized treatment strategies for colorectal cancer and offers preliminary candidate targets and drugs for future drug development.

However, several limitations of this study must be acknowledged. A significant concern in Mendelian Randomization studies is the possibility of horizontal pleiotropy, which could affect the accuracy of selecting instrumental variables. The composition of the oral microbiome can be influenced by multiple factors, including genetic background, lifestyle choices, dietary habits, and environmental factors, all of which could impact the outcomes of the study. Additionally, the instrumental variables used may only explain a small portion of the observed variability, necessitating further research to fully understand the complex changes in the oral microbiome. Moreover, our MR analysis focused predominantly on populations of Asian descent, meaning our results may not be generalizable to other ethnicities, such as those of European descent. Further research is needed to validate and extend our findings to other populations. Single-cell RNA sequencing (scRNA-seq) and drug prediction analyses offer tremendous potential for providing high-resolution cellular characteristics and identifying potential therapeutic strategies. However, several limitations of these methods must be acknowledged. Firstly, the quality and resolution of scRNA-seq data are highly dependent on sampling and technical factors, such as cell capture efficiency and sequencing depth. This dependency may result in underrepresentation of certain cell types or subpopulations. Additionally, the complexity of single-cell data analysis increases the difficulty of interpreting results, particularly in distinguishing between technical noise and biological significance. In the realm of drug prediction, although databases like DSigDB offer extensive gene-drug association information, these predictions require further experimental validation to confirm their efficacy and safety. The accuracy of prediction models is also limited by the coverage of existing datasets and the assumptions made by algorithms. Furthermore, the effects of drugs observed at the cellular level may not directly translate to *in vivo* outcomes. Hence, more *in vivo* studies and clinical trials are necessary to verify their practical application value.

Future research should expand to populations of different ethnic and geographical backgrounds to validate and generalize current findings, and collect larger sample sizes to improve statistical accuracy and result robustness. Longitudinal studies will reveal the temporal dynamics between the oral microbiome and colorectal cancer development. In-depth functional genomics and metabolomics studies will elucidate the specific mechanisms of the microbiome. Future studies should integrate multi-omics data and apply single-cell multi-omics and spatial transcriptomics technologies to depict interactions between the microbiome and host cells at higher resolution. These directions will deepen our understanding of the oral microbiome’s relationship with colorectal cancer and advance personalized medicine and precision therapy.

## 5 Conclusion

This study establishes the significant role of the oral microbiome in colorectal cancer (CRC) development through Mendelian Randomization (MR) analysis. We identified 19 bacterial taxa associated with CRC risk, with specific microbes showing both protective and harmful effects. Single-cell RNA sequencing highlighted key pathways, including JAK-STAT signaling, involved in CRC progression. Drug prediction and molecular docking identified potential therapeutics like Menadione Sodium Bisulfite and Raloxifene. These findings offer new insights and therapeutic targets for personalized CRC treatment strategies.

## Data Availability

The original contributions presented in the study are included in the article/**Supplementary Material**. Further inquiries can be directed to the corresponding author/s.
