# Supplementary figures and images for: Revealing the association between East Asian oral microbiome and colorectal cancer through Mendelian randomization and multi-omics analysis
Source: Front Cell Infect Microbiol. 2024 Sep 17;14:1452392. doi: 10.3389/fcimb.2024.1452392 (PMC11443854; doi:10.3389/fcimb.2024.1452392)

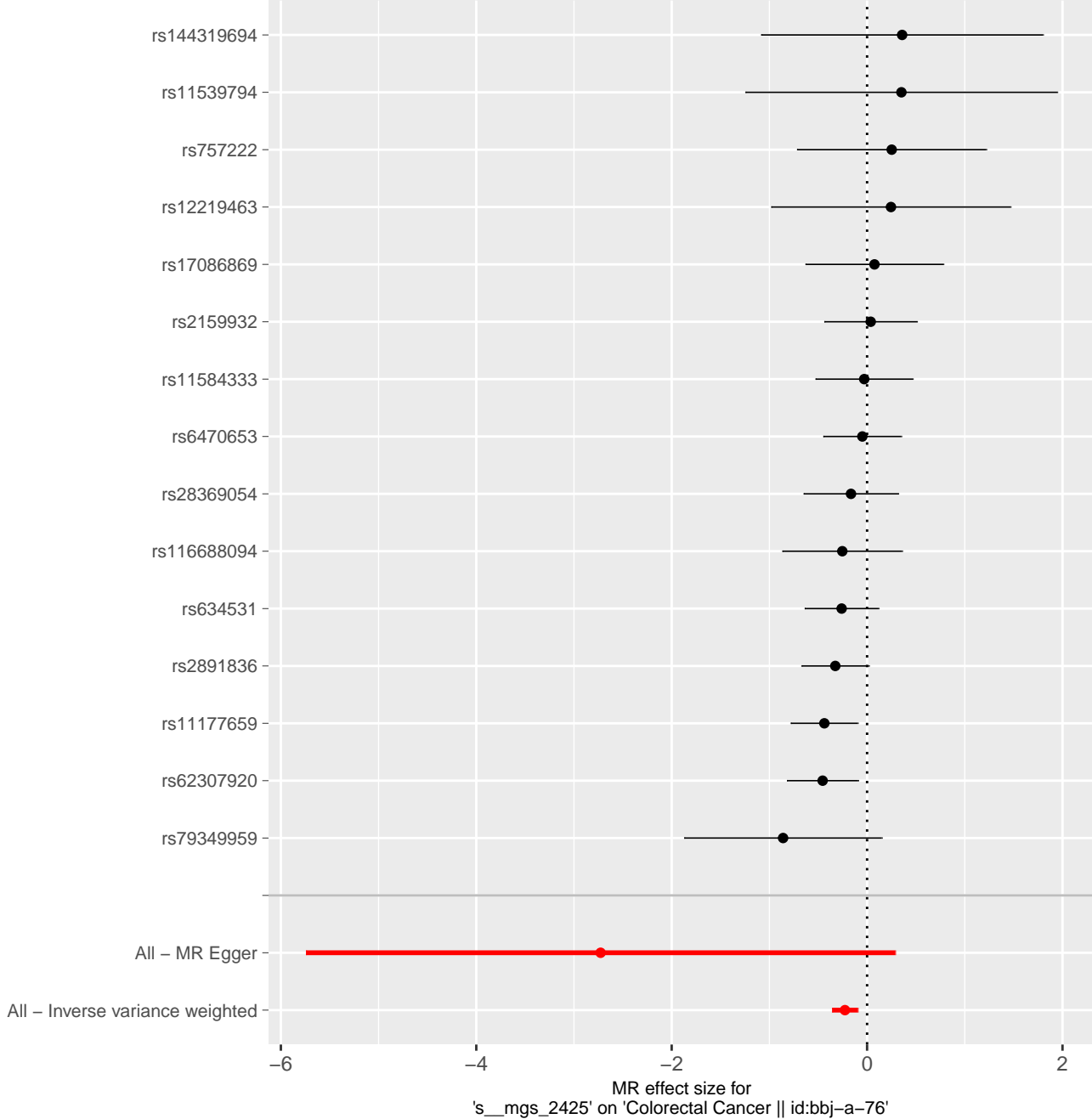

Supplement: Supplementary file 1 [file Supplementaryfile1.zip › Supplementary files 1 forest plot/saliva-pheno.1269.bbj-a-76.pdf]

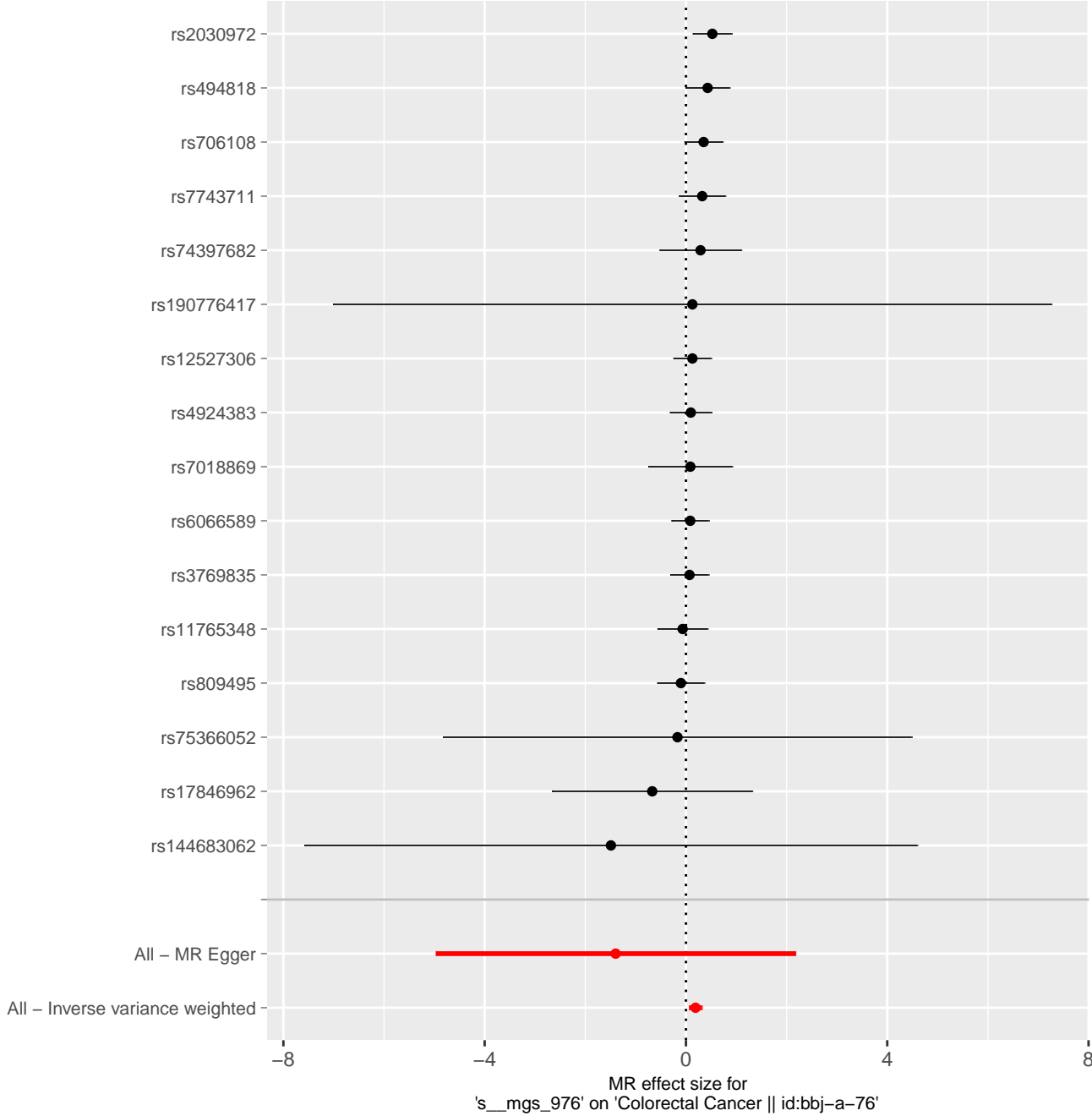

Supplement: Supplementary file 1 [file Supplementaryfile1.zip › Supplementary files 1 forest plot/saliva-pheno.1542.bbj-a-76.pdf]

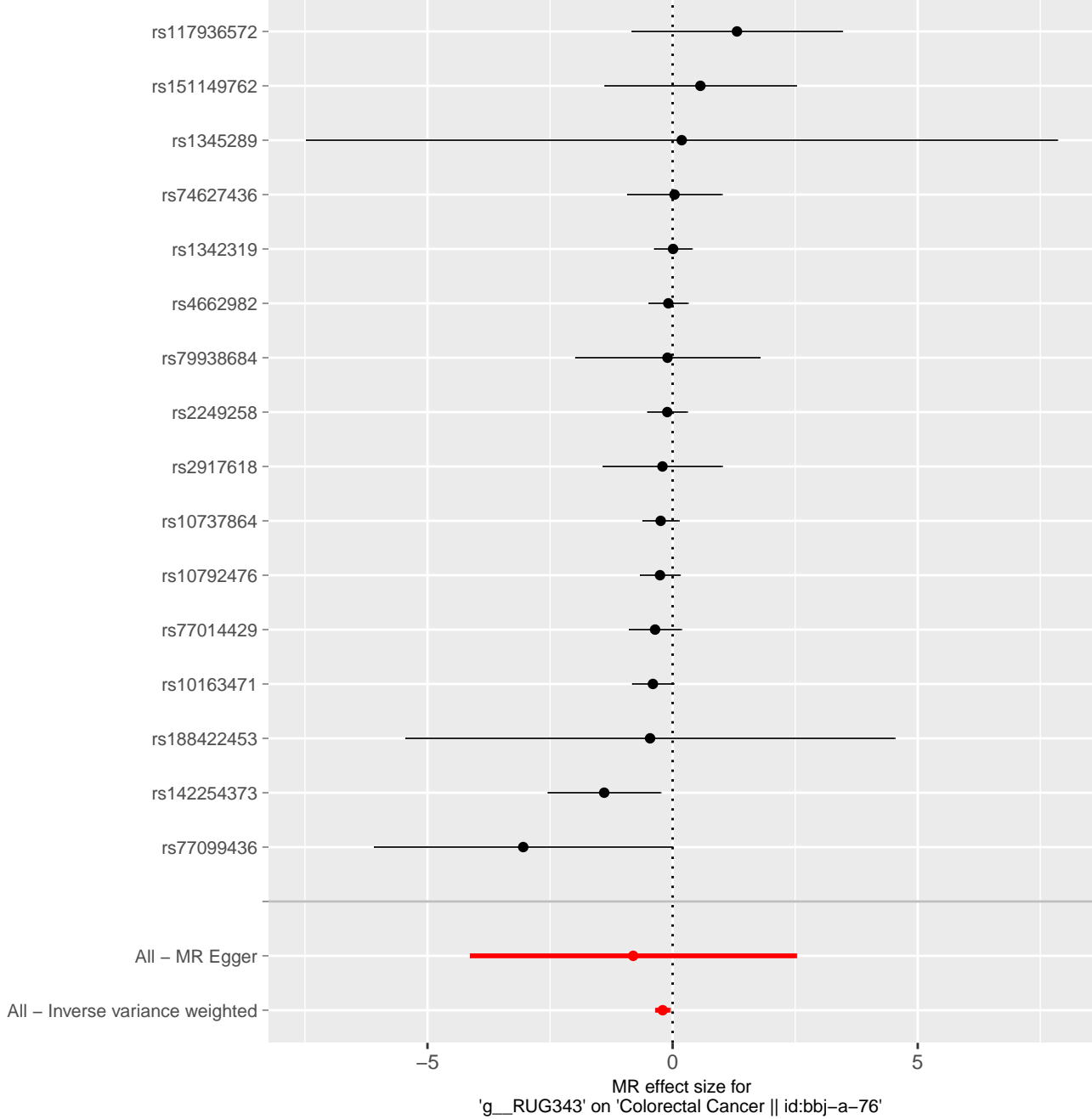

Supplement: Supplementary file 1 [file Supplementaryfile1.zip › Supplementary files 1 forest plot/saliva-pheno.160.bbj-a-76.pdf]

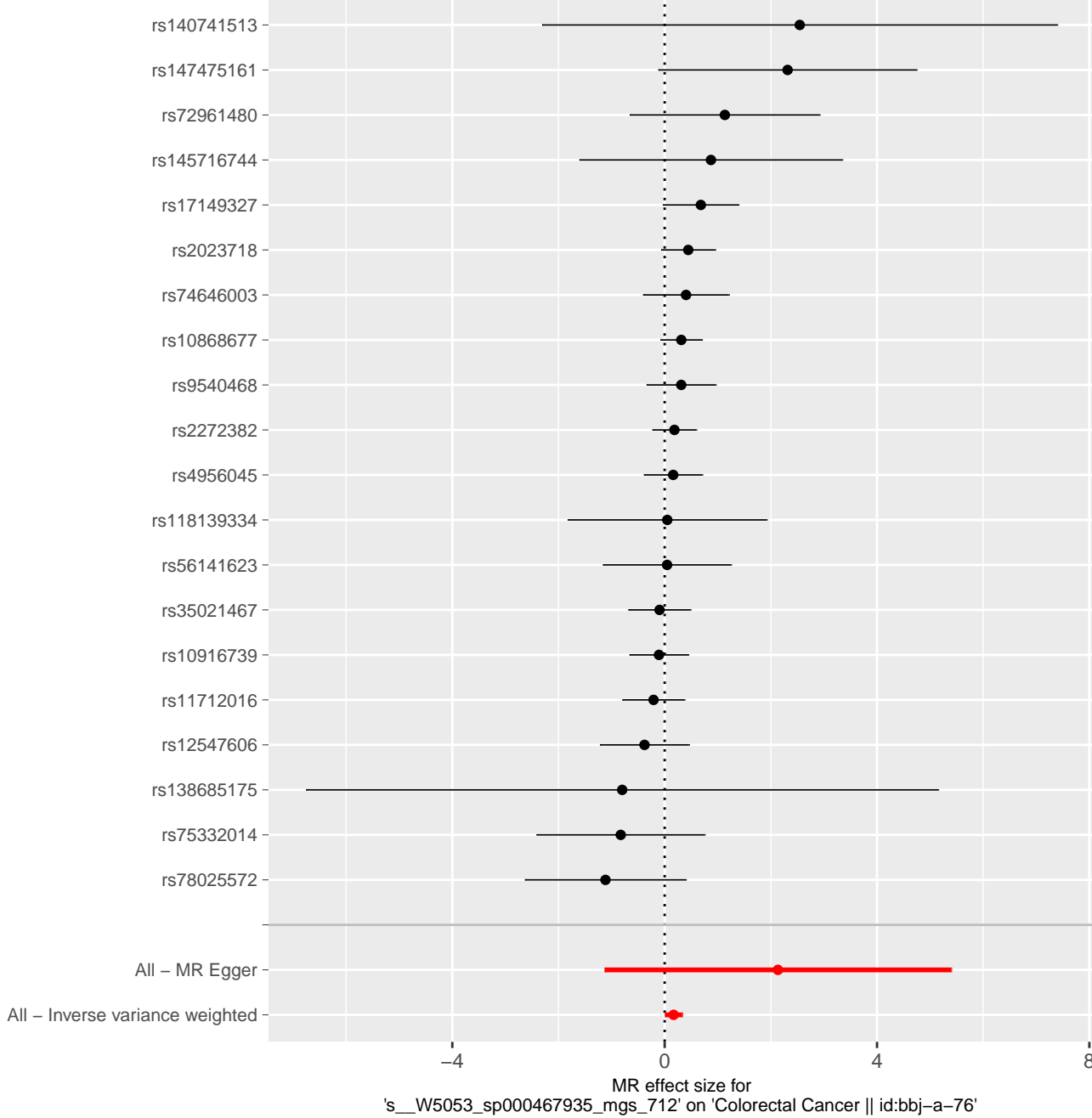

Supplement: Supplementary file 1 [file Supplementaryfile1.zip › Supplementary files 1 forest plot/saliva-pheno.2303.bbj-a-76.pdf]

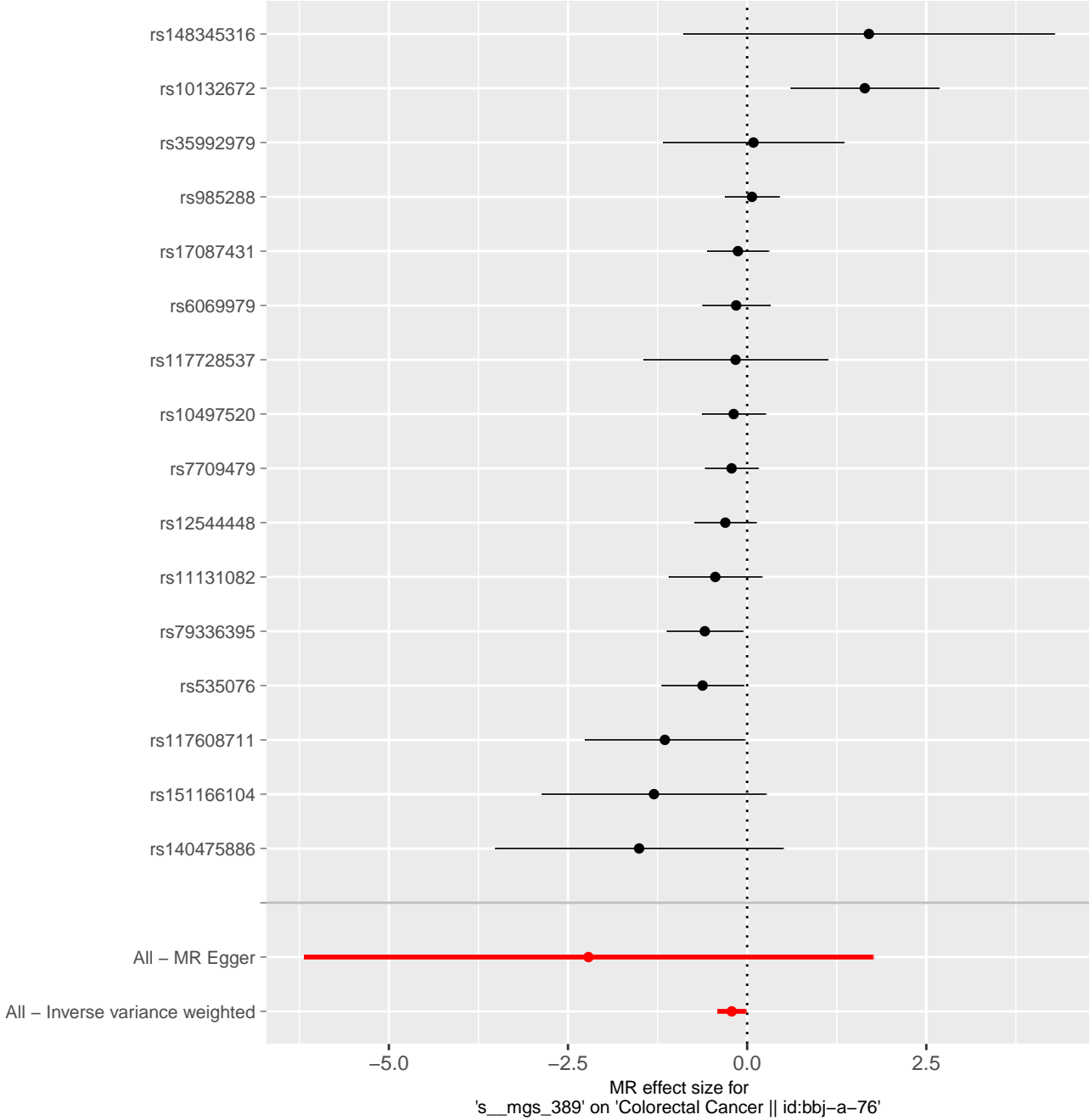

Supplement: Supplementary file 1 [file Supplementaryfile1.zip › Supplementary files 1 forest plot/saliva-pheno.2610.bbj-a-76.pdf]

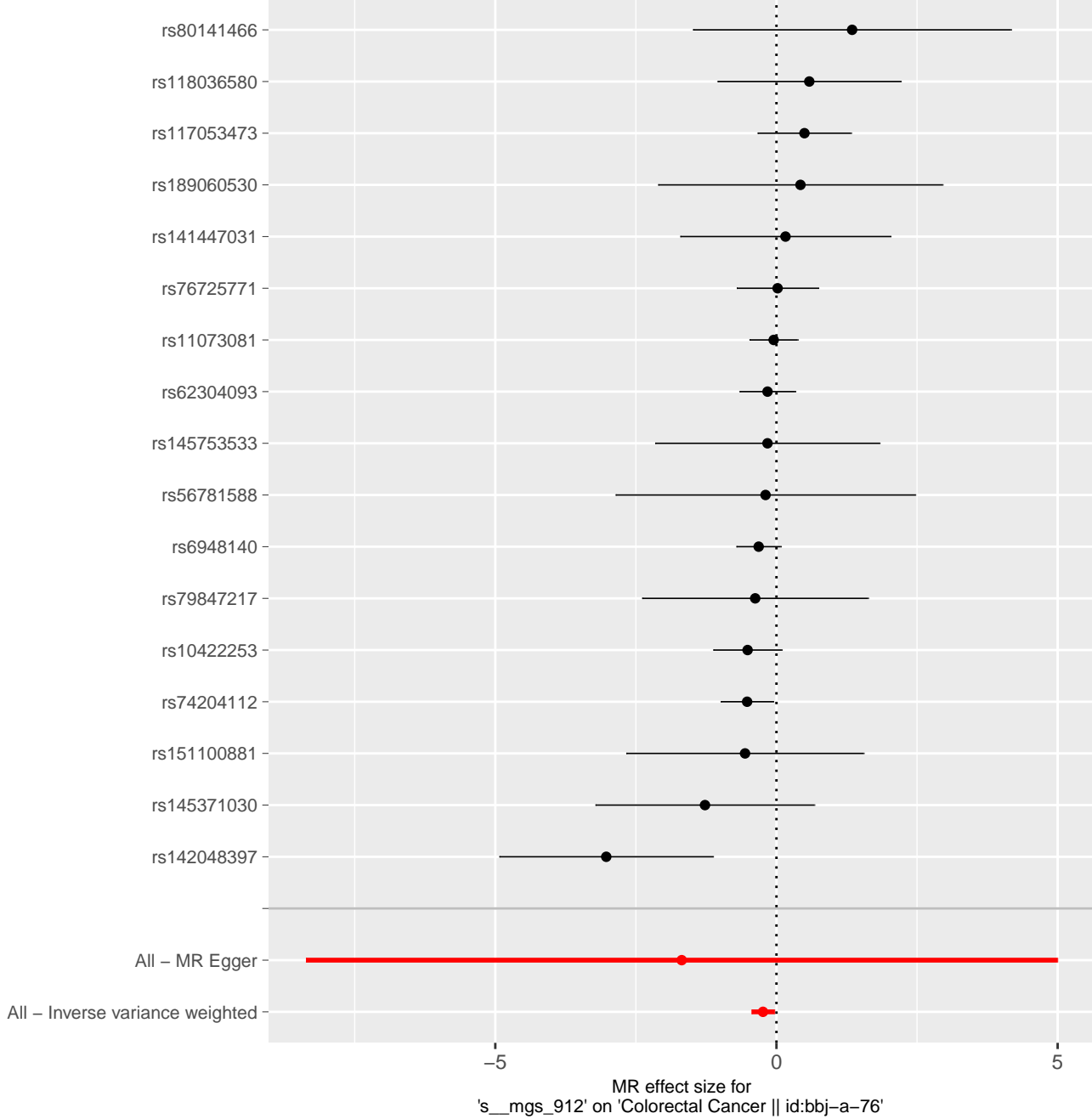

Supplement: Supplementary file 1 [file Supplementaryfile1.zip › Supplementary files 1 forest plot/saliva-pheno.3072.bbj-a-76.pdf]

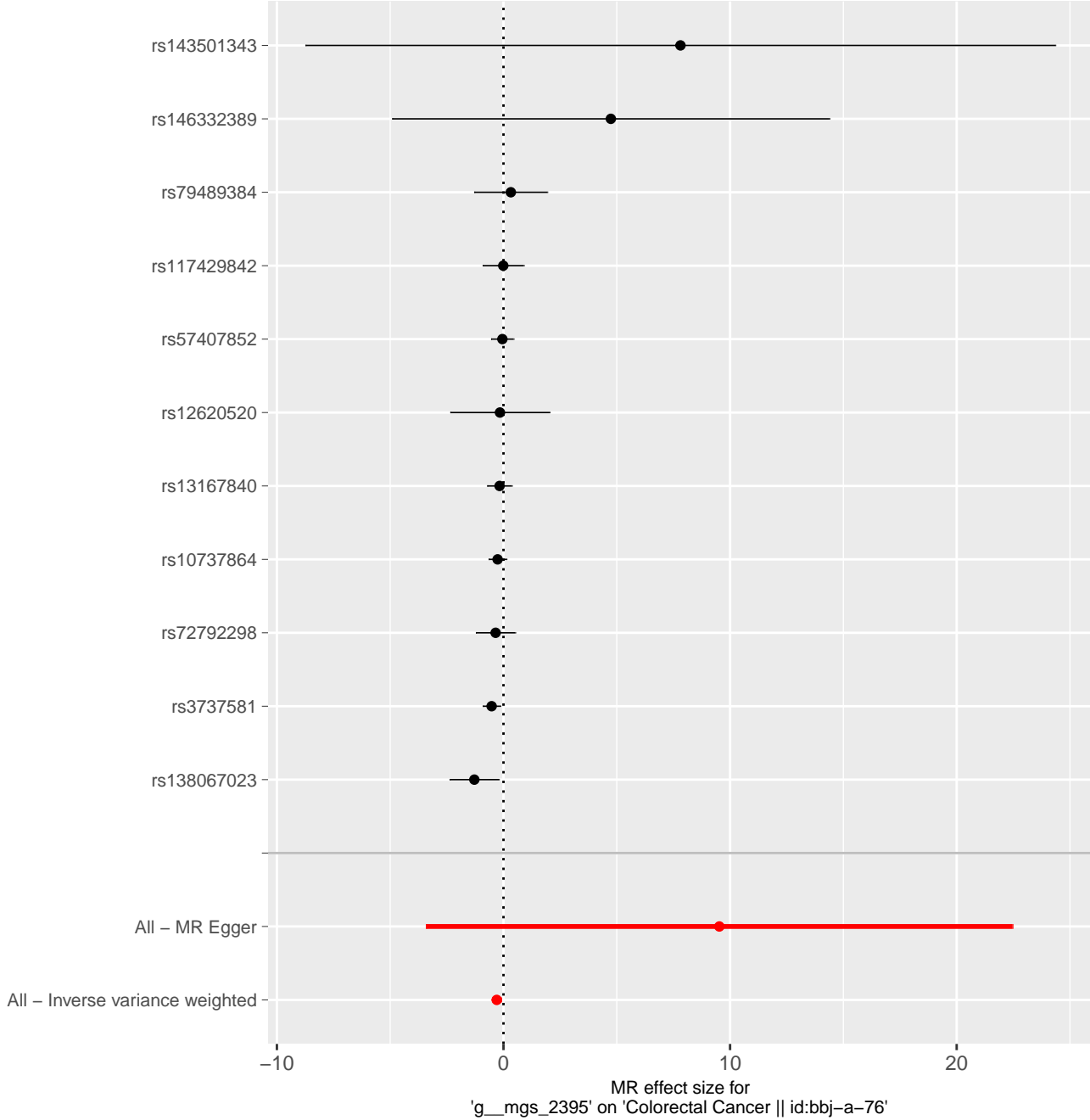

Supplement: Supplementary file 1 [file Supplementaryfile1.zip › Supplementary files 1 forest plot/saliva-pheno.654.bbj-a-76.pdf]

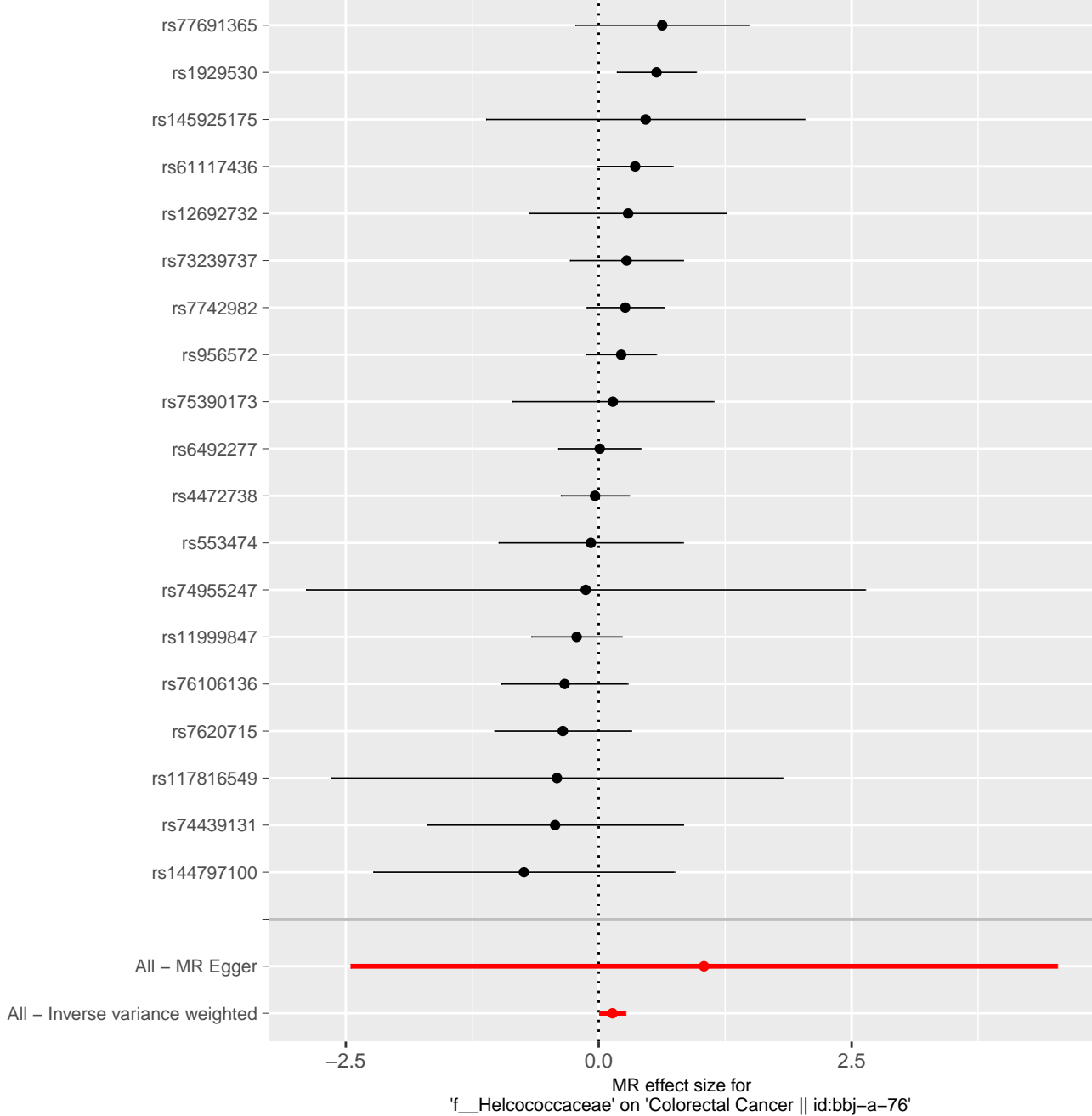

Supplement: Supplementary file 1 [file Supplementaryfile1.zip › Supplementary files 1 forest plot/saliva-pheno.673.bbj-a-76.pdf]

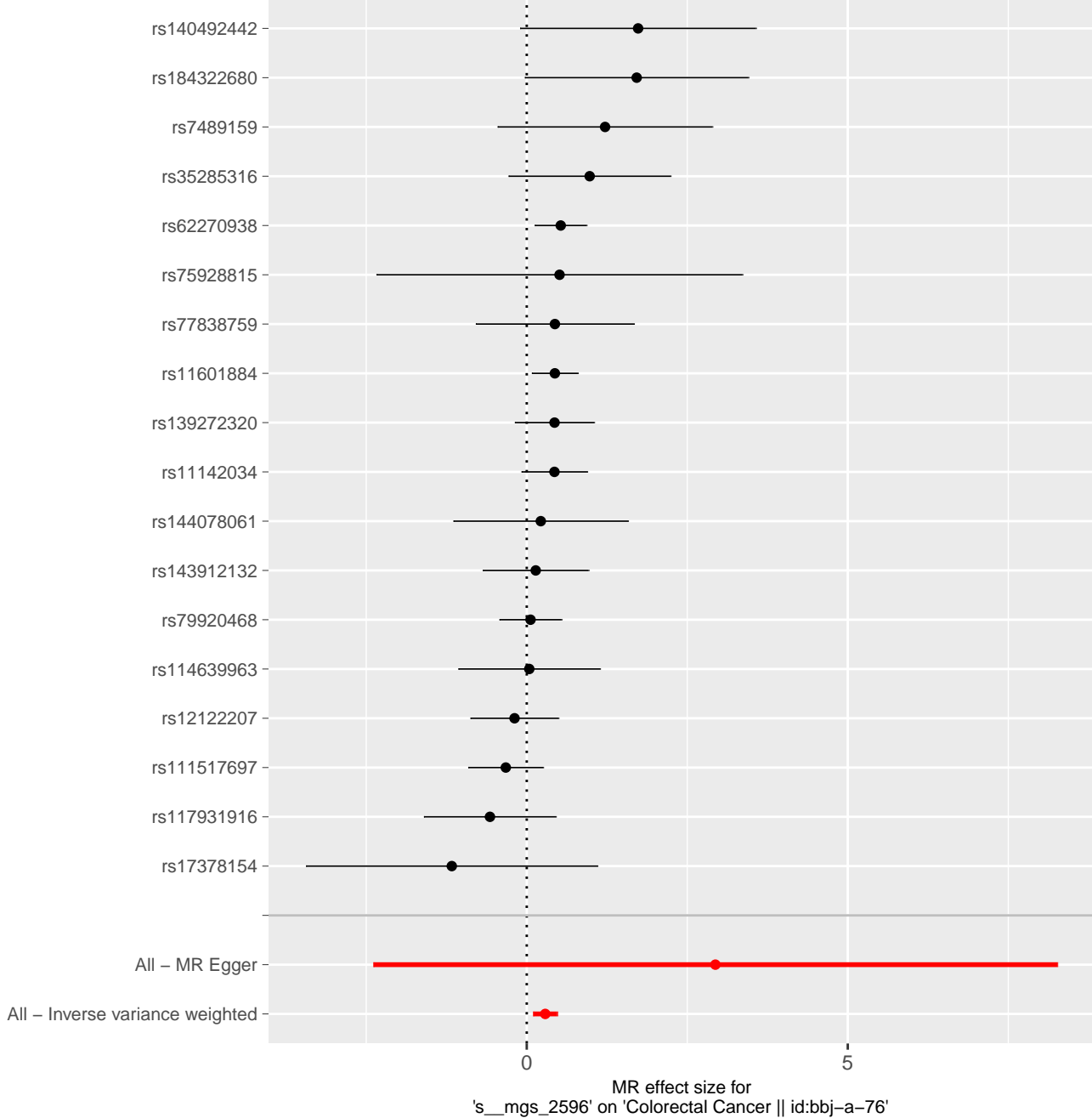

Supplement: Supplementary file 1 [file Supplementaryfile1.zip › Supplementary files 1 forest plot/saliva-pheno.725.bbj-a-76.pdf]

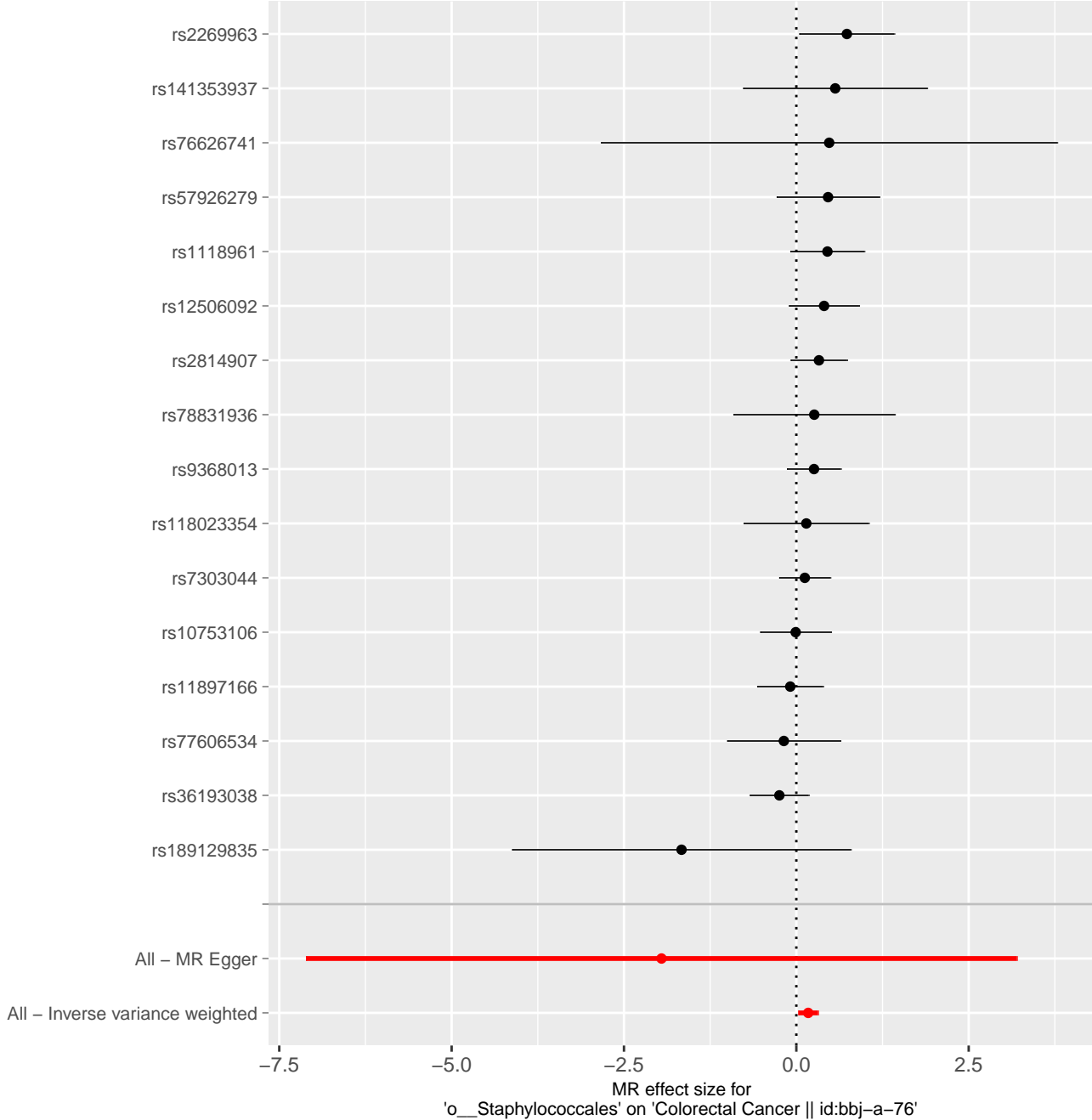

Supplement: Supplementary file 1 [file Supplementaryfile1.zip › Supplementary files 1 forest plot/saliva-pheno.846.bbj-a-76.pdf]

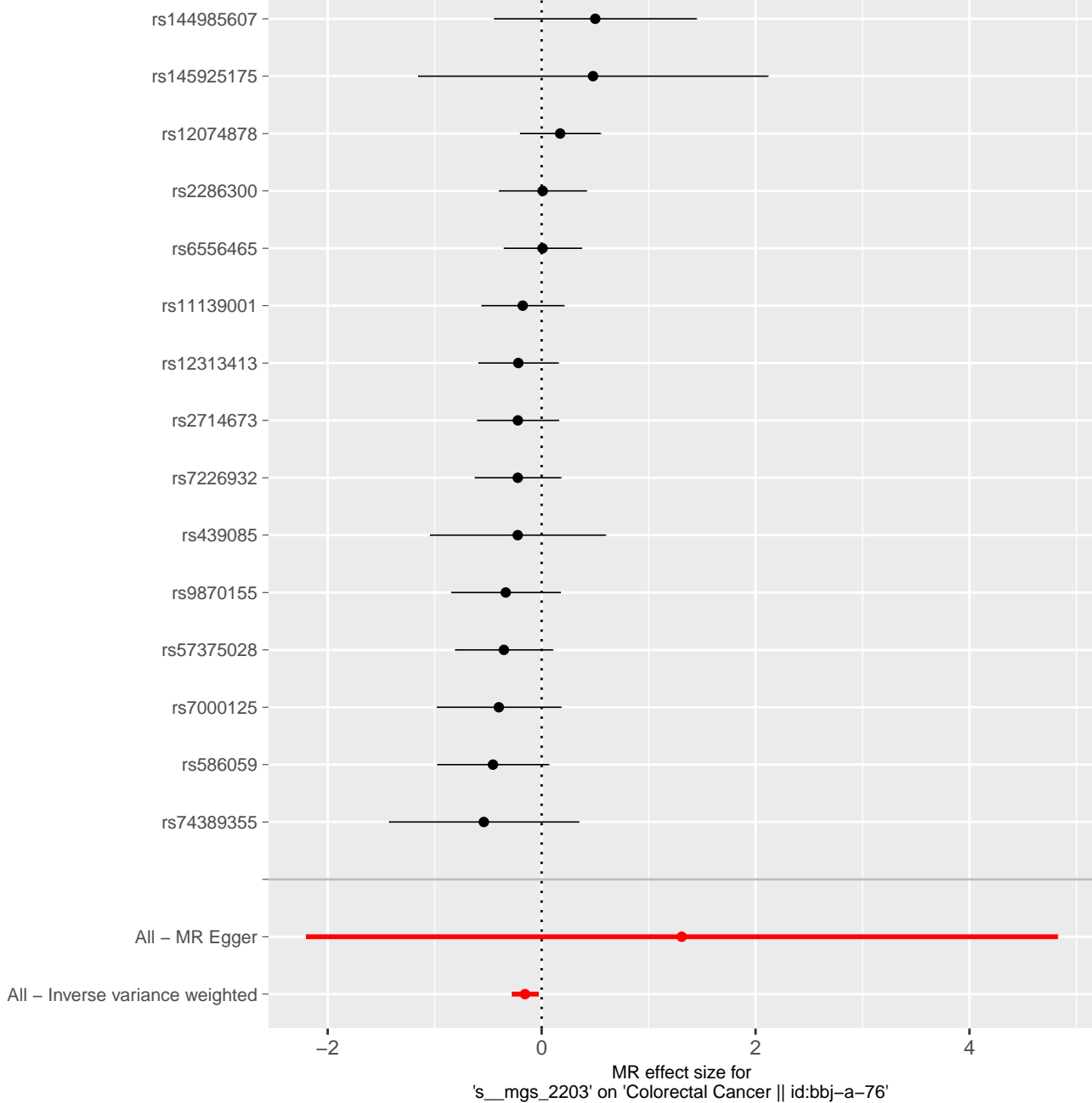

Supplement: Supplementary file 1 [file Supplementaryfile1.zip › Supplementary files 1 forest plot/saliva-pheno.87.bbj-a-76.pdf]

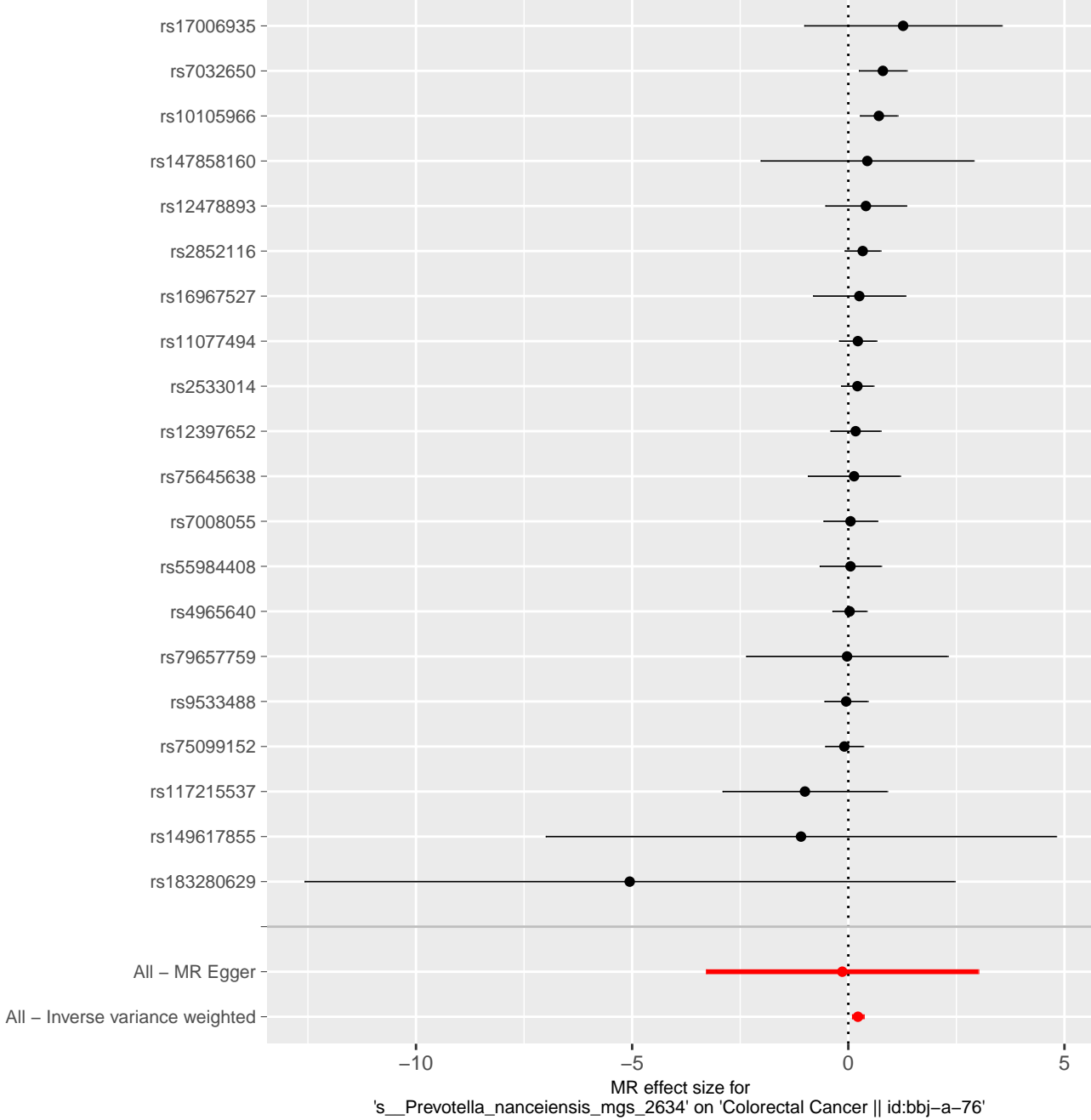

Supplement: Supplementary file 1 [file Supplementaryfile1.zip › Supplementary files 1 forest plot/tongue-pheno.1229.bbj-a-76.pdf]

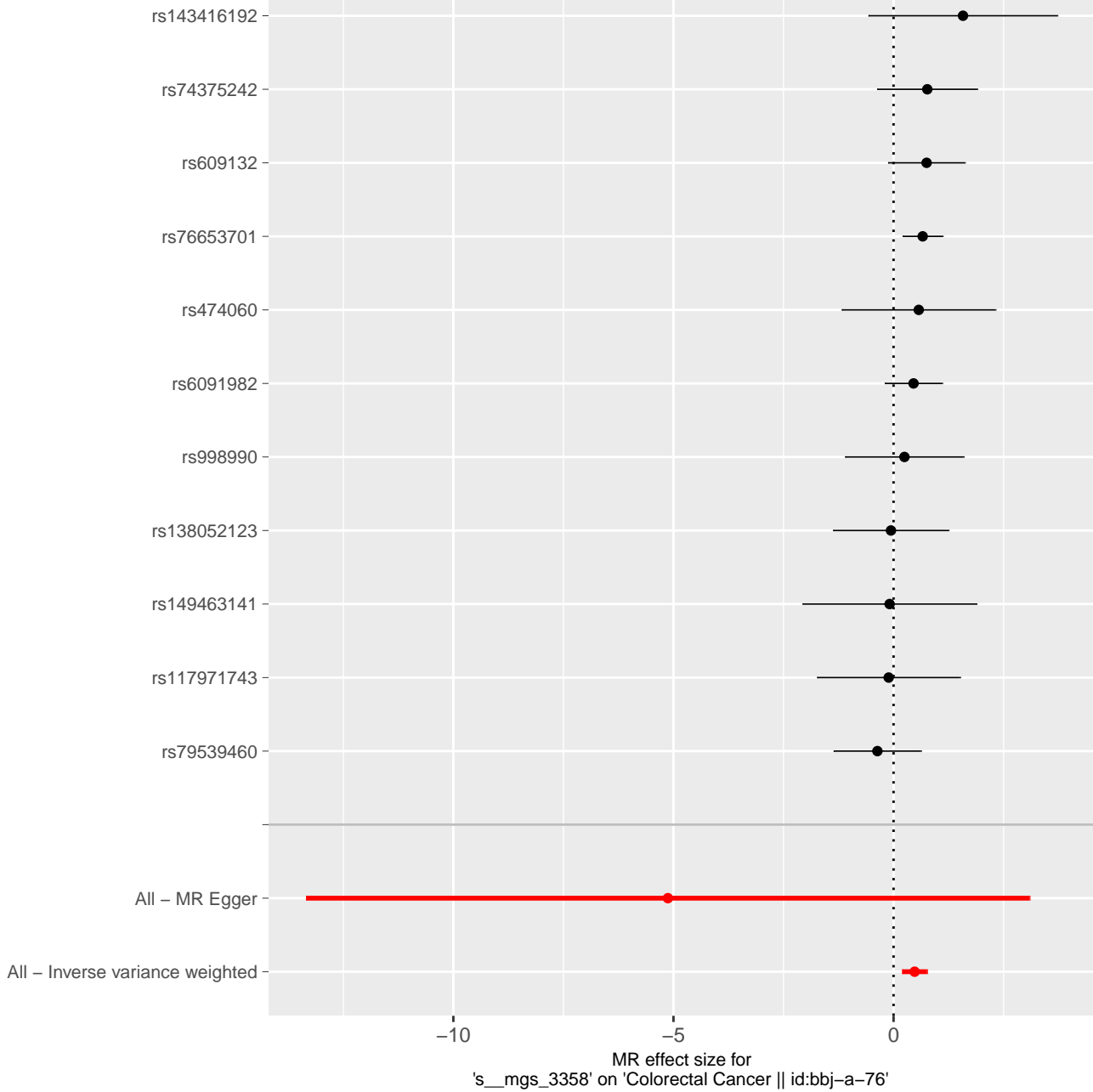

Supplement: Supplementary file 1 [file Supplementaryfile1.zip › Supplementary files 1 forest plot/tongue-pheno.145.bbj-a-76.pdf]

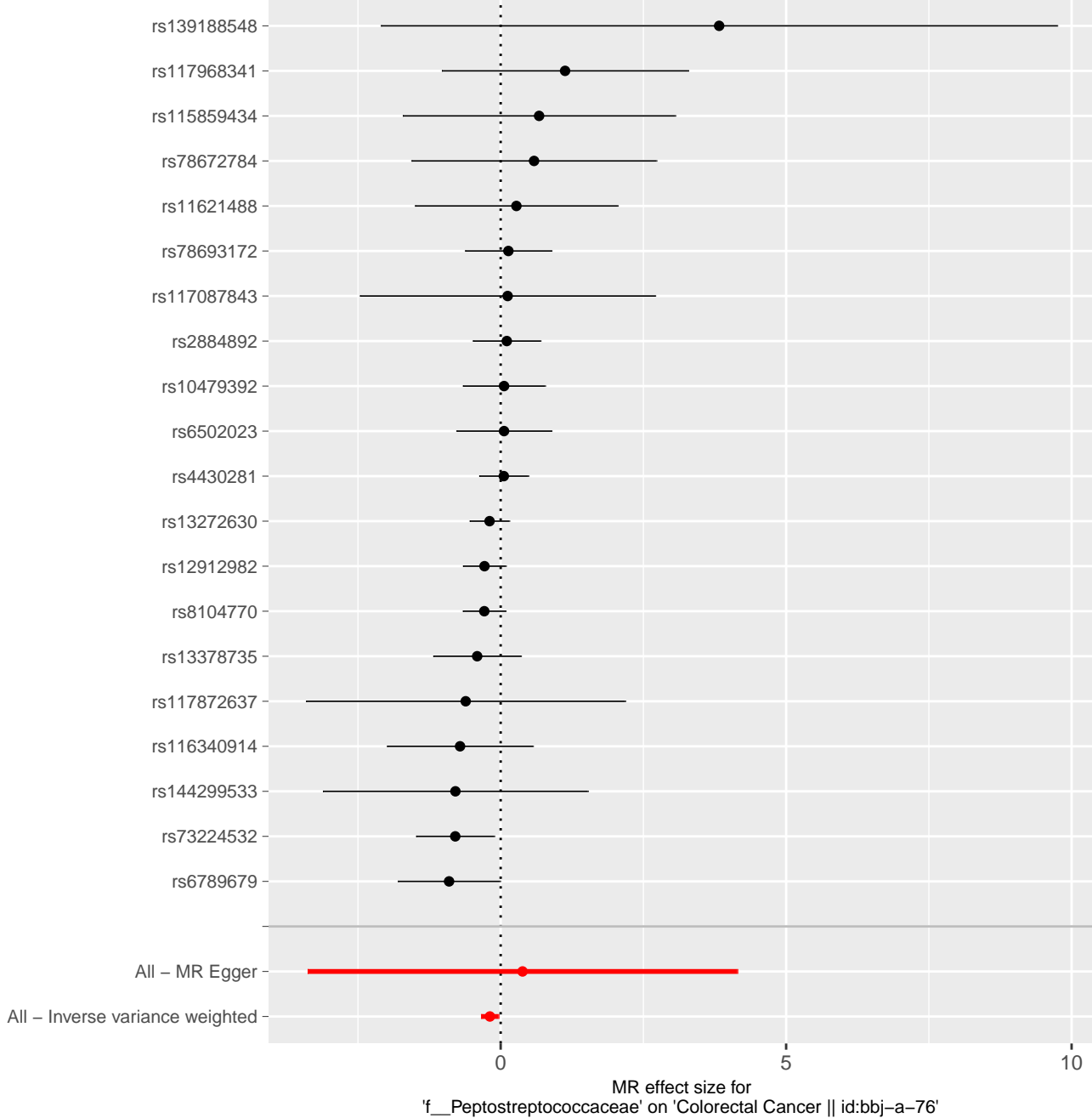

Supplement: Supplementary file 1 [file Supplementaryfile1.zip › Supplementary files 1 forest plot/tongue-pheno.1637.bbj-a-76.pdf]

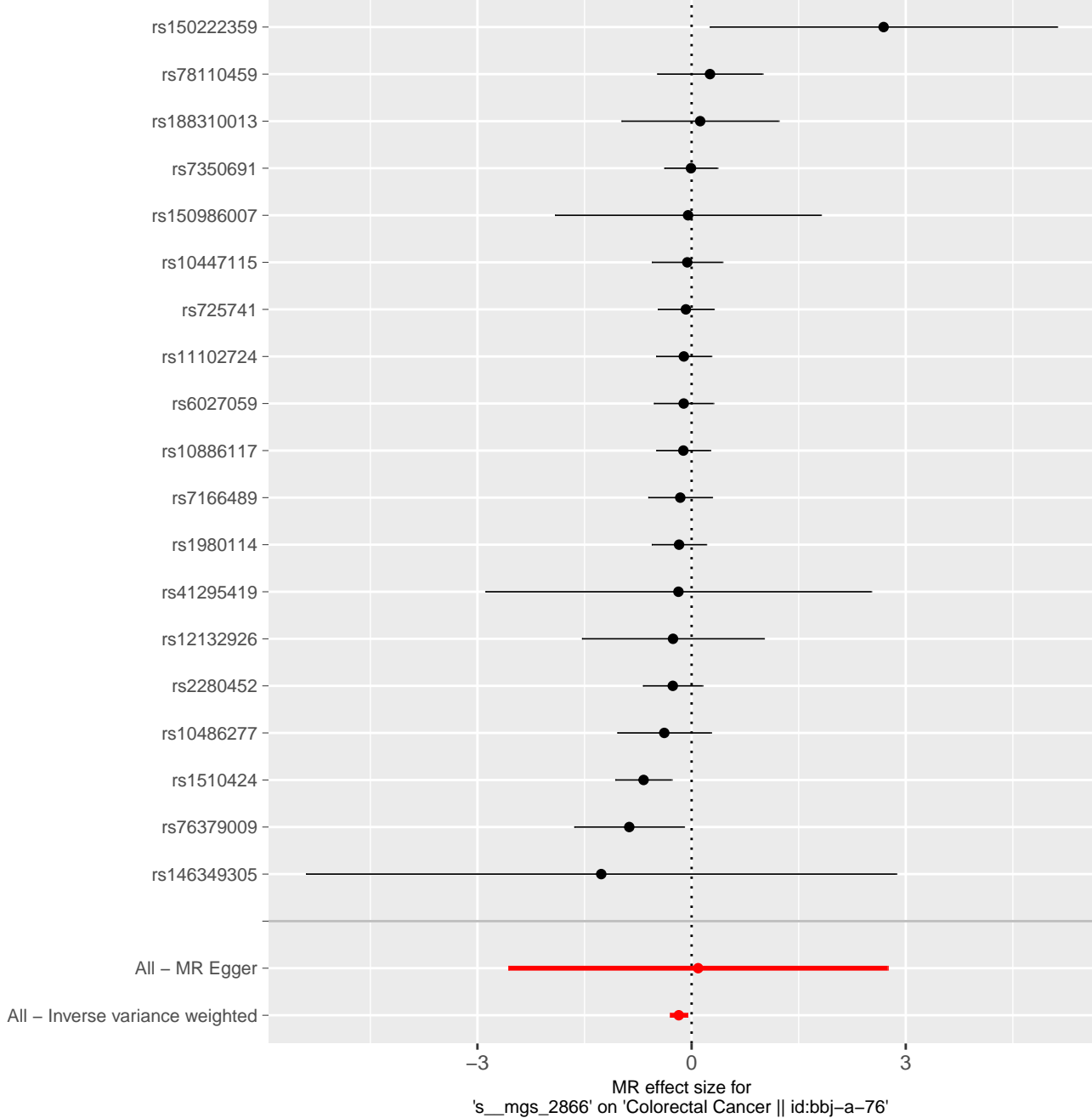

Supplement: Supplementary file 1 [file Supplementaryfile1.zip › Supplementary files 1 forest plot/tongue-pheno.1653.bbj-a-76.pdf]

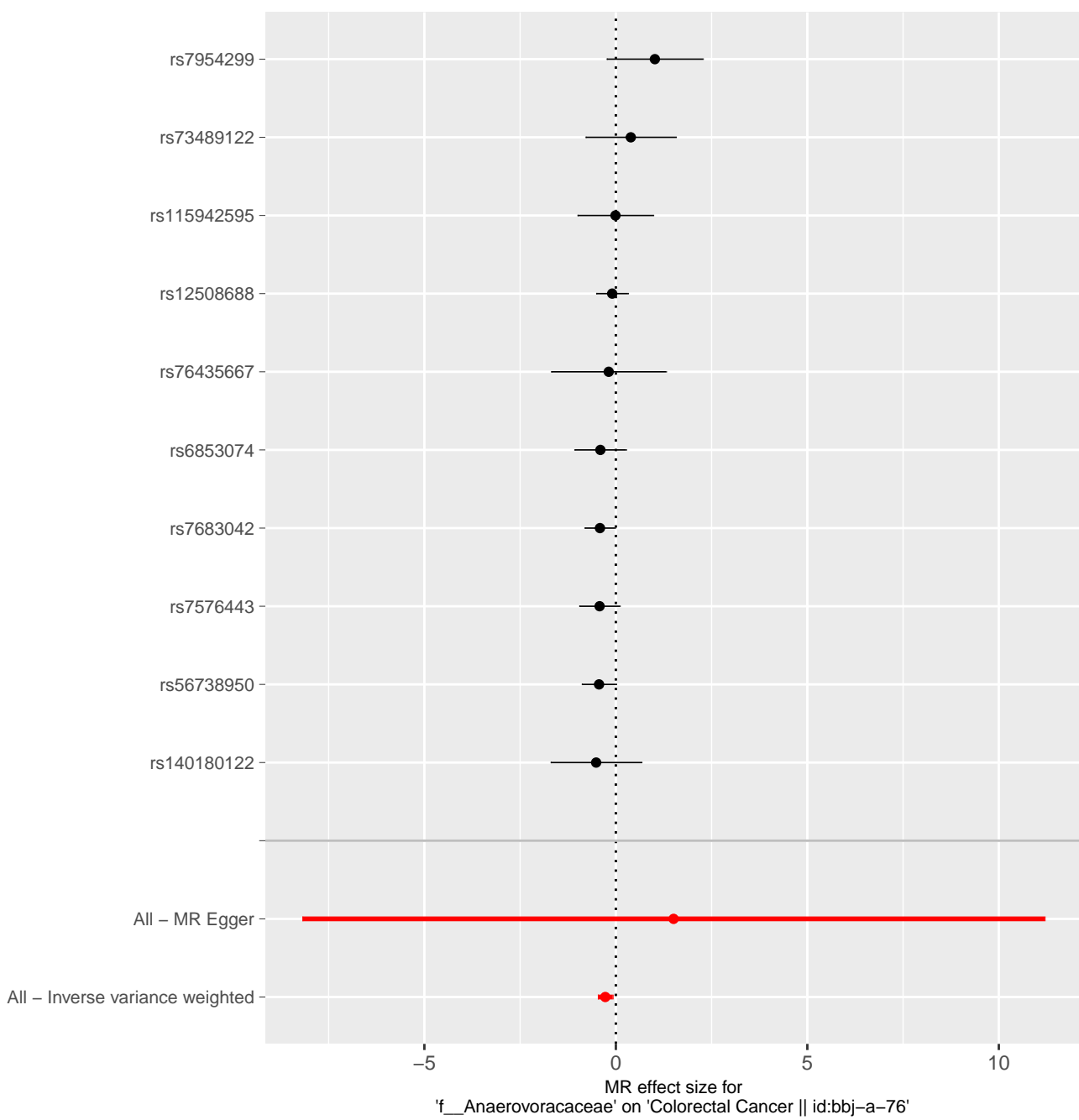

Supplement: Supplementary file 1 [file Supplementaryfile1.zip › Supplementary files 1 forest plot/tongue-pheno.1781.bbj-a-76.pdf]

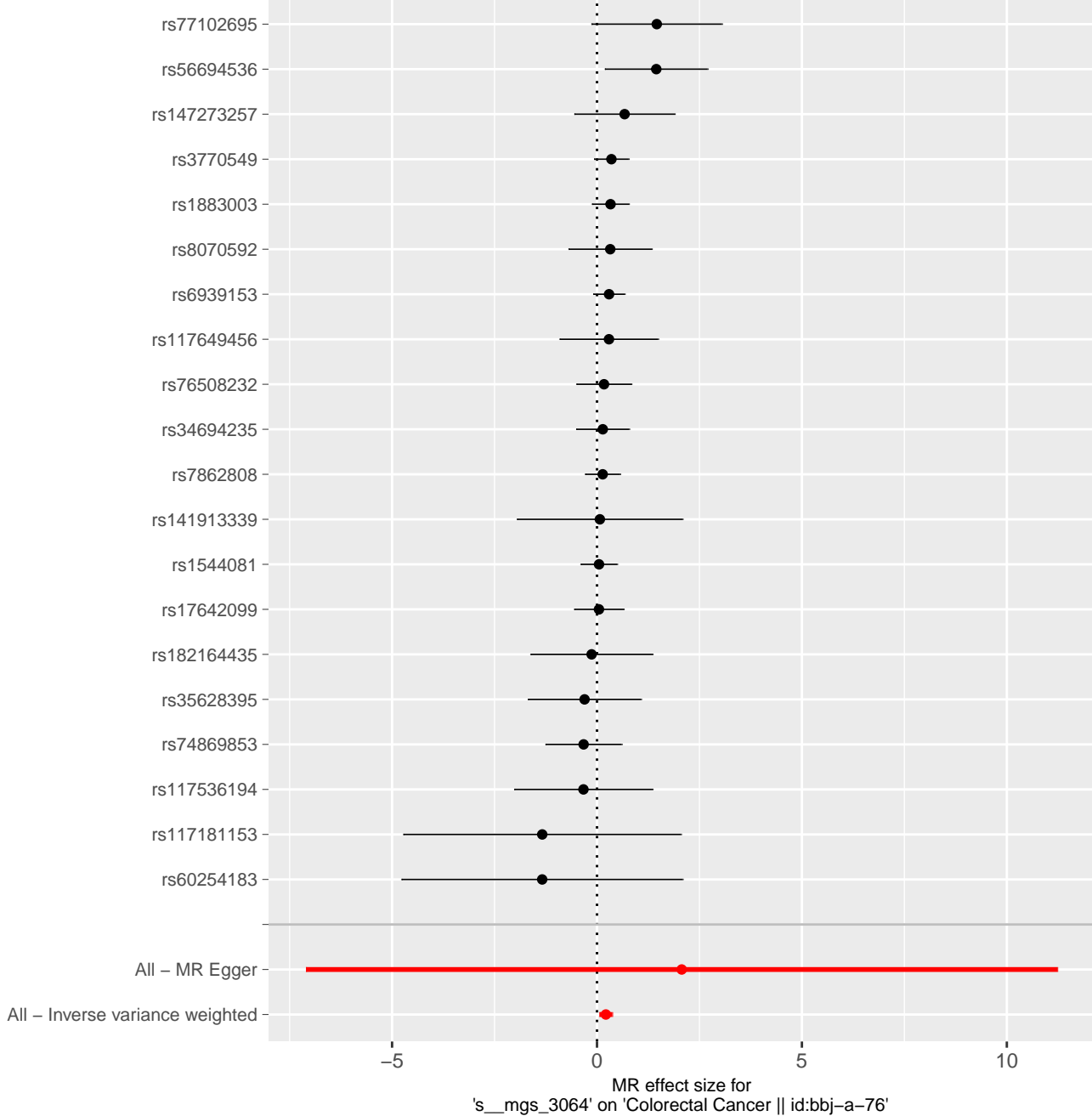

Supplement: Supplementary file 1 [file Supplementaryfile1.zip › Supplementary files 1 forest plot/tongue-pheno.3410.bbj-a-76.pdf]

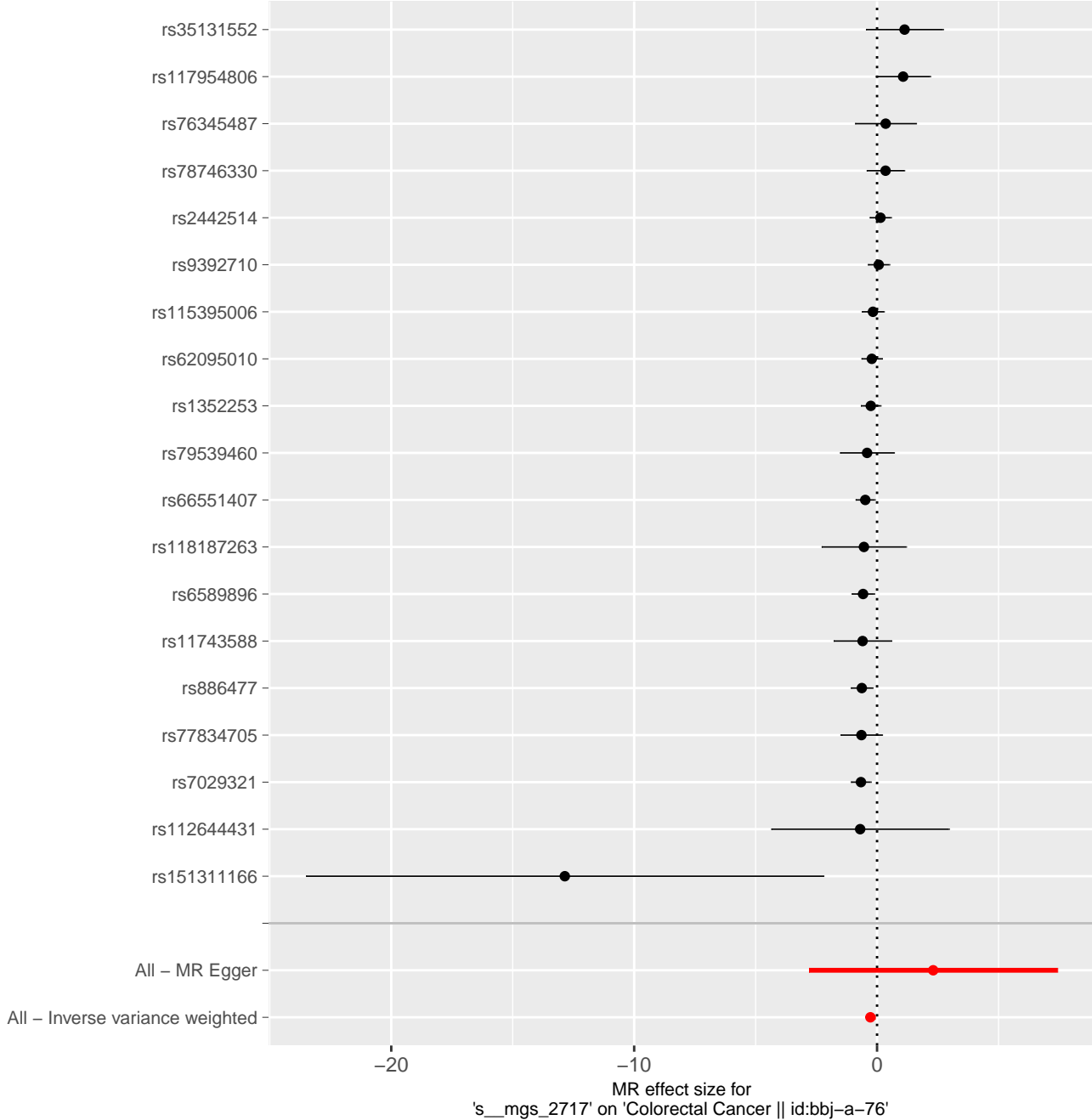

Supplement: Supplementary file 1 [file Supplementaryfile1.zip › Supplementary files 1 forest plot/tongue-pheno.368.bbj-a-76.pdf]

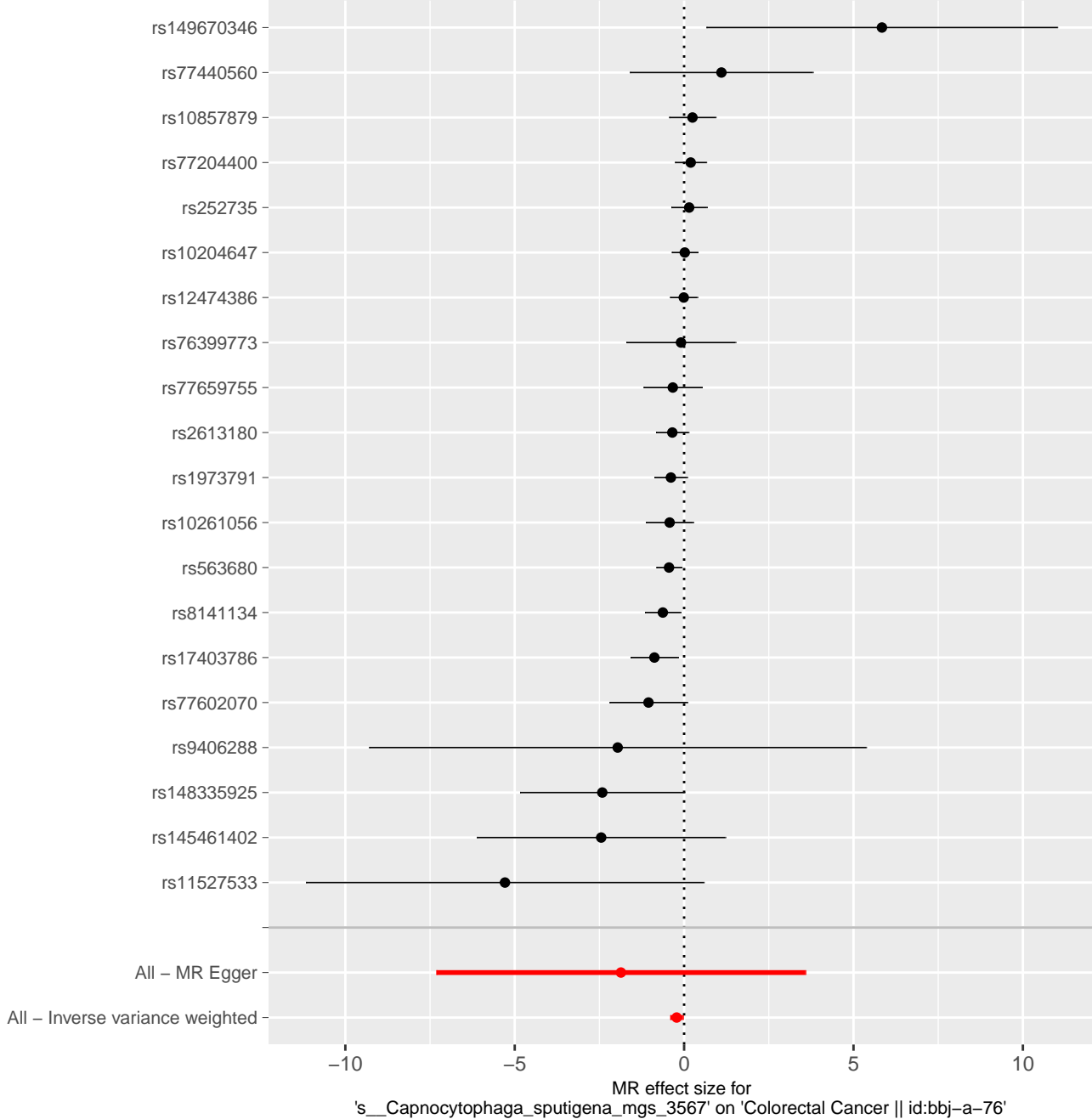

Supplement: Supplementary file 1 [file Supplementaryfile1.zip › Supplementary files 1 forest plot/tongue-pheno.768.bbj-a-76.pdf]

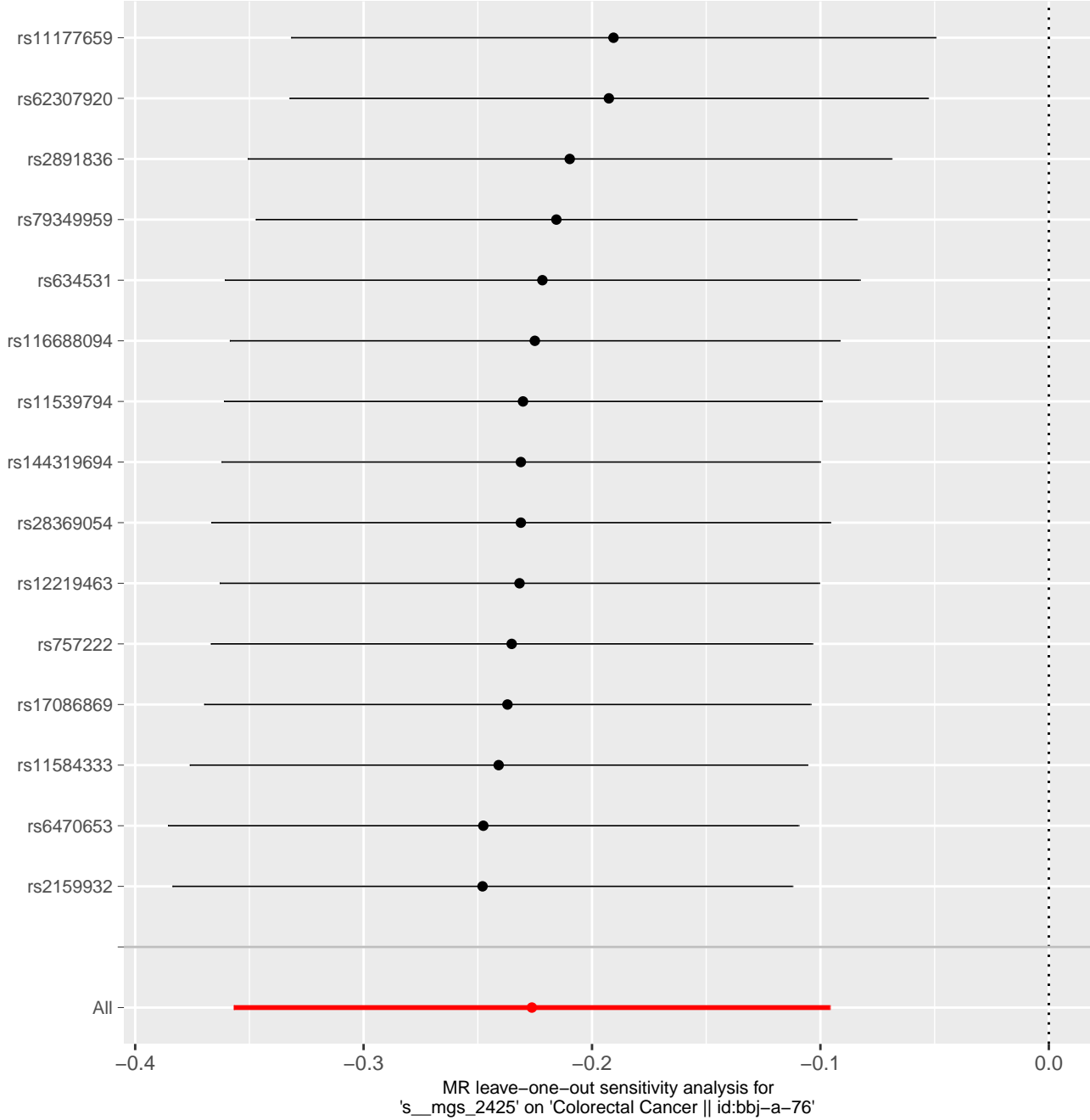

Supplement: Supplementary file 2 [file Supplementaryfile2.zip › Supplementary files 2 leave-one-out plot/saliva-pheno.1269.bbj-a-76.pdf]

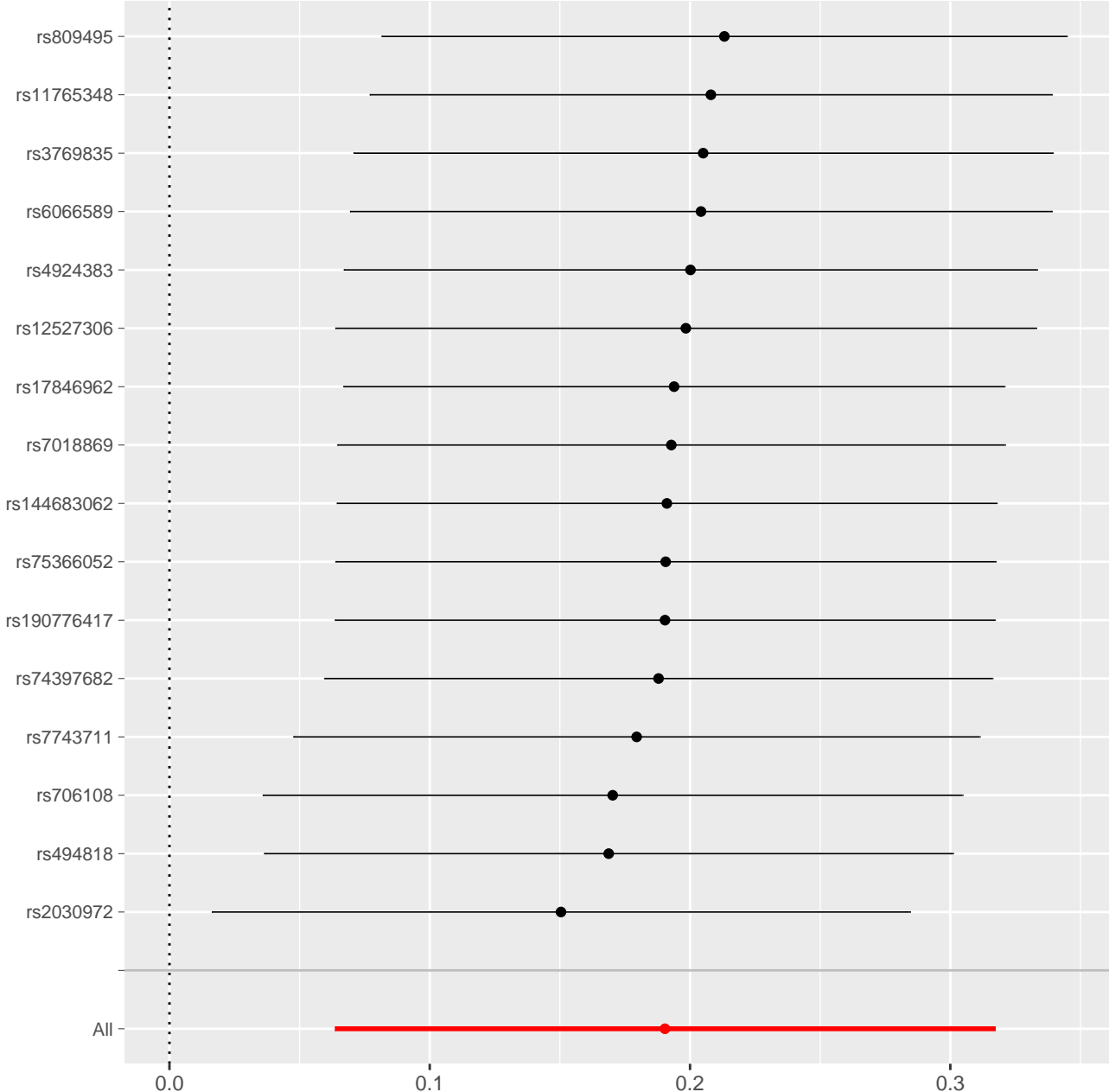

Supplement: Supplementary file 2 [file Supplementaryfile2.zip › Supplementary files 2 leave-one-out plot/saliva-pheno.1542.bbj-a-76.pdf]

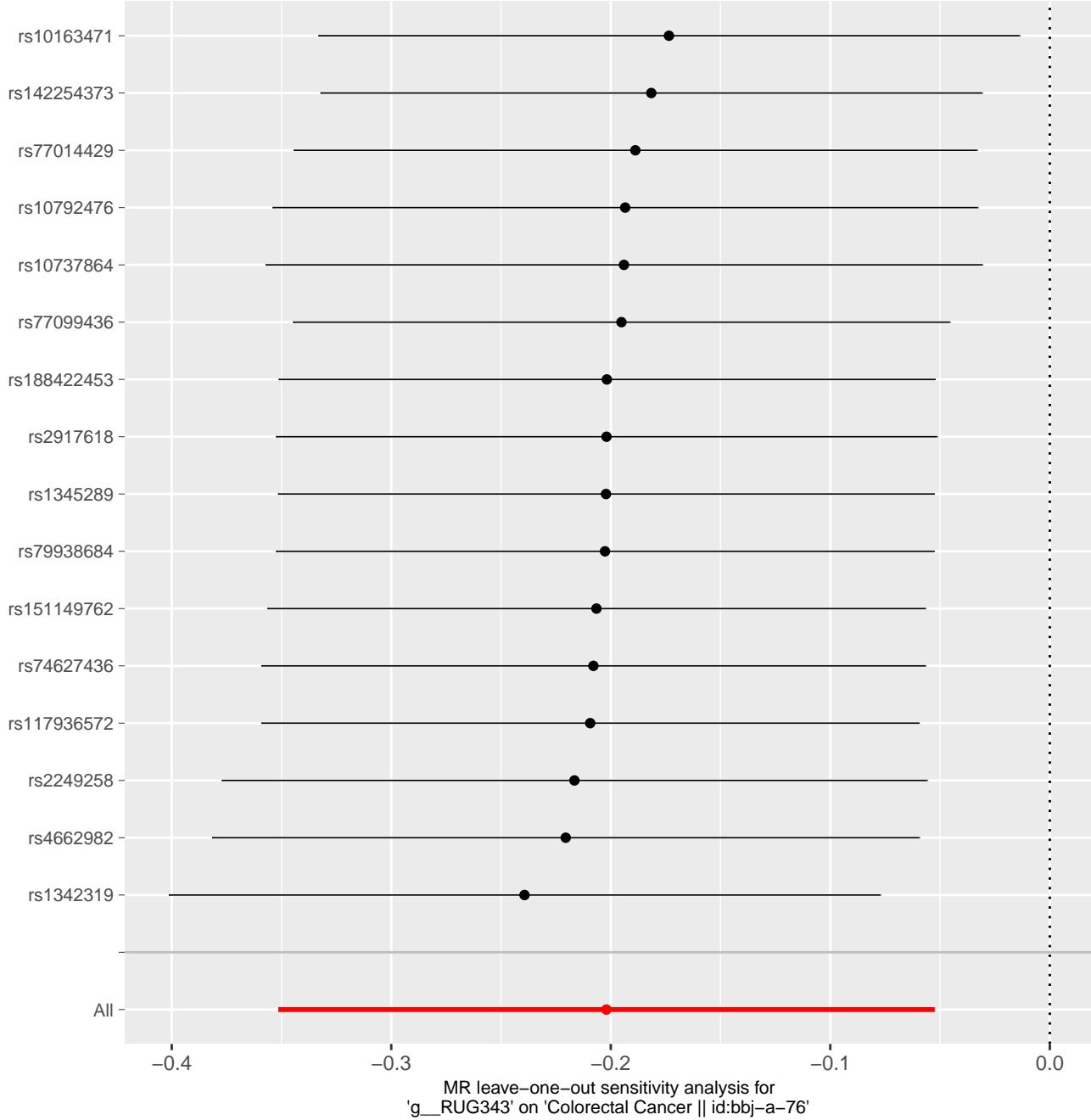

Supplement: Supplementary file 2 [file Supplementaryfile2.zip › Supplementary files 2 leave-one-out plot/saliva-pheno.160.bbj-a-76.pdf]

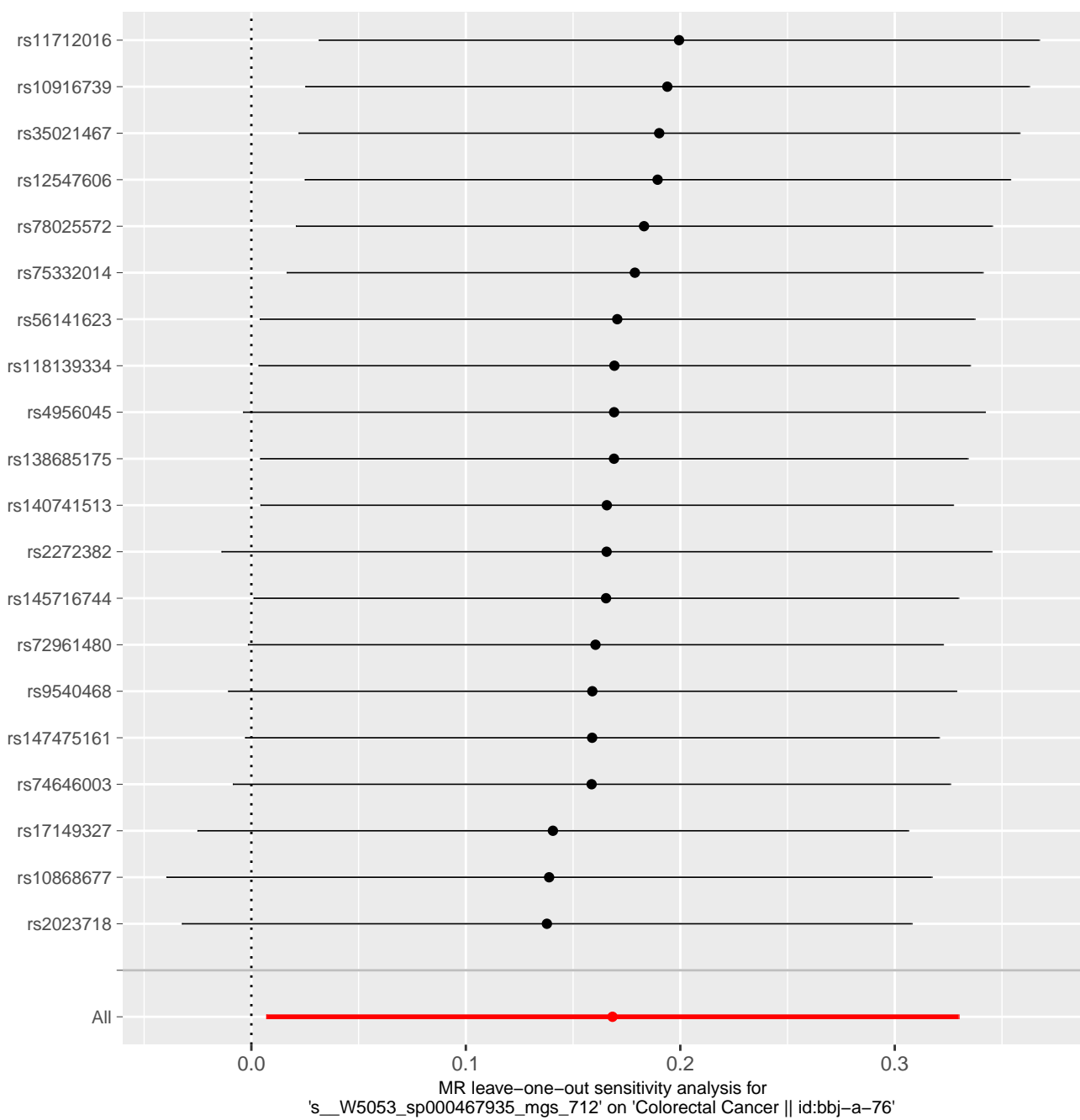

Supplement: Supplementary file 2 [file Supplementaryfile2.zip › Supplementary files 2 leave-one-out plot/saliva-pheno.2303.bbj-a-76.pdf]

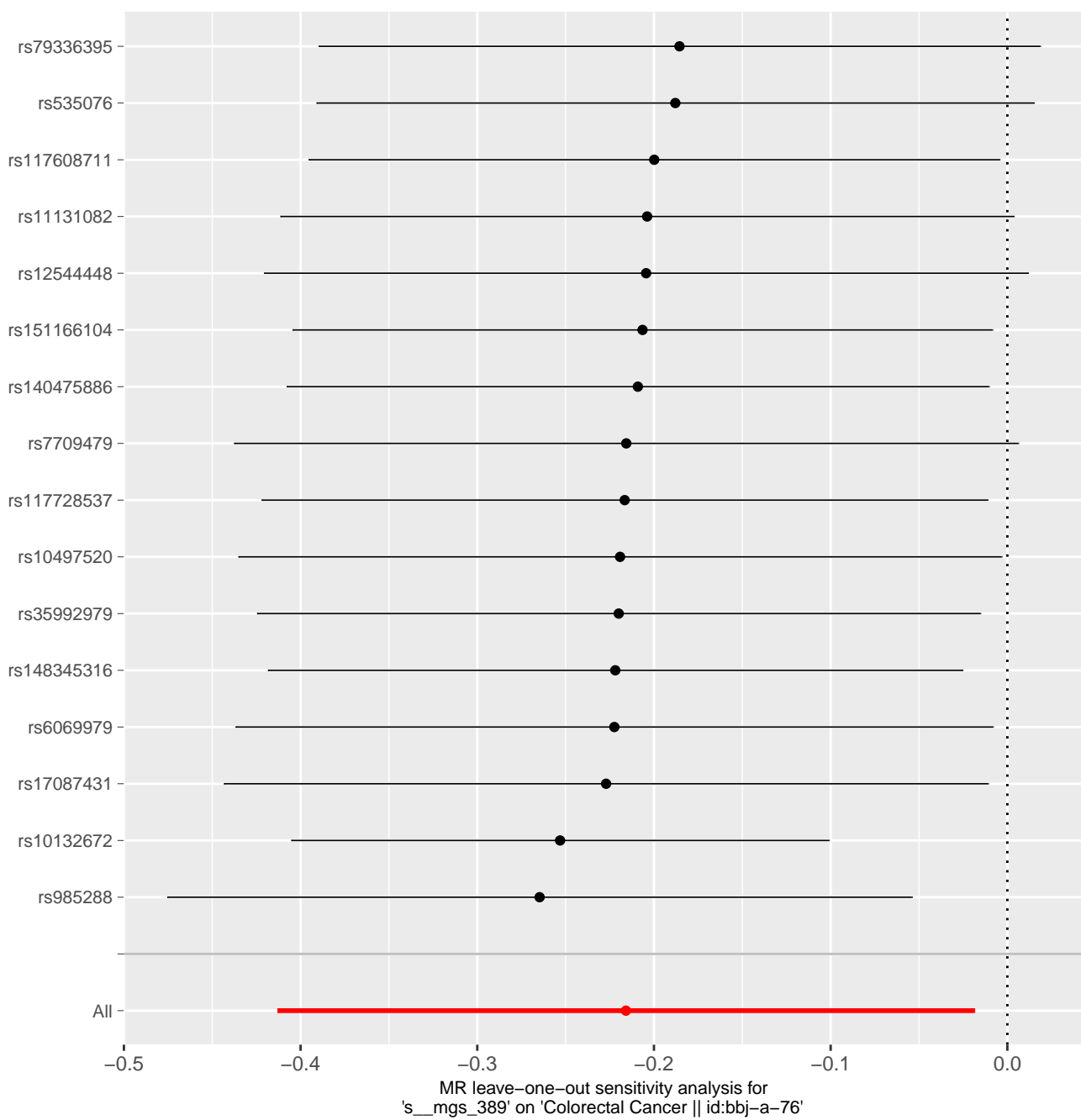

Supplement: Supplementary file 2 [file Supplementaryfile2.zip › Supplementary files 2 leave-one-out plot/saliva-pheno.2610.bbj-a-76.pdf]

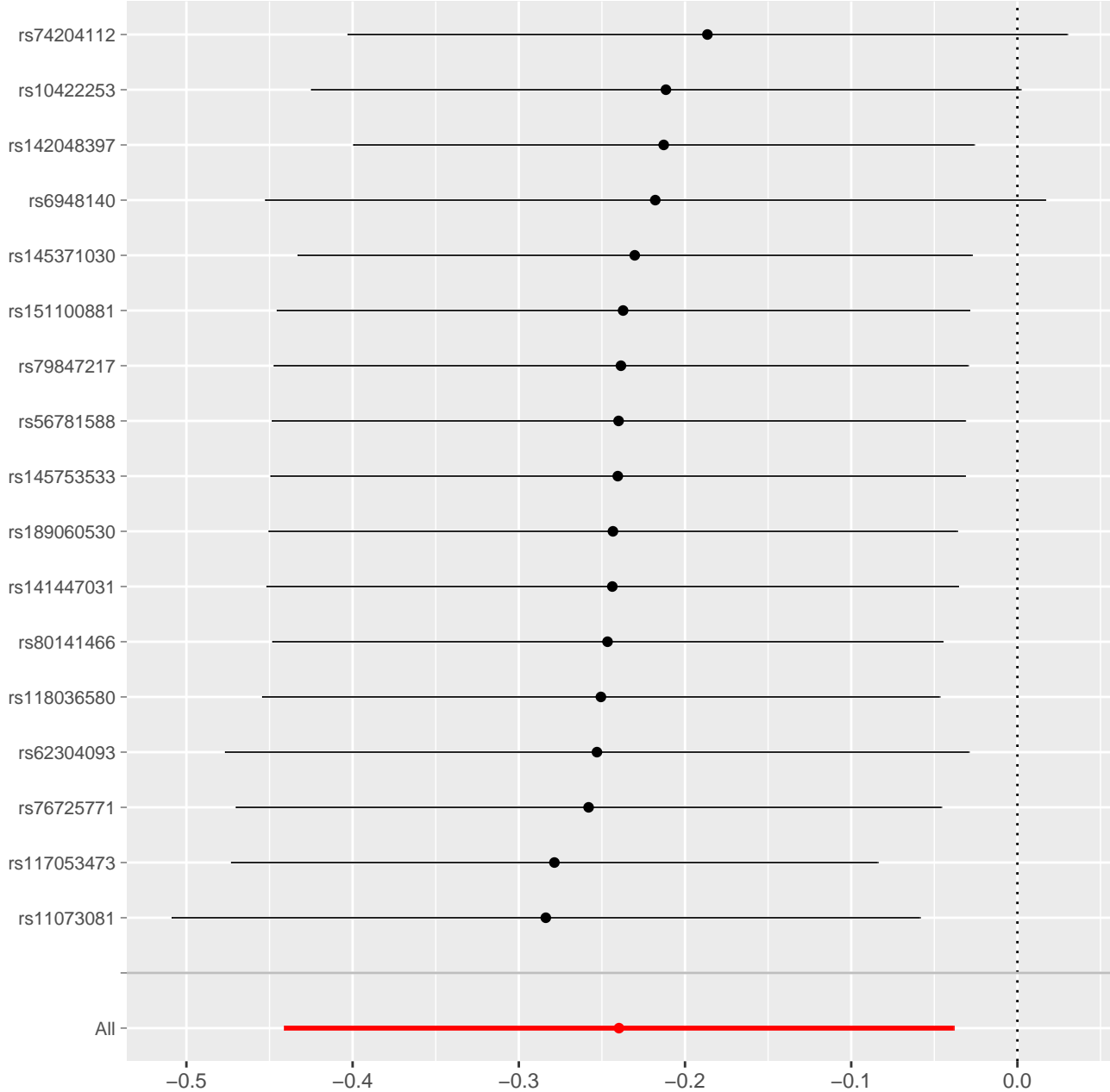

MR leave-one-out sensitivity analysis for  
's\_mgs\_912' on 'Colorectal Cancer || id:bbj-a-76'

Supplement: Supplementary file 2 [file Supplementaryfile2.zip › Supplementary files 2 leave-one-out plot/saliva-pheno.3072.bbj-a-76.pdf]

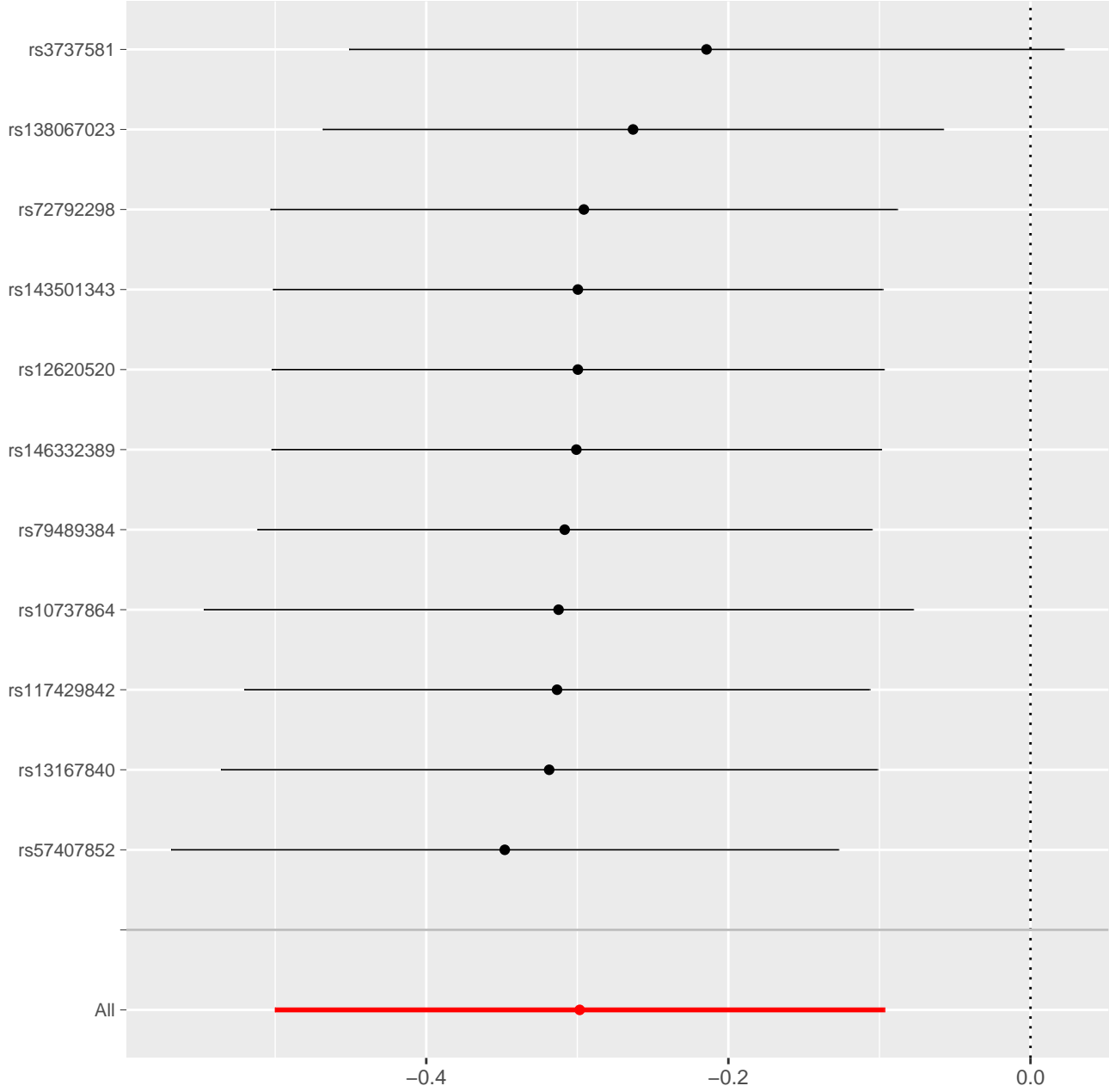

Supplement: Supplementary file 2 [file Supplementaryfile2.zip › Supplementary files 2 leave-one-out plot/saliva-pheno.654.bbj-a-76.pdf]

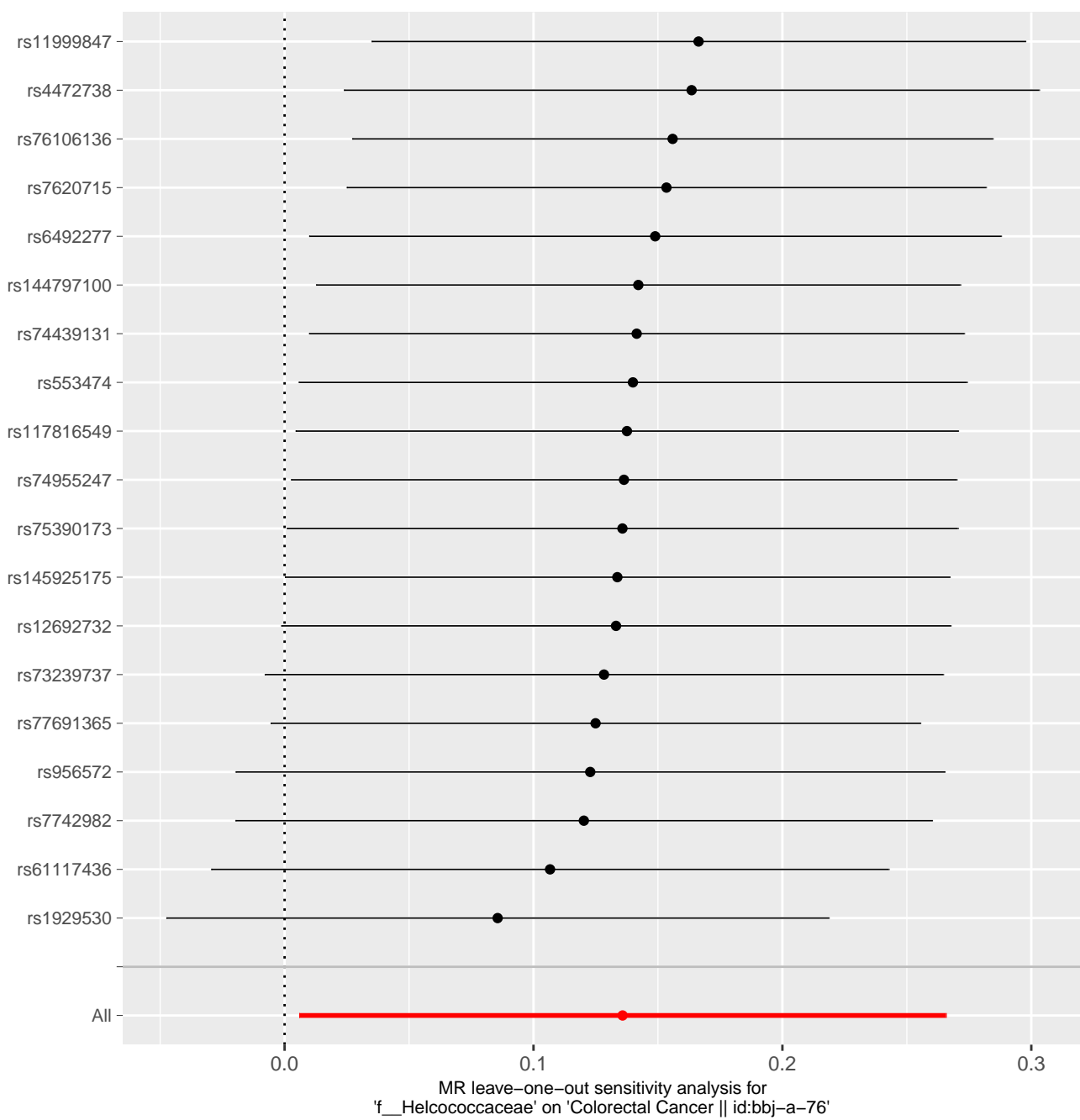

Supplement: Supplementary file 2 [file Supplementaryfile2.zip › Supplementary files 2 leave-one-out plot/saliva-pheno.673.bbj-a-76.pdf]

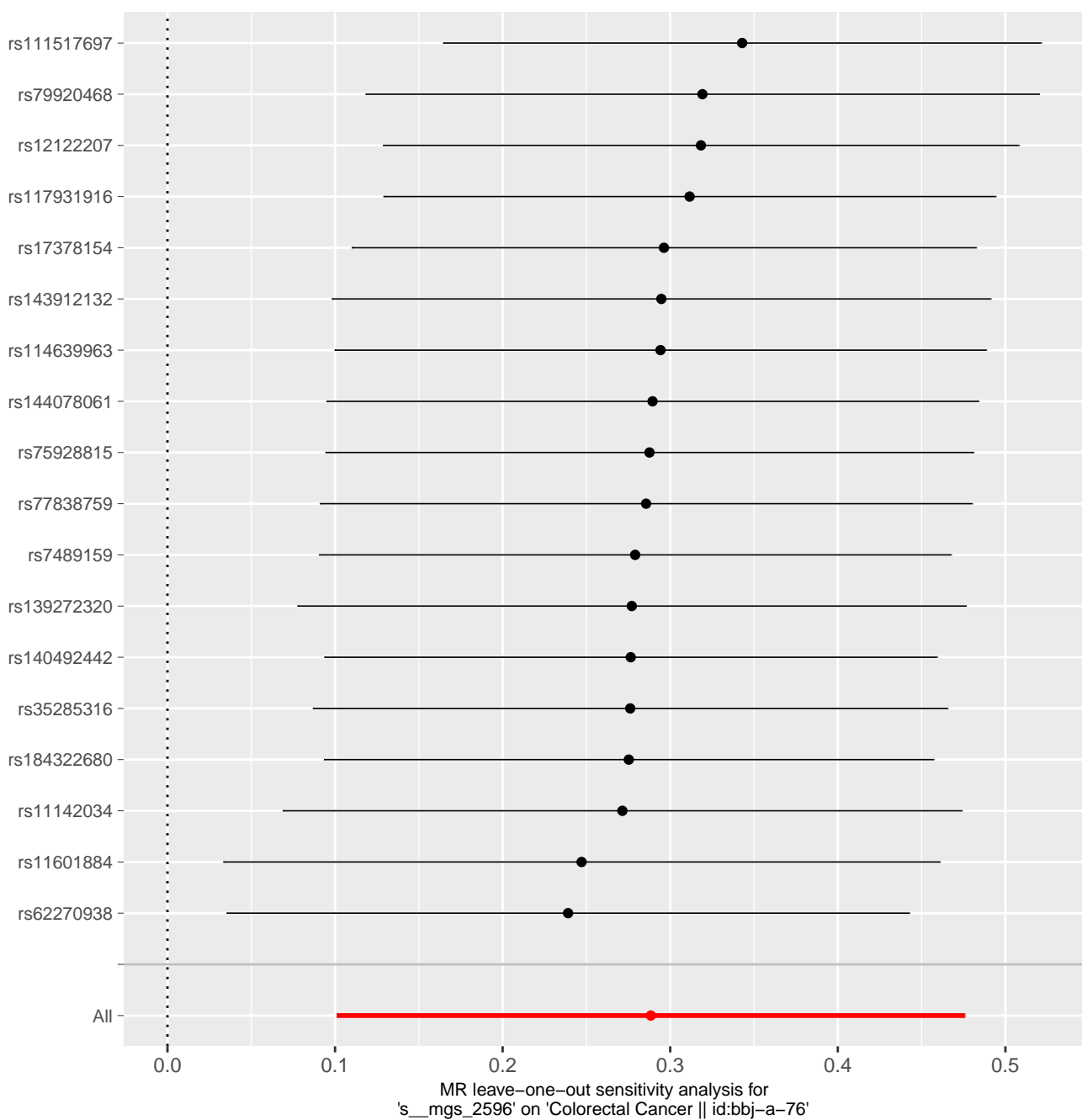

Supplement: Supplementary file 2 [file Supplementaryfile2.zip › Supplementary files 2 leave-one-out plot/saliva-pheno.725.bbj-a-76.pdf]

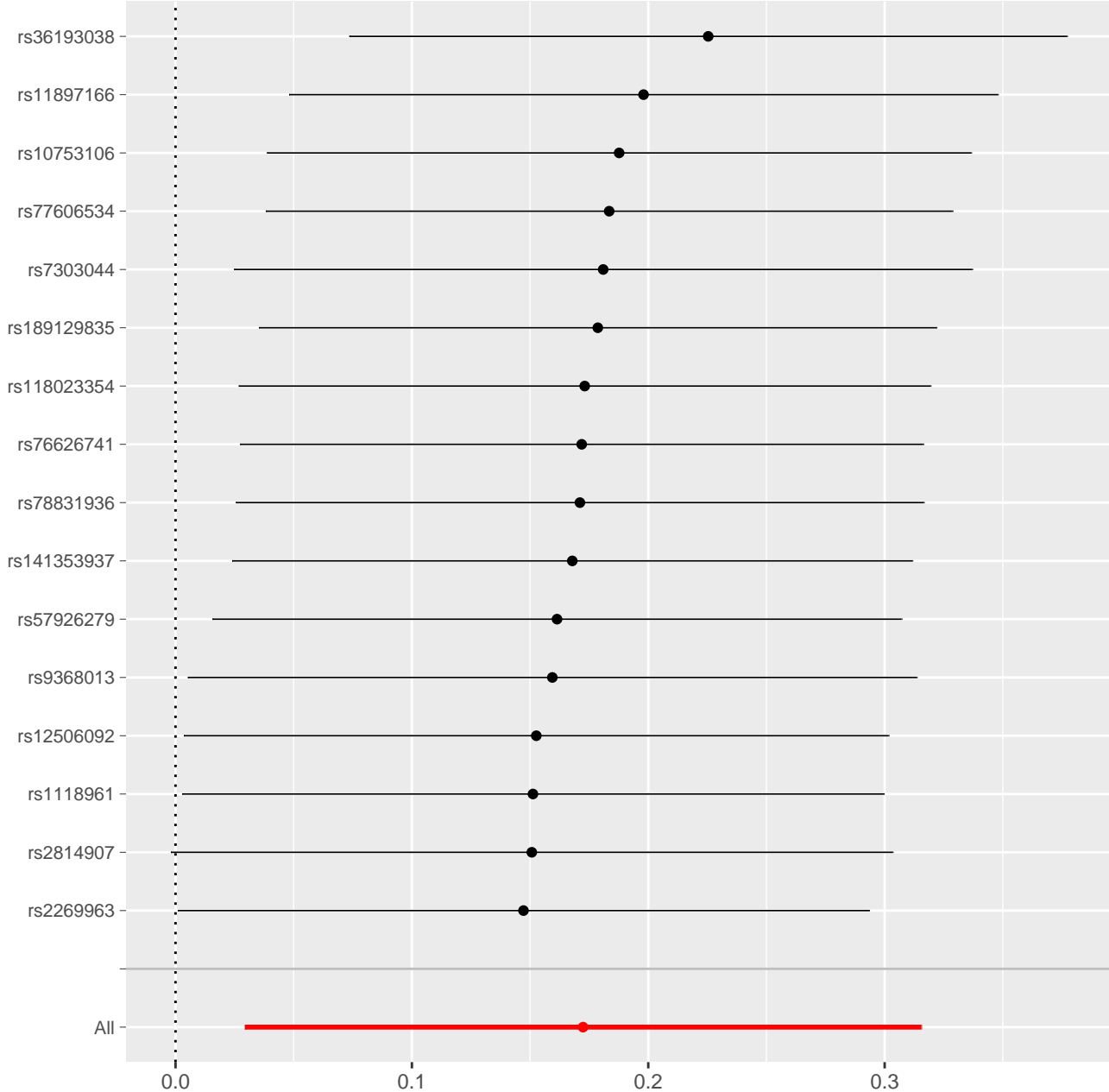

MR leave-one-out sensitivity analysis for  
'o\_Staphylococcales' on 'Colorectal Cancer || id:bbj-a-76'

Supplement: Supplementary file 2 [file Supplementaryfile2.zip › Supplementary files 2 leave-one-out plot/saliva-pheno.846.bbj-a-76.pdf]

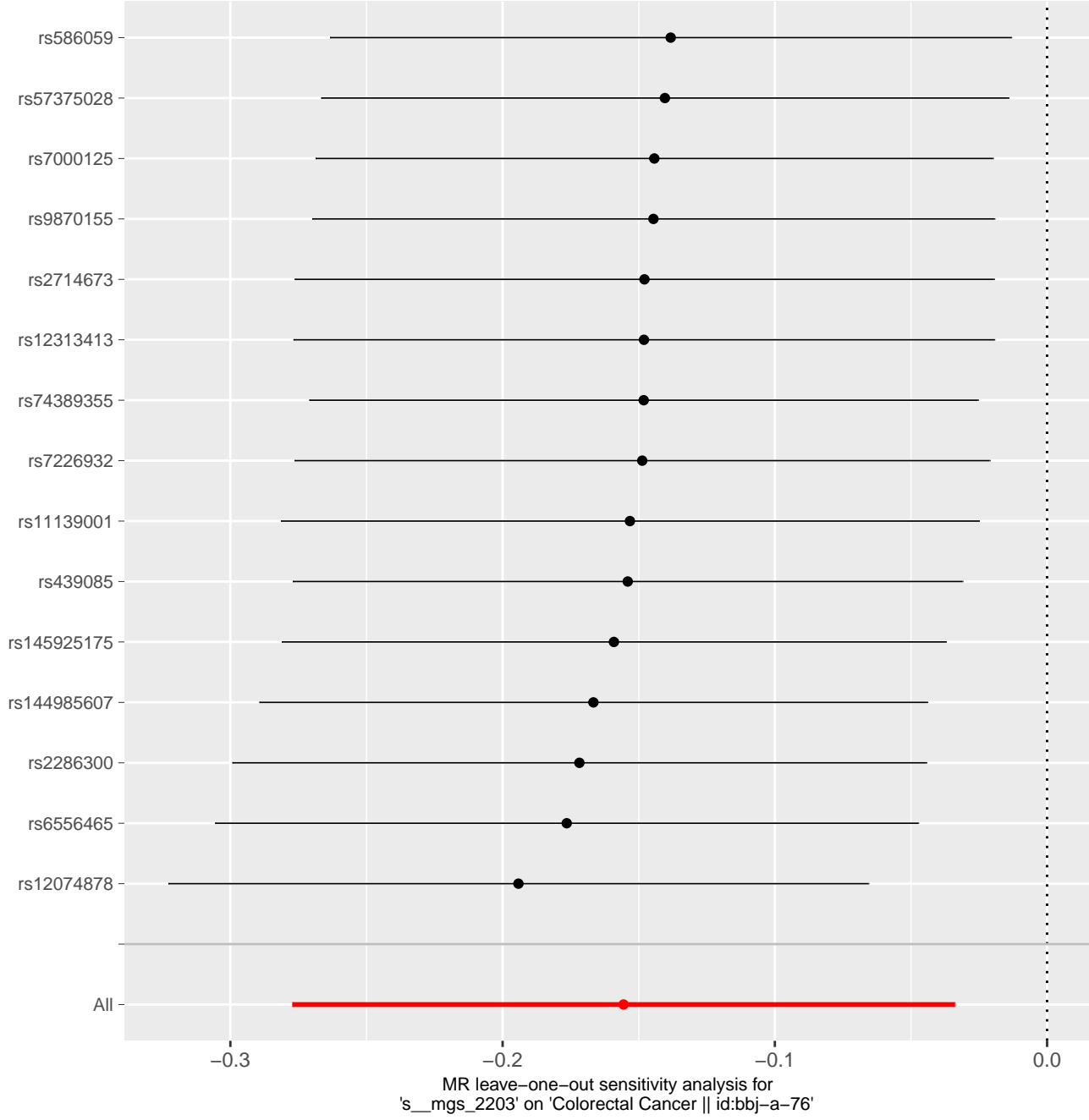

Supplement: Supplementary file 2 [file Supplementaryfile2.zip › Supplementary files 2 leave-one-out plot/saliva-pheno.87.bbj-a-76.pdf]

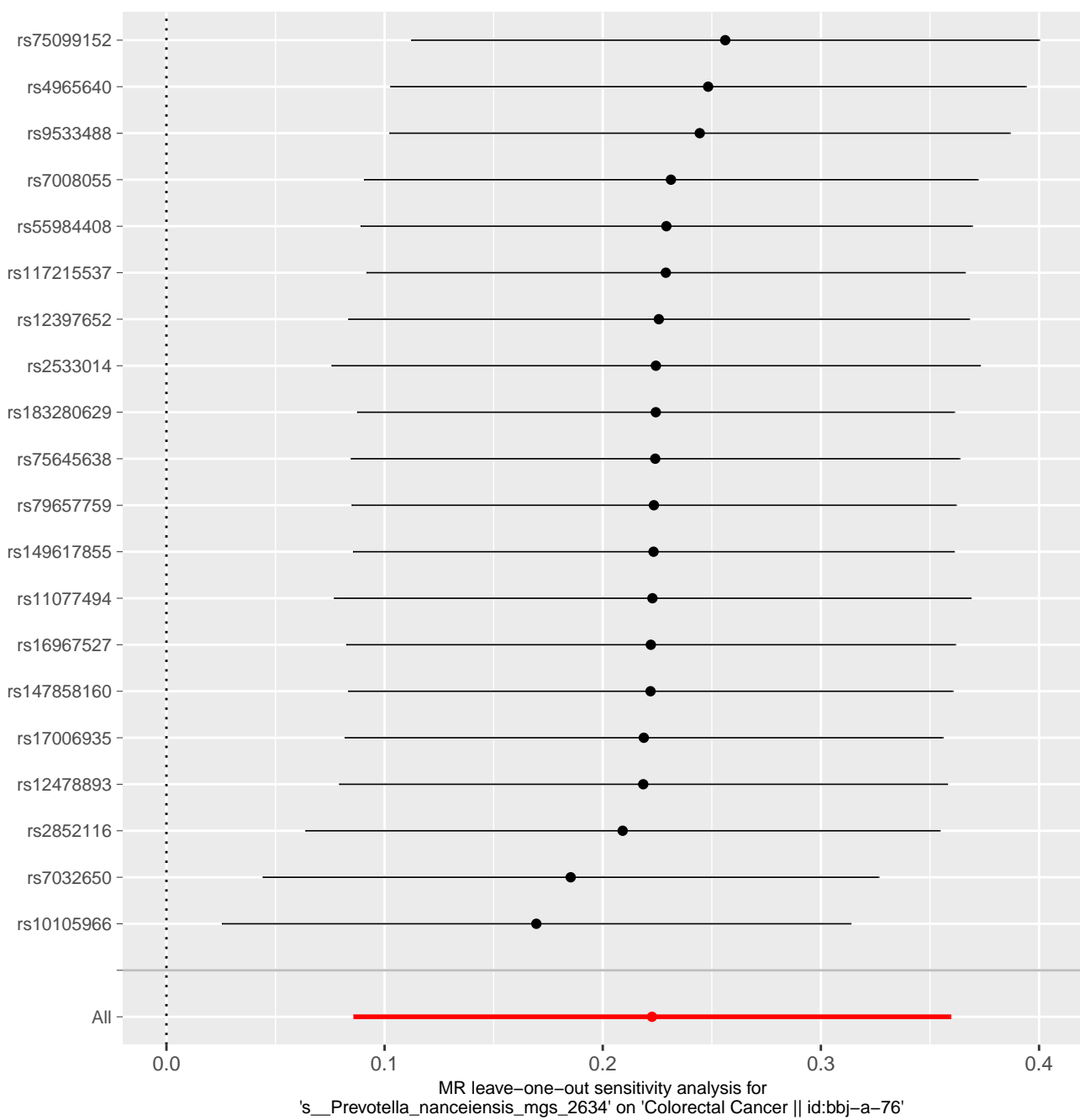

Supplement: Supplementary file 2 [file Supplementaryfile2.zip › Supplementary files 2 leave-one-out plot/tongue-pheno.1229.bbj-a-76.pdf]

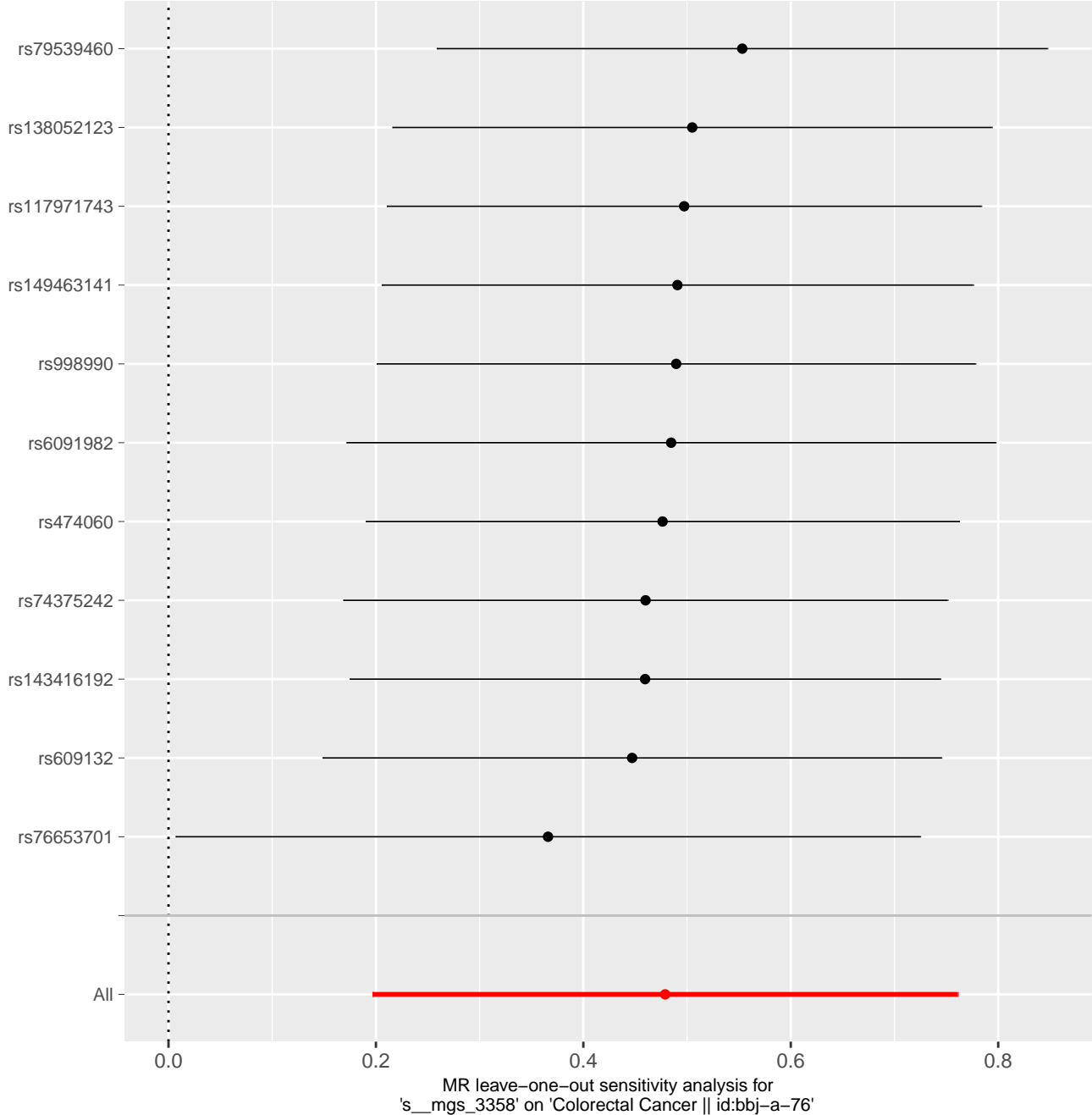

Supplement: Supplementary file 2 [file Supplementaryfile2.zip › Supplementary files 2 leave-one-out plot/tongue-pheno.145.bbj-a-76.pdf]

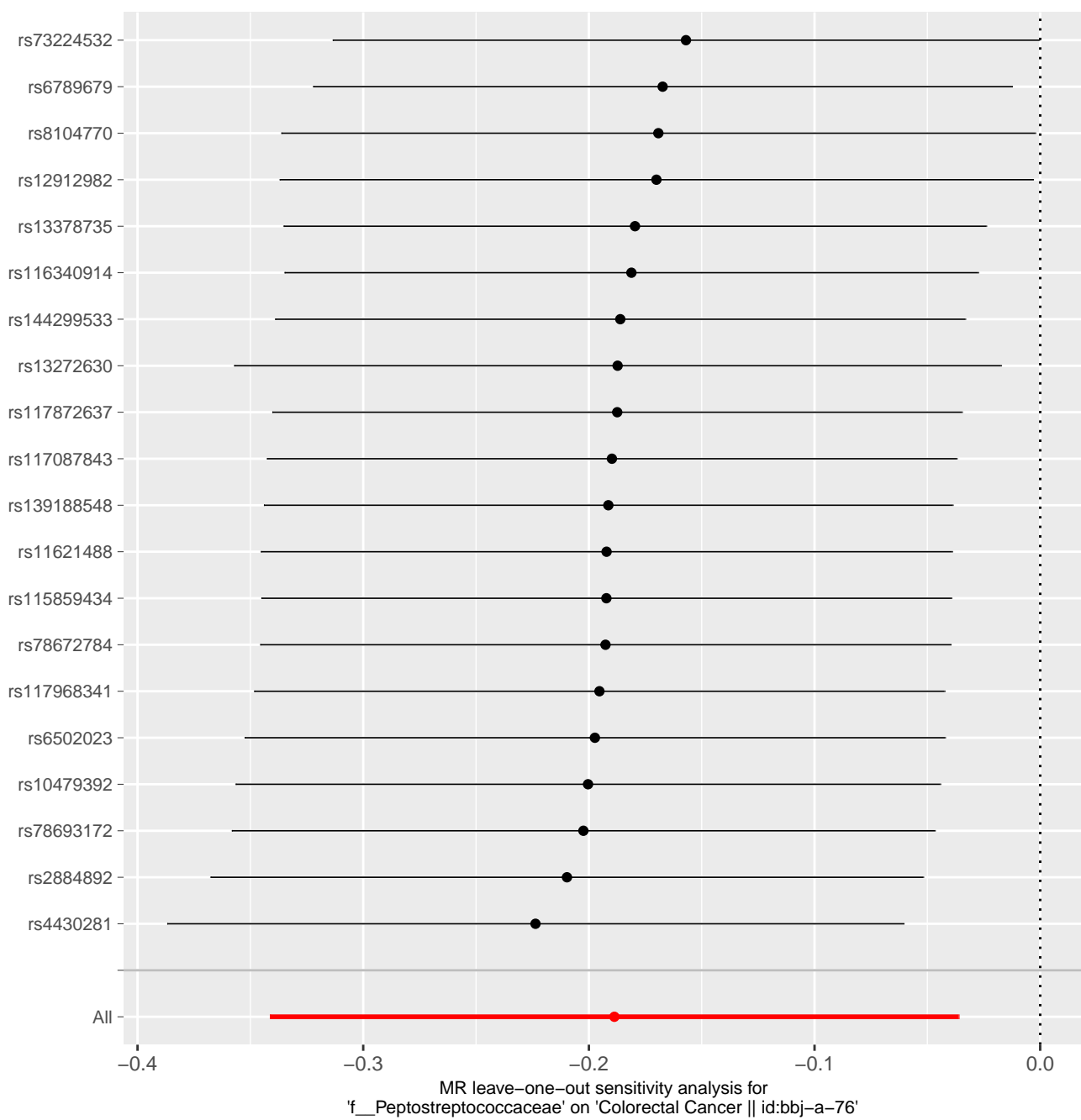

Supplement: Supplementary file 2 [file Supplementaryfile2.zip › Supplementary files 2 leave-one-out plot/tongue-pheno.1637.bbj-a-76.pdf]

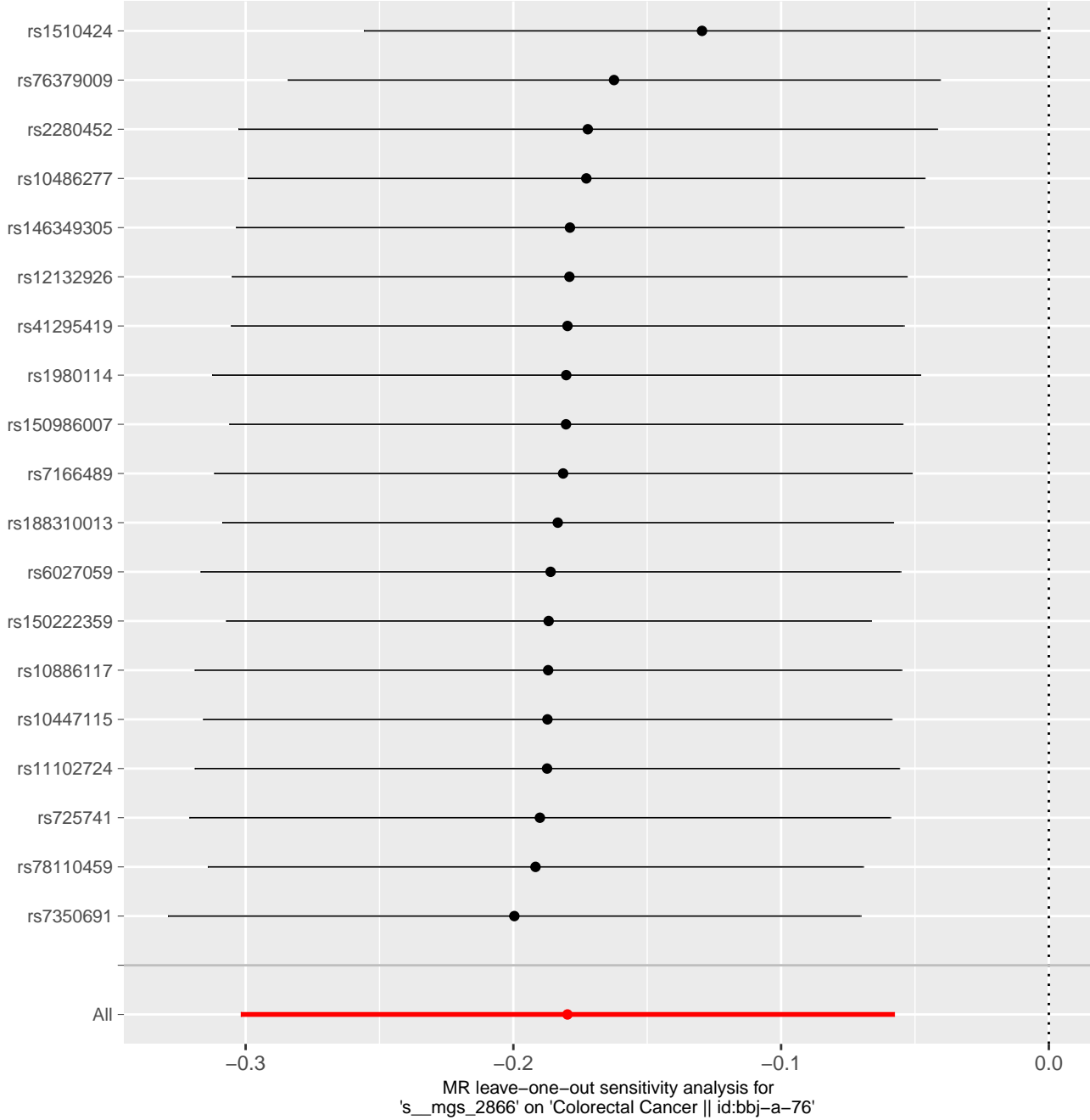

Supplement: Supplementary file 2 [file Supplementaryfile2.zip › Supplementary files 2 leave-one-out plot/tongue-pheno.1653.bbj-a-76.pdf]

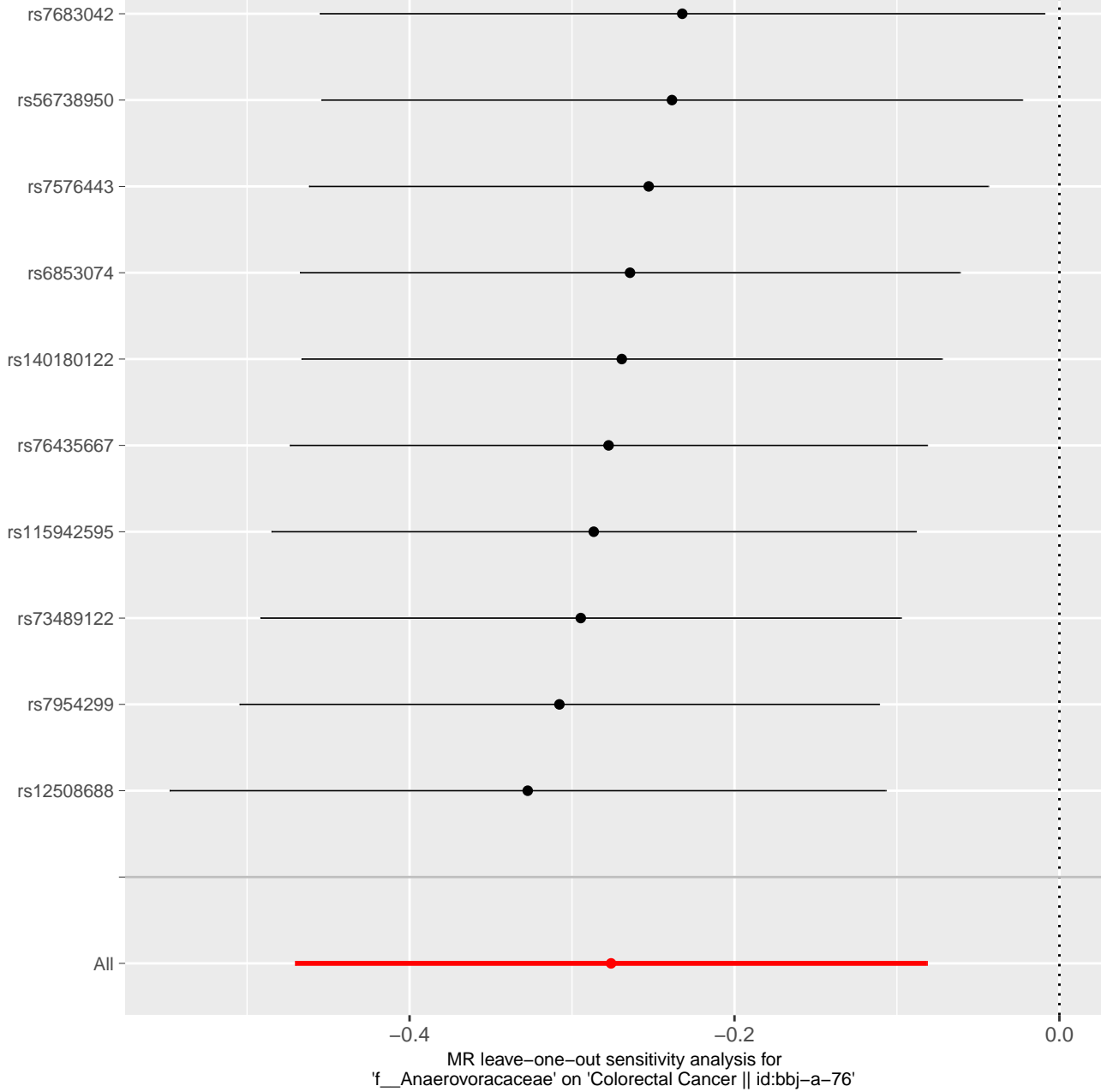

Supplement: Supplementary file 2 [file Supplementaryfile2.zip › Supplementary files 2 leave-one-out plot/tongue-pheno.1781.bbj-a-76.pdf]

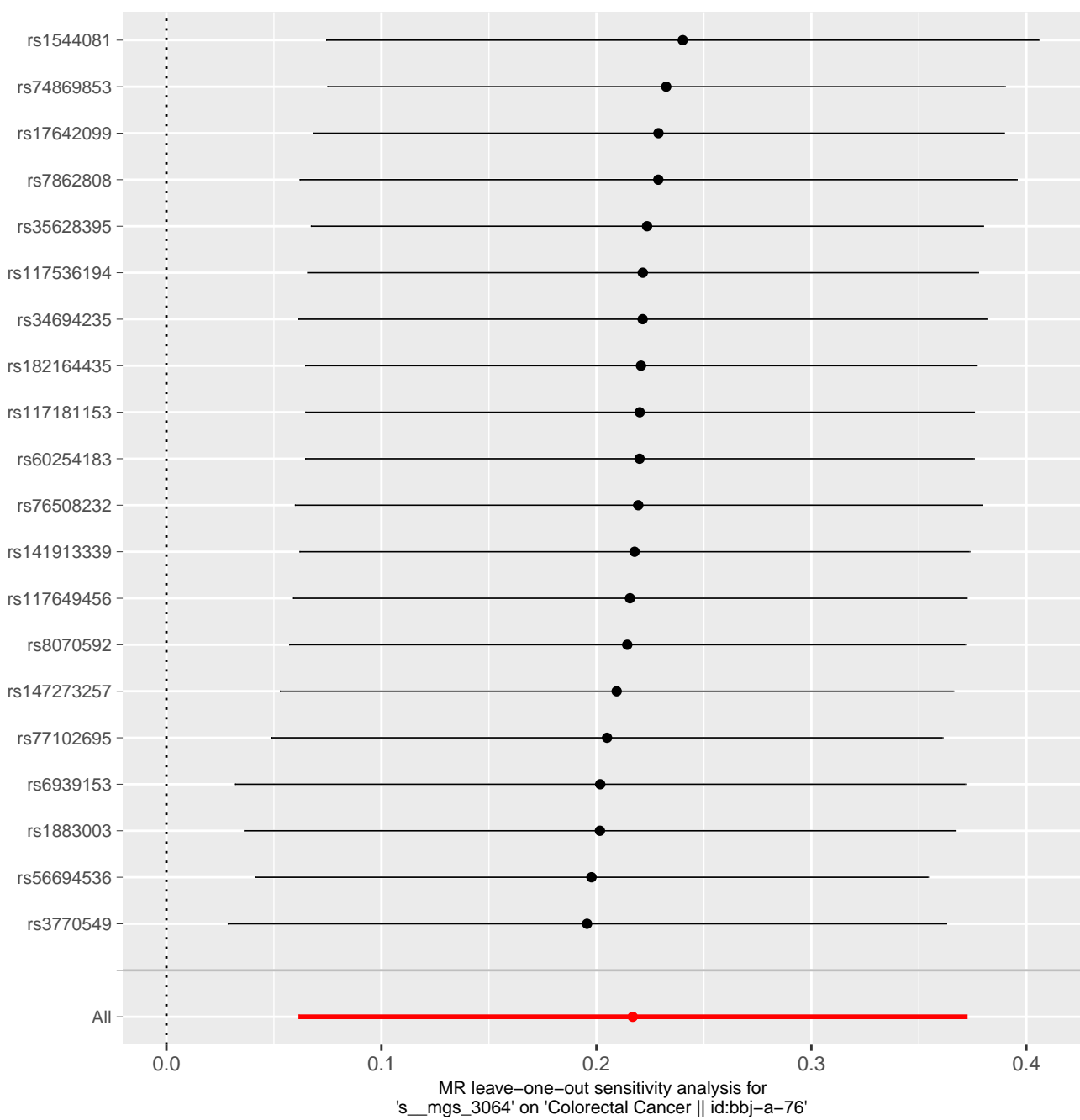

Supplement: Supplementary file 2 [file Supplementaryfile2.zip › Supplementary files 2 leave-one-out plot/tongue-pheno.3410.bbj-a-76.pdf]

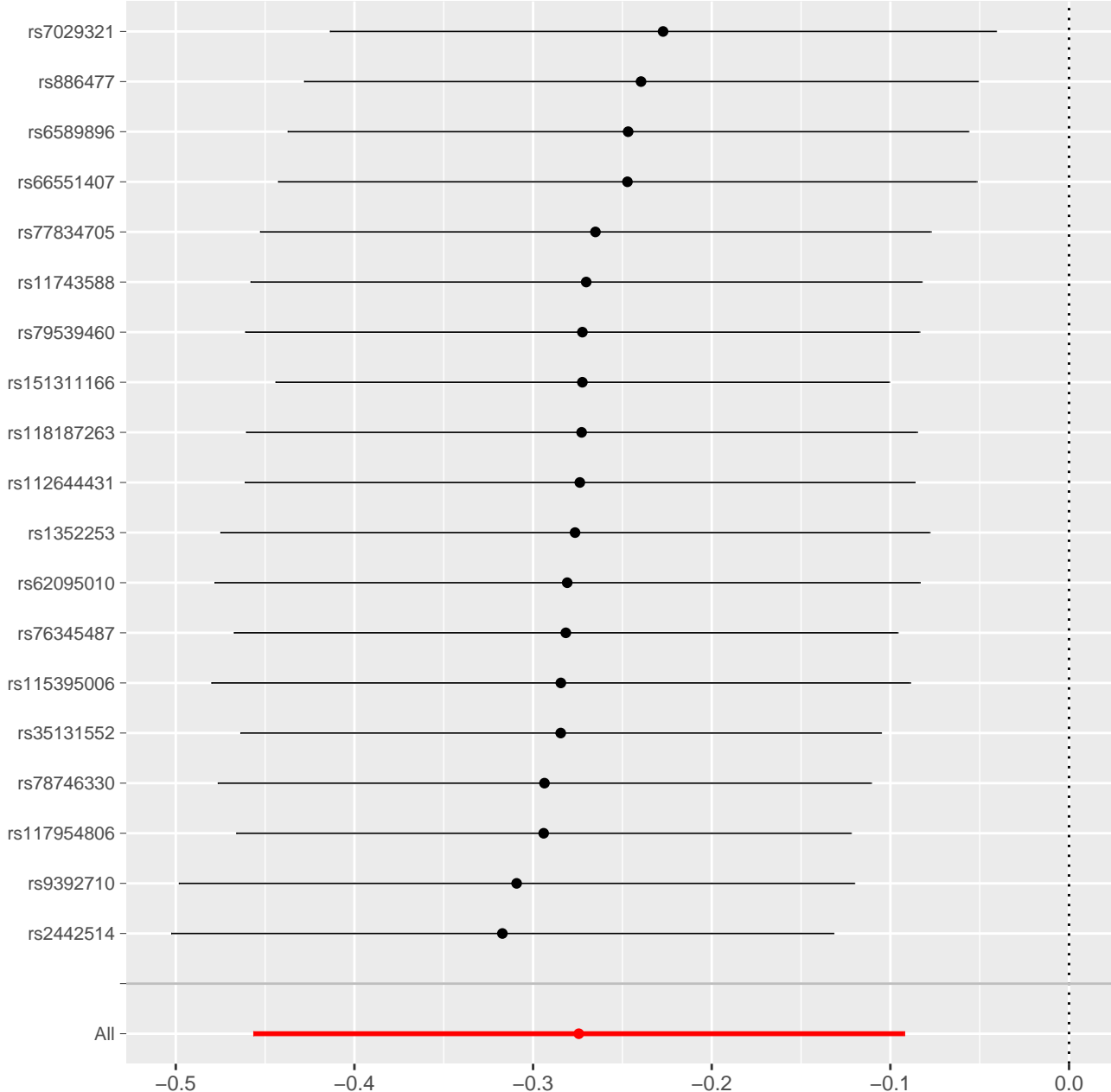

MR leave-one-out sensitivity analysis for  
's\_mgs\_2717' on 'Colorectal Cancer || id:bbj-a-76'

Supplement: Supplementary file 2 [file Supplementaryfile2.zip › Supplementary files 2 leave-one-out plot/tongue-pheno.368.bbj-a-76.pdf]

# MR Method

- Inverse variance weighted
- MR Egger

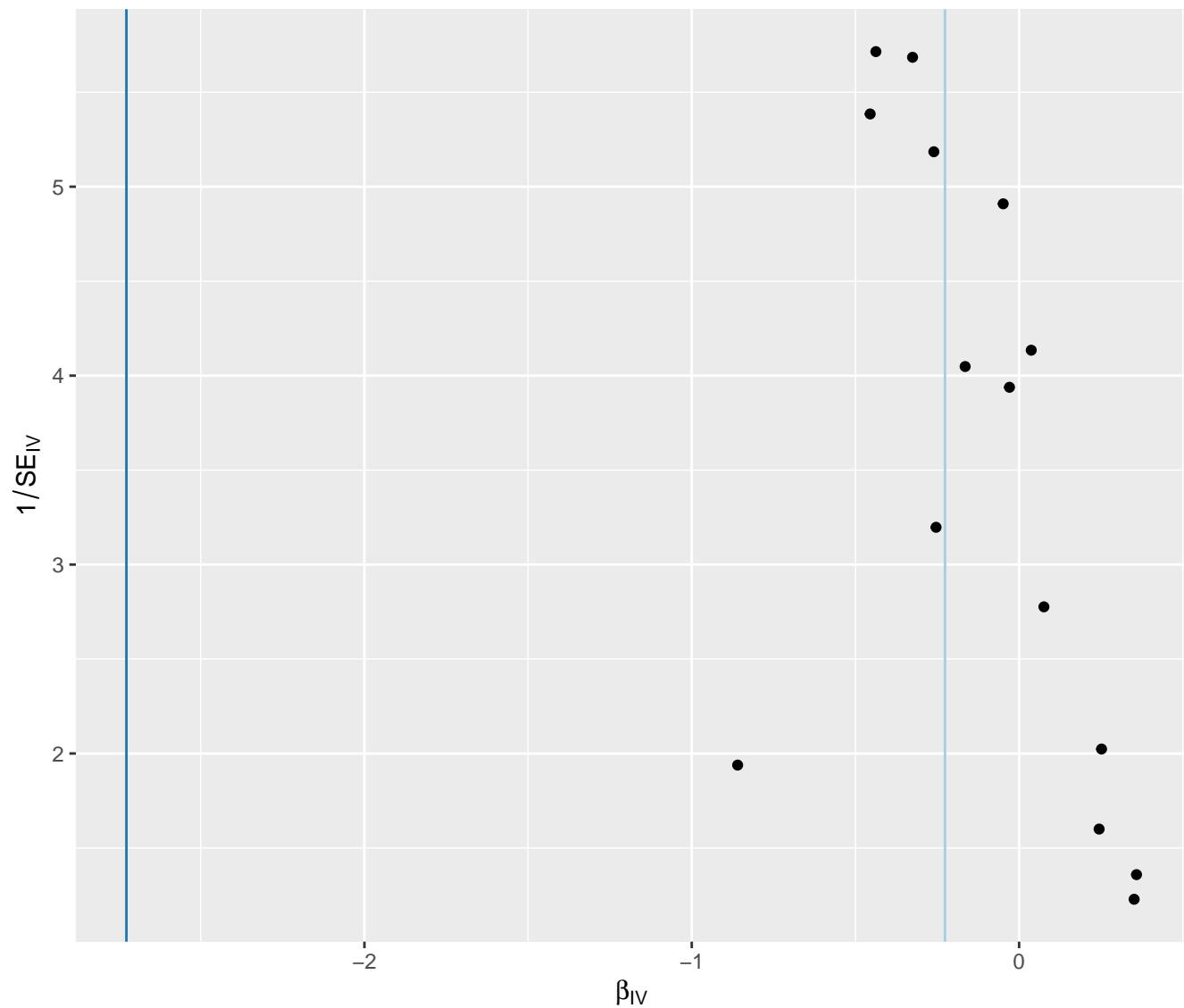

Supplement: Supplementary file 3 [file Supplementaryfile3.zip › Supplementary files 3 funnel plot/saliva-pheno.1269.bbj-a-76.pdf]

# MR Method

- Inverse variance weighted
- MR Egger

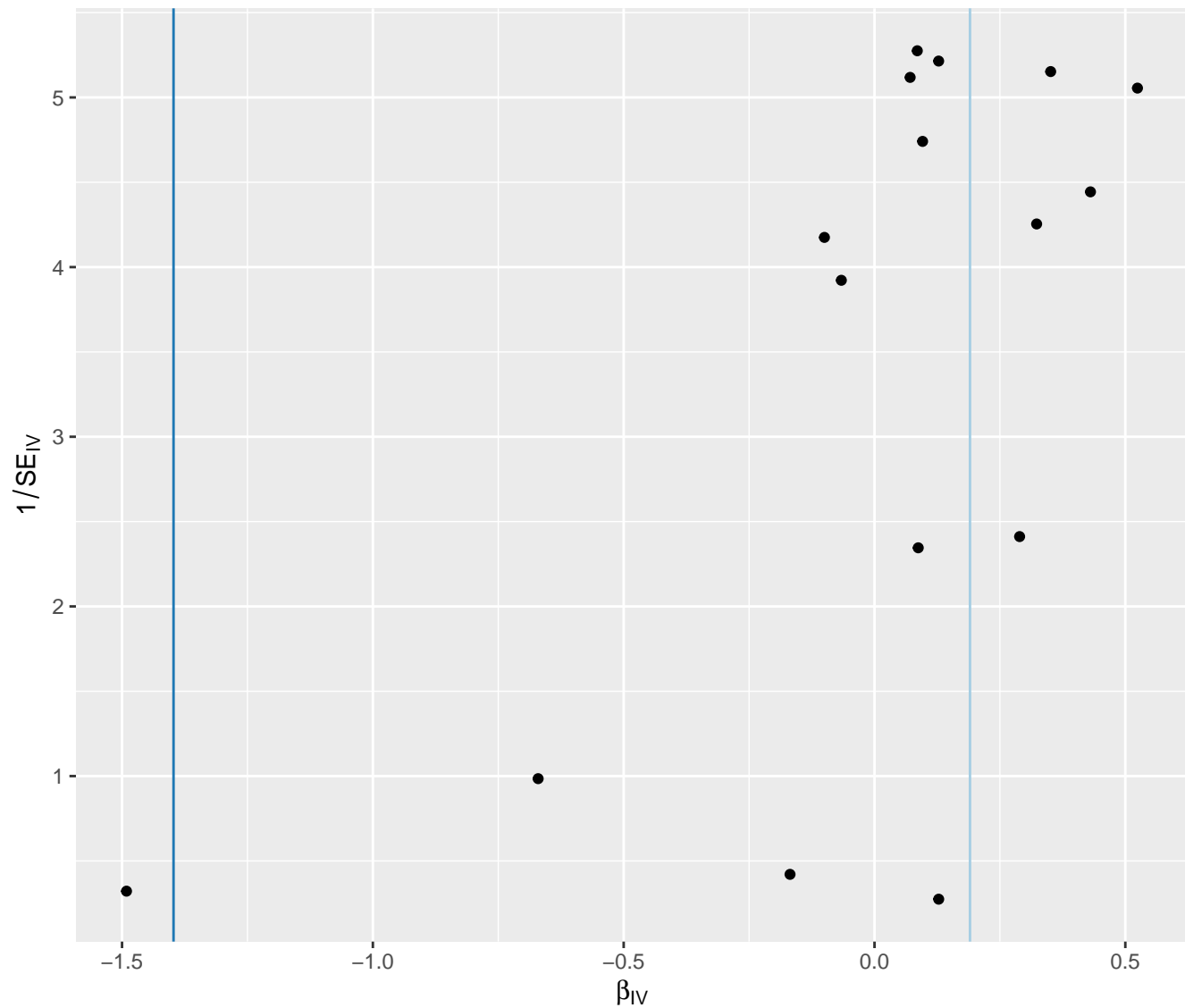

Supplement: Supplementary file 3 [file Supplementaryfile3.zip › Supplementary files 3 funnel plot/saliva-pheno.1542.bbj-a-76.pdf]

# MR Method

- Inverse variance weighted
- MR Egger

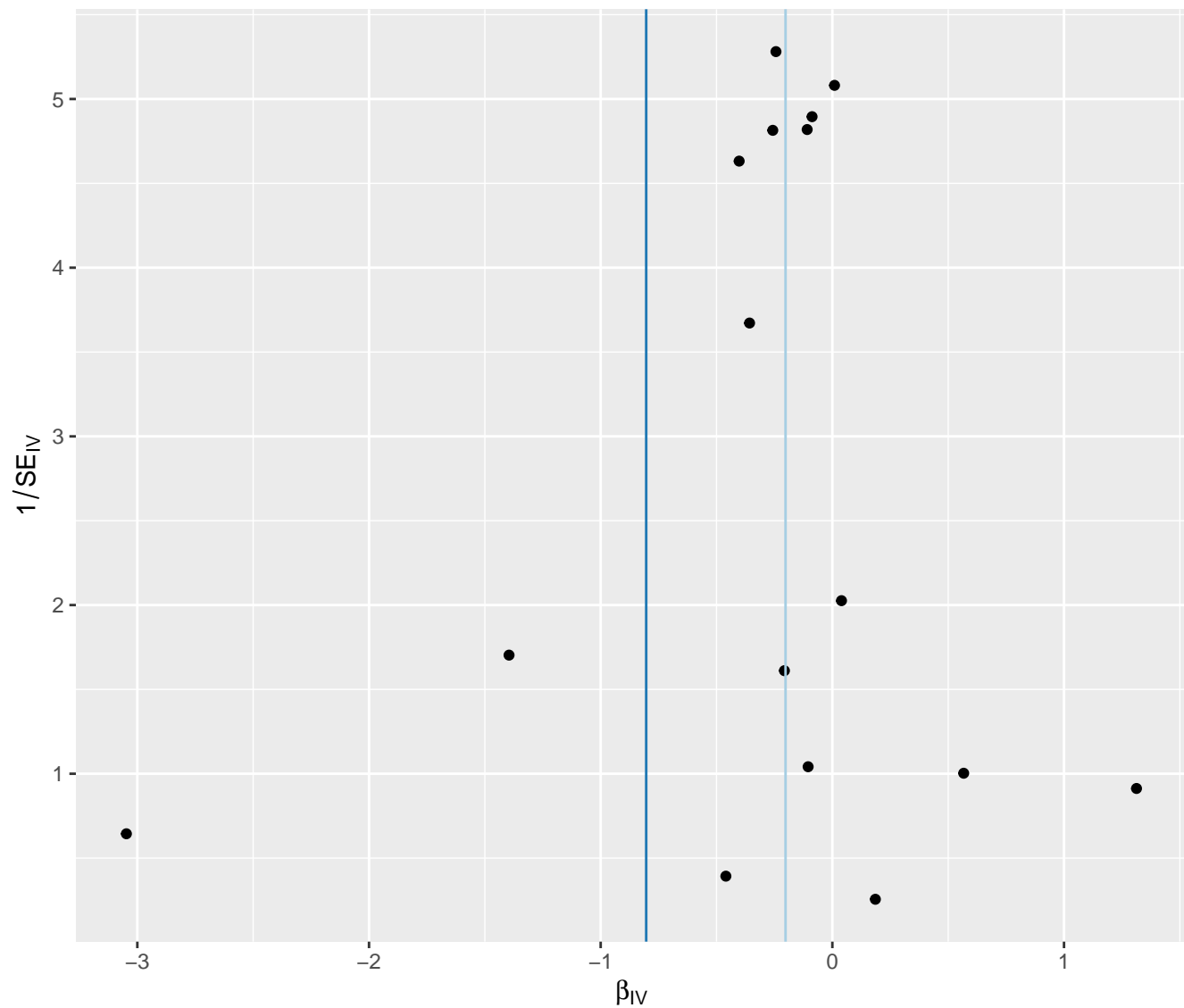

Supplement: Supplementary file 3 [file Supplementaryfile3.zip › Supplementary files 3 funnel plot/saliva-pheno.160.bbj-a-76.pdf]

# MR Method

- Inverse variance weighted
- MR Egger

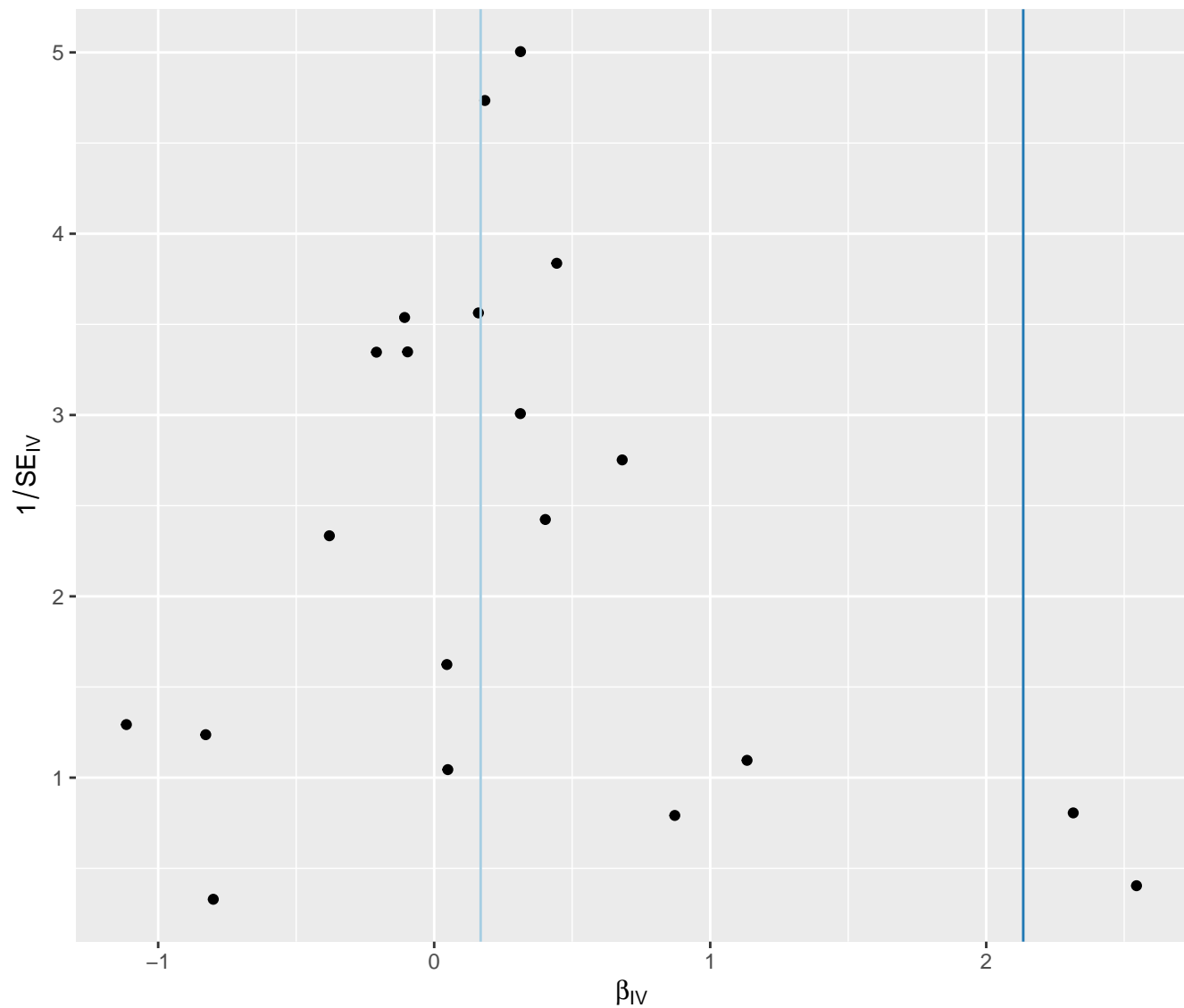

Supplement: Supplementary file 3 [file Supplementaryfile3.zip › Supplementary files 3 funnel plot/saliva-pheno.2303.bbj-a-76.pdf]

# MR Method

- Inverse variance weighted
- MR Egger

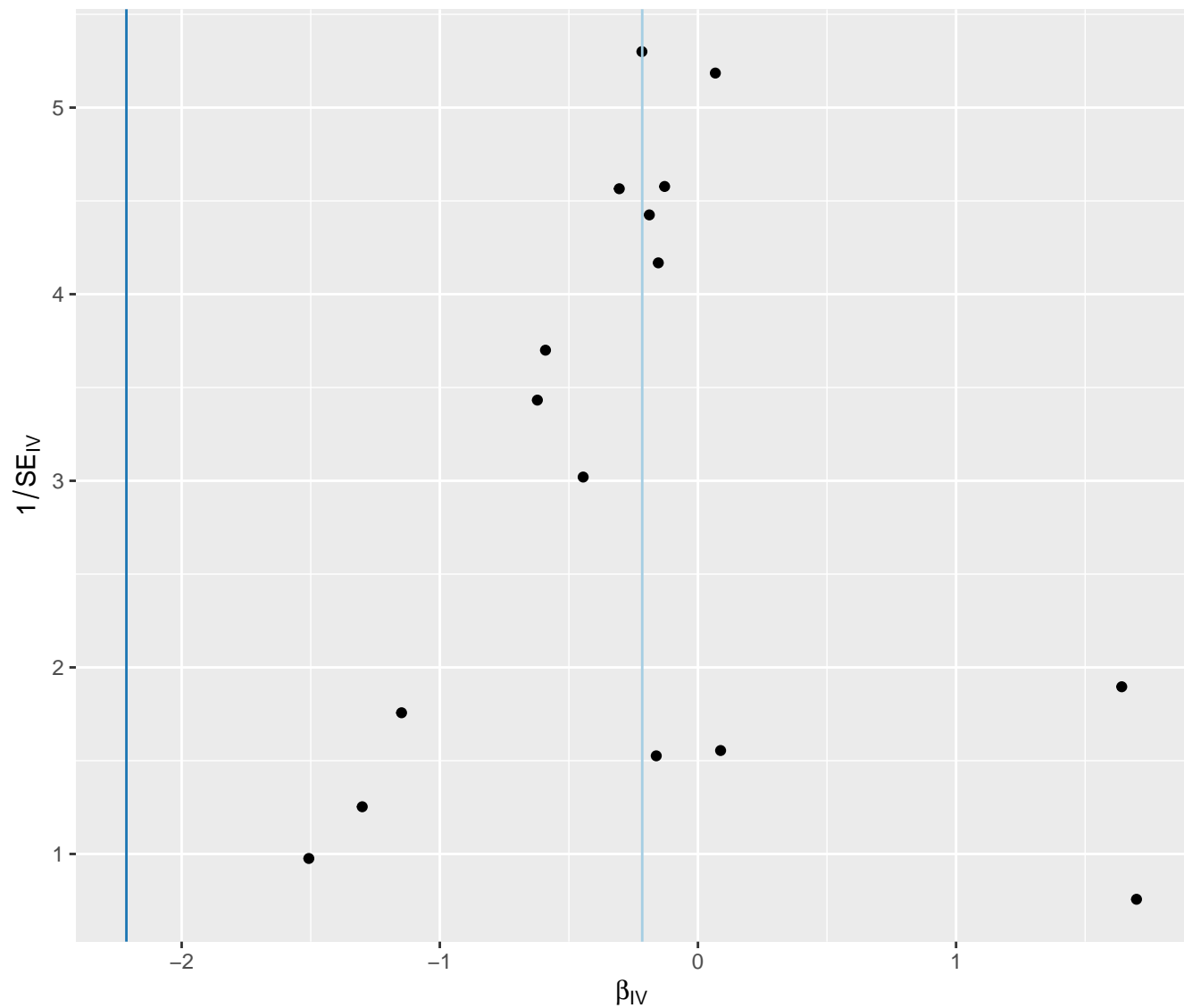

Supplement: Supplementary file 3 [file Supplementaryfile3.zip › Supplementary files 3 funnel plot/saliva-pheno.2610.bbj-a-76.pdf]

# MR Method

- Inverse variance weighted
- MR Egger

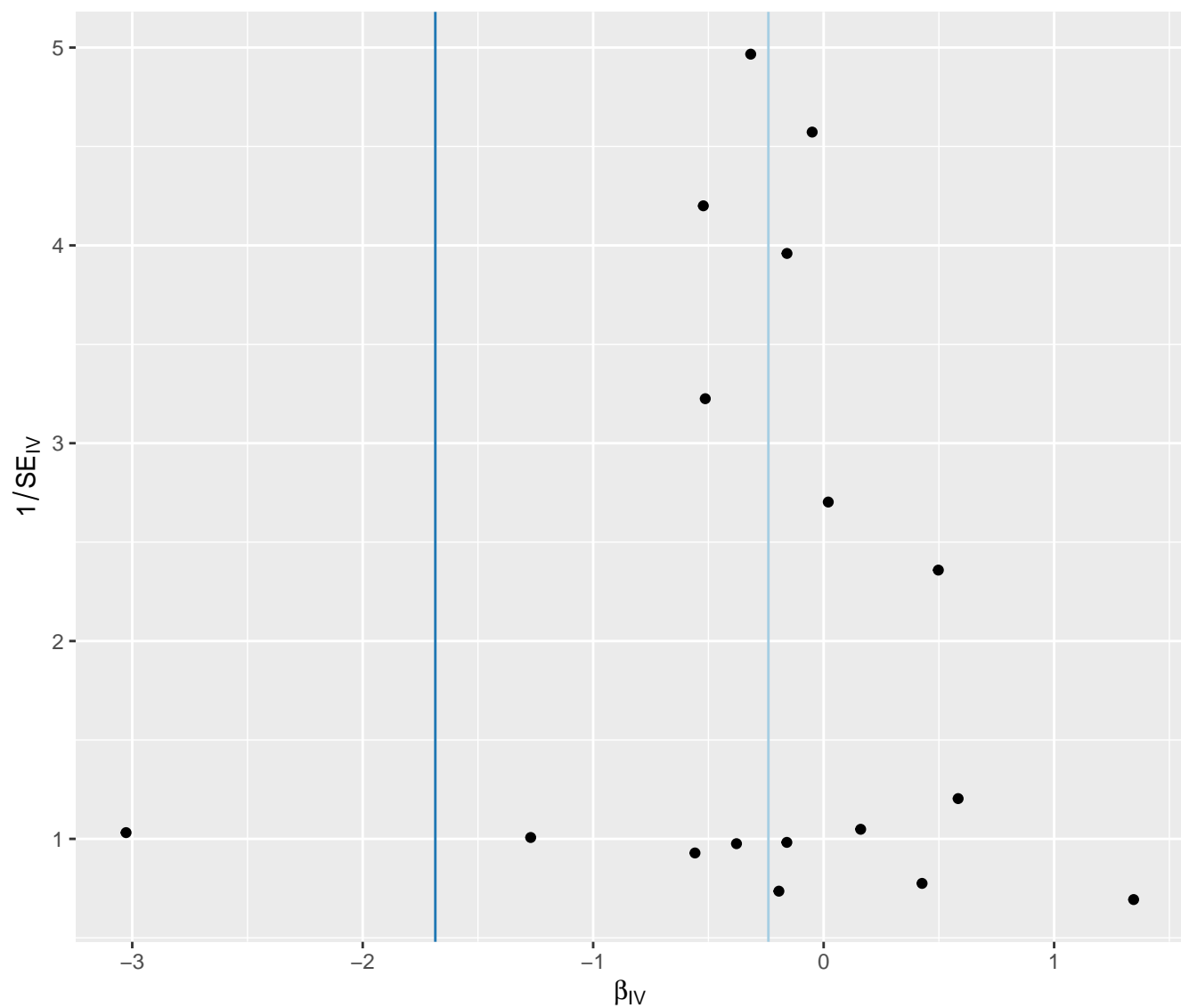

Supplement: Supplementary file 3 [file Supplementaryfile3.zip › Supplementary files 3 funnel plot/saliva-pheno.3072.bbj-a-76.pdf]

# MR Method

- Inverse variance weighted
- MR Egger

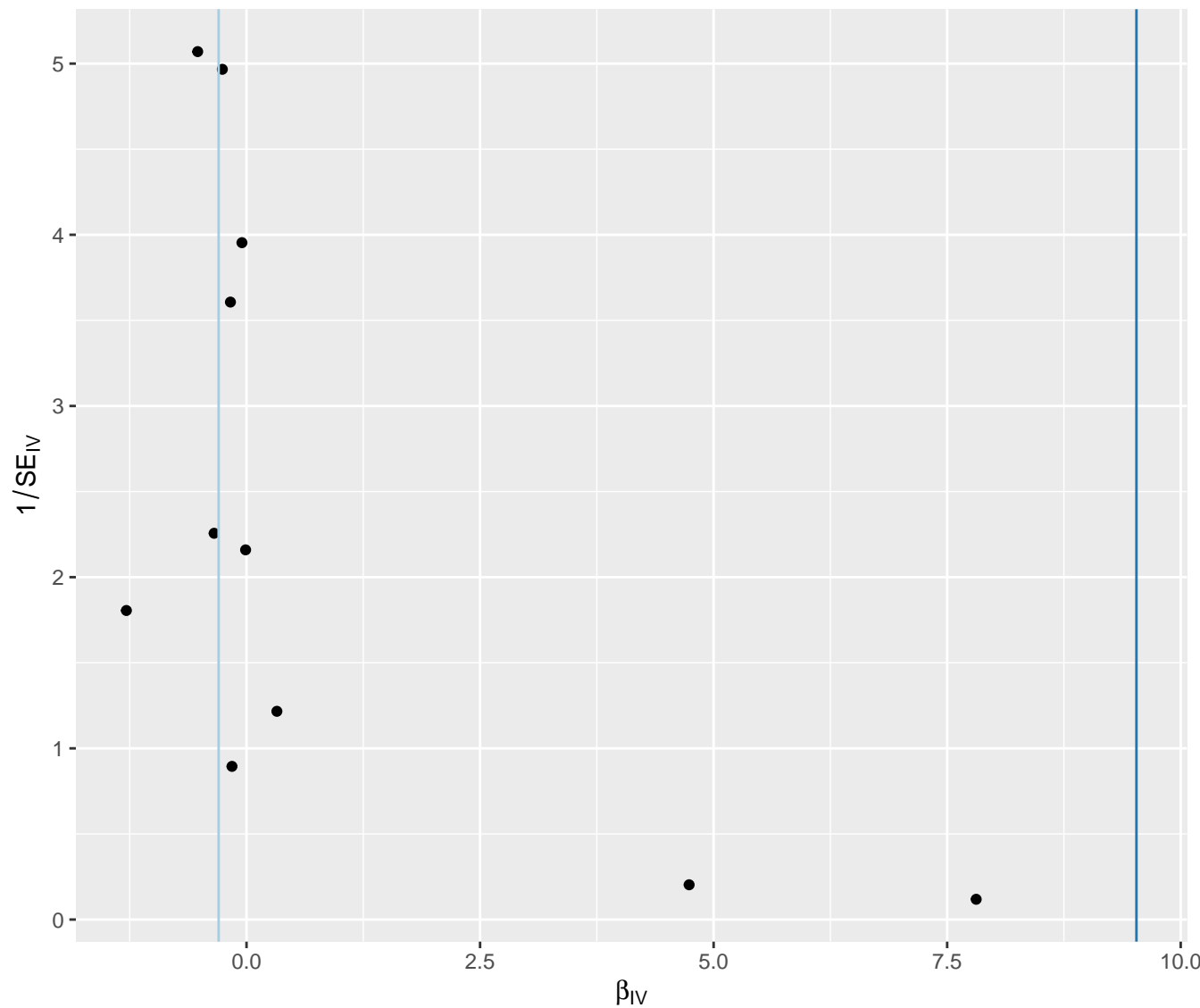

Supplement: Supplementary file 3 [file Supplementaryfile3.zip › Supplementary files 3 funnel plot/saliva-pheno.654.bbj-a-76.pdf]

# MR Method

- Inverse variance weighted
- MR Egger

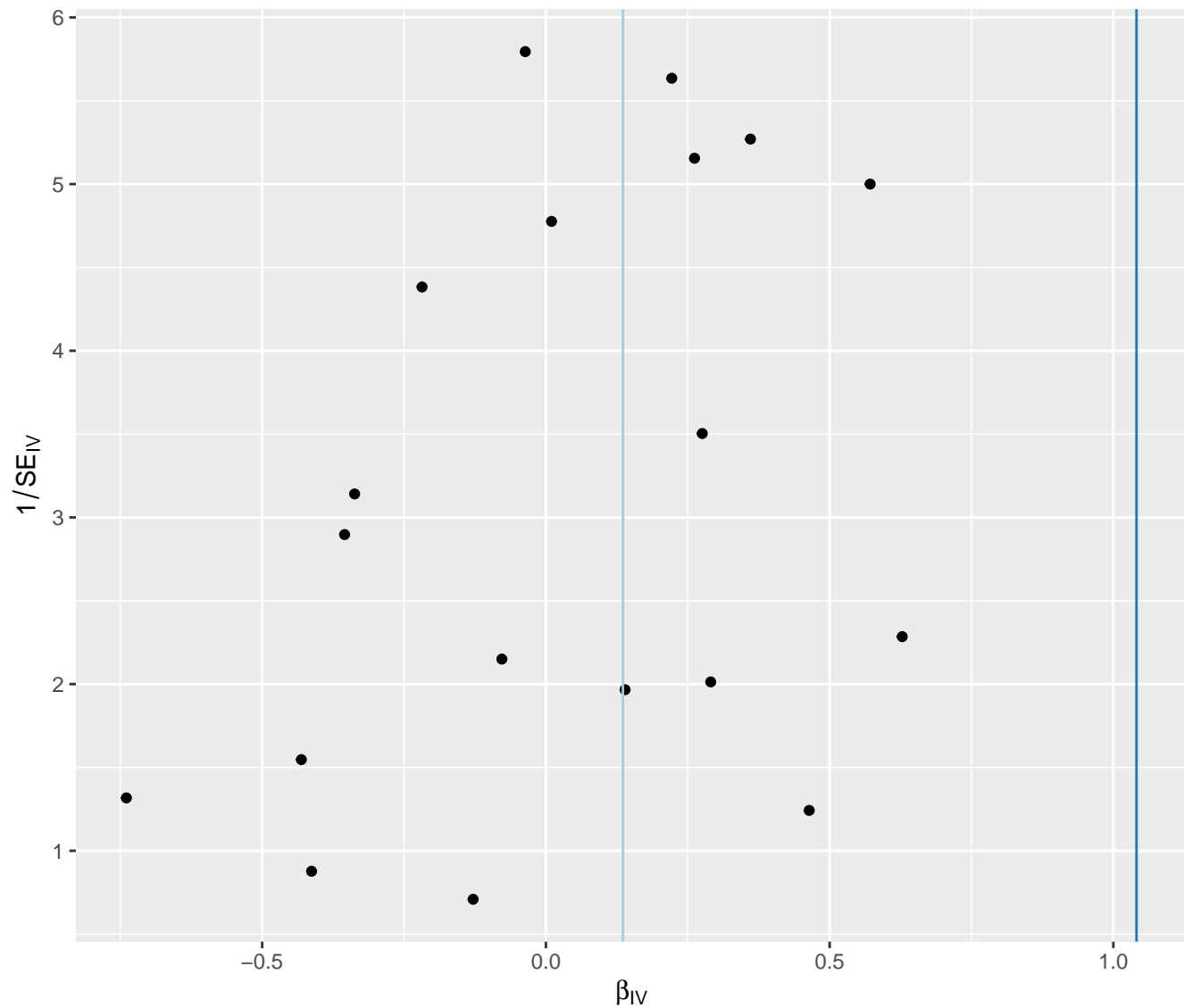

Supplement: Supplementary file 3 [file Supplementaryfile3.zip › Supplementary files 3 funnel plot/saliva-pheno.673.bbj-a-76.pdf]

# MR Method

- Inverse variance weighted
- MR Egger

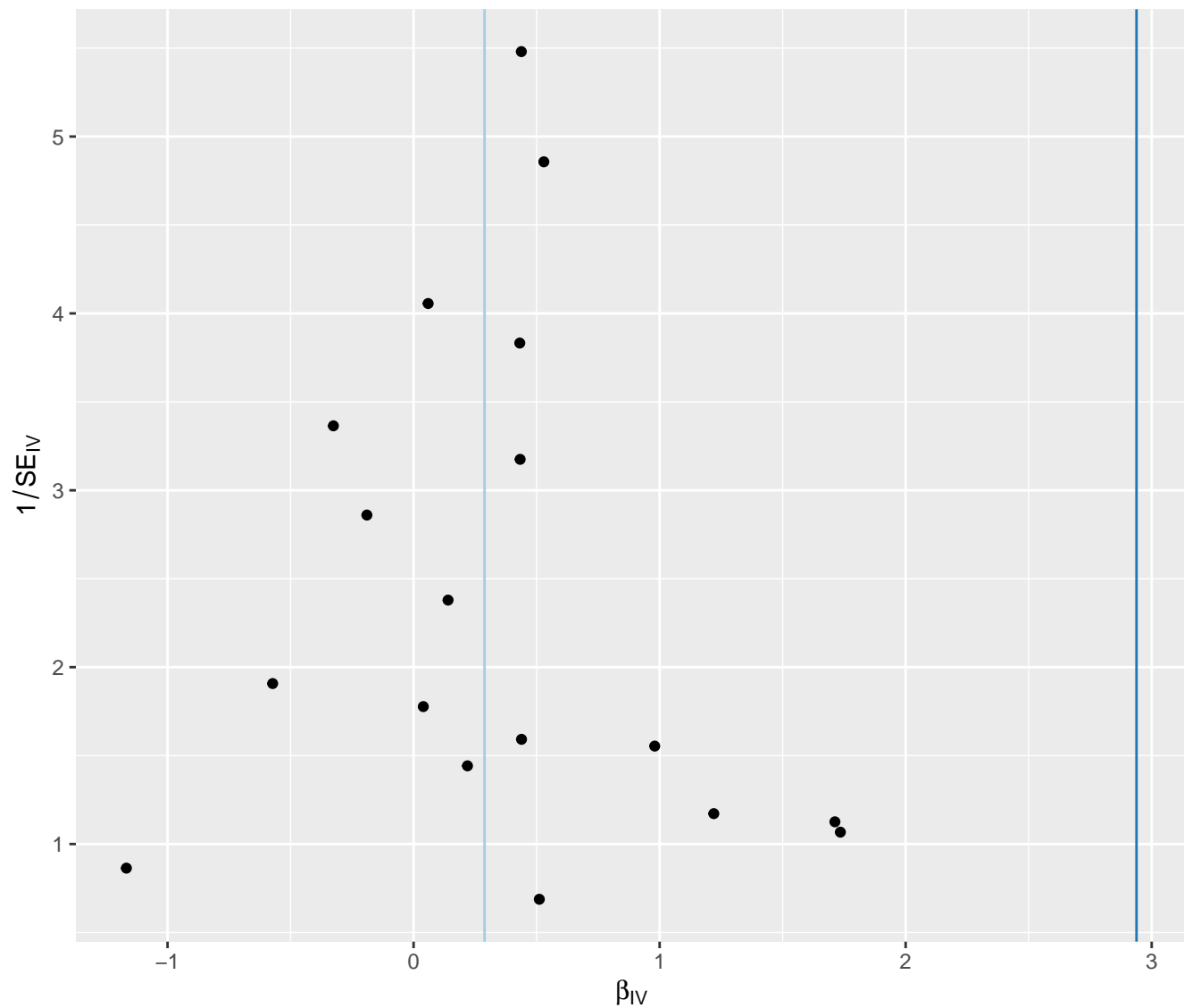

Supplement: Supplementary file 3 [file Supplementaryfile3.zip › Supplementary files 3 funnel plot/saliva-pheno.725.bbj-a-76.pdf]

# MR Method

- Inverse variance weighted
- MR Egger

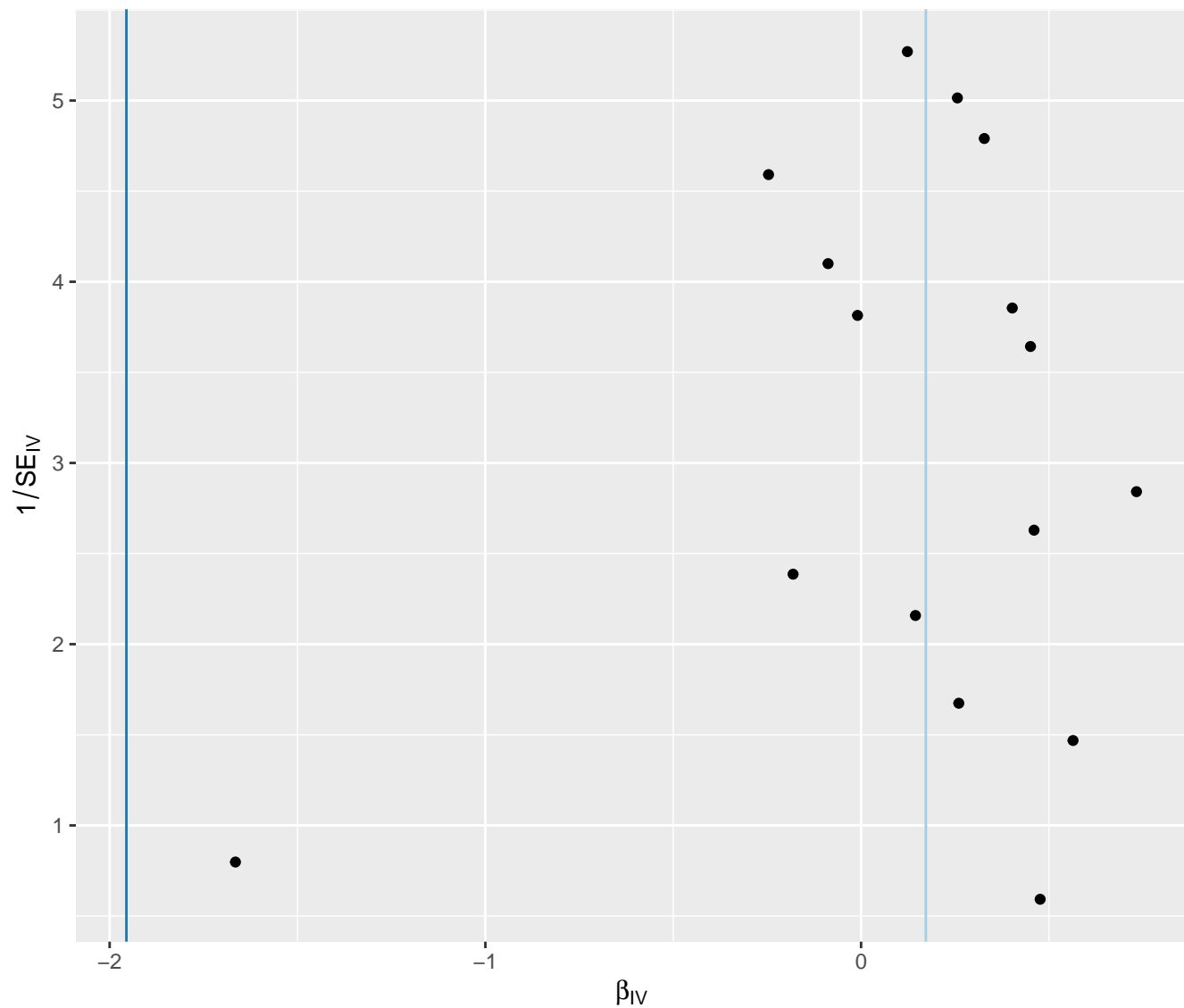

Supplement: Supplementary file 3 [file Supplementaryfile3.zip › Supplementary files 3 funnel plot/saliva-pheno.846.bbj-a-76.pdf]

# MR Method

- Inverse variance weighted
- MR Egger

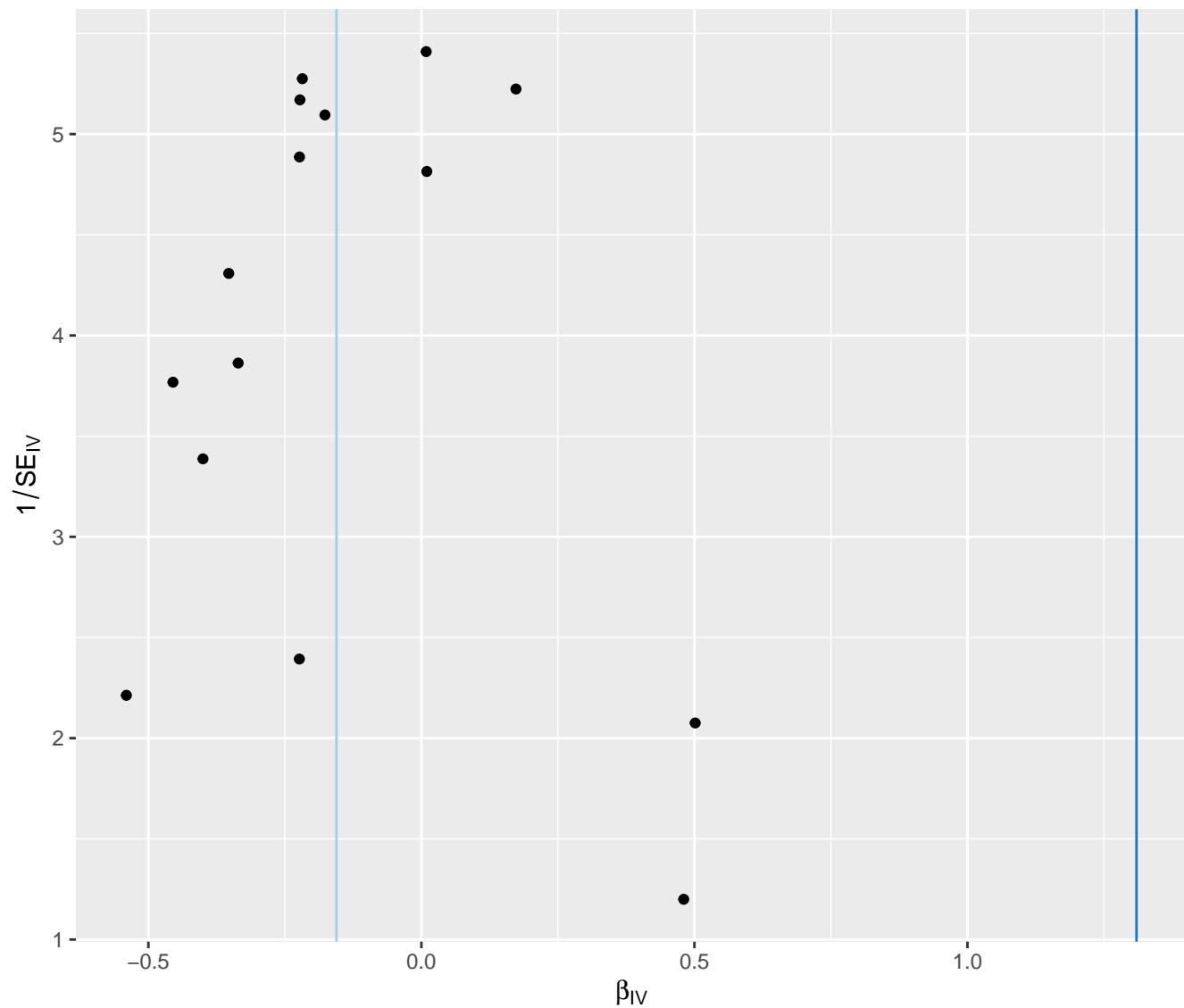

Supplement: Supplementary file 3 [file Supplementaryfile3.zip › Supplementary files 3 funnel plot/saliva-pheno.87.bbj-a-76.pdf]

# MR Method

- Inverse variance weighted
- MR Egger

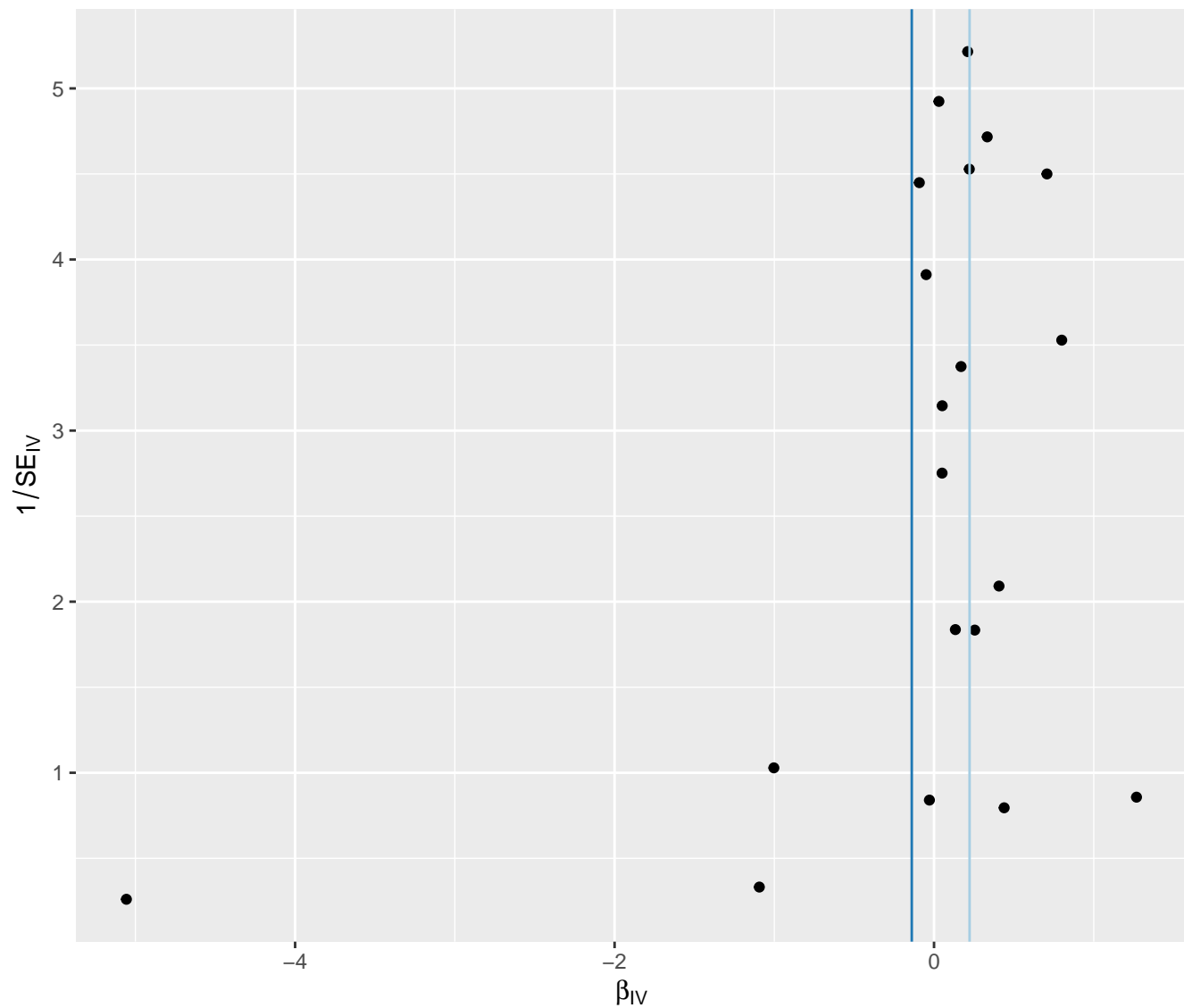

Supplement: Supplementary file 3 [file Supplementaryfile3.zip › Supplementary files 3 funnel plot/tongue-pheno.1229.bbj-a-76.pdf]

# MR Method

- Inverse variance weighted
- MR Egger

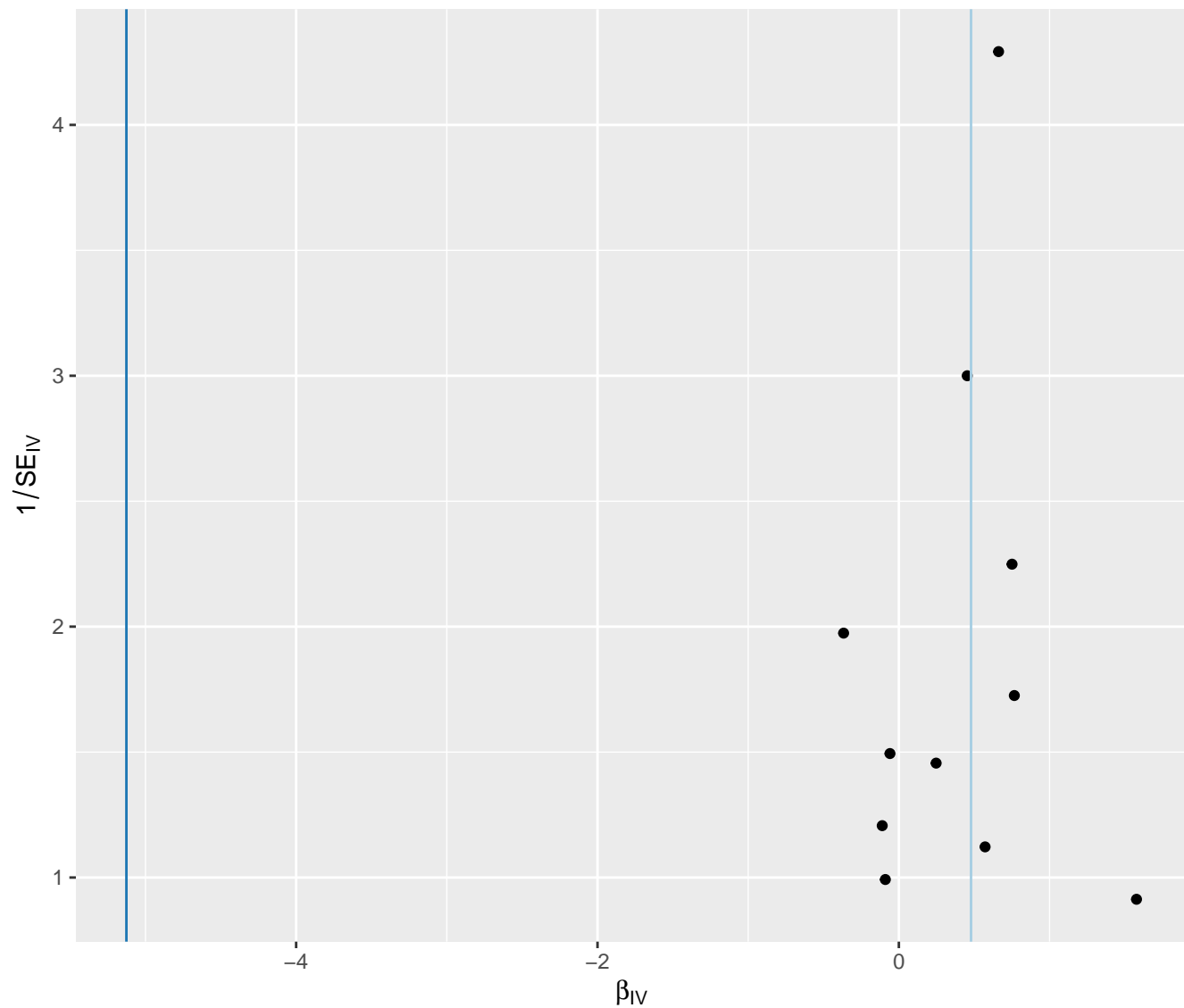

Supplement: Supplementary file 3 [file Supplementaryfile3.zip › Supplementary files 3 funnel plot/tongue-pheno.145.bbj-a-76.pdf]

# MR Method

- Inverse variance weighted
- MR Egger

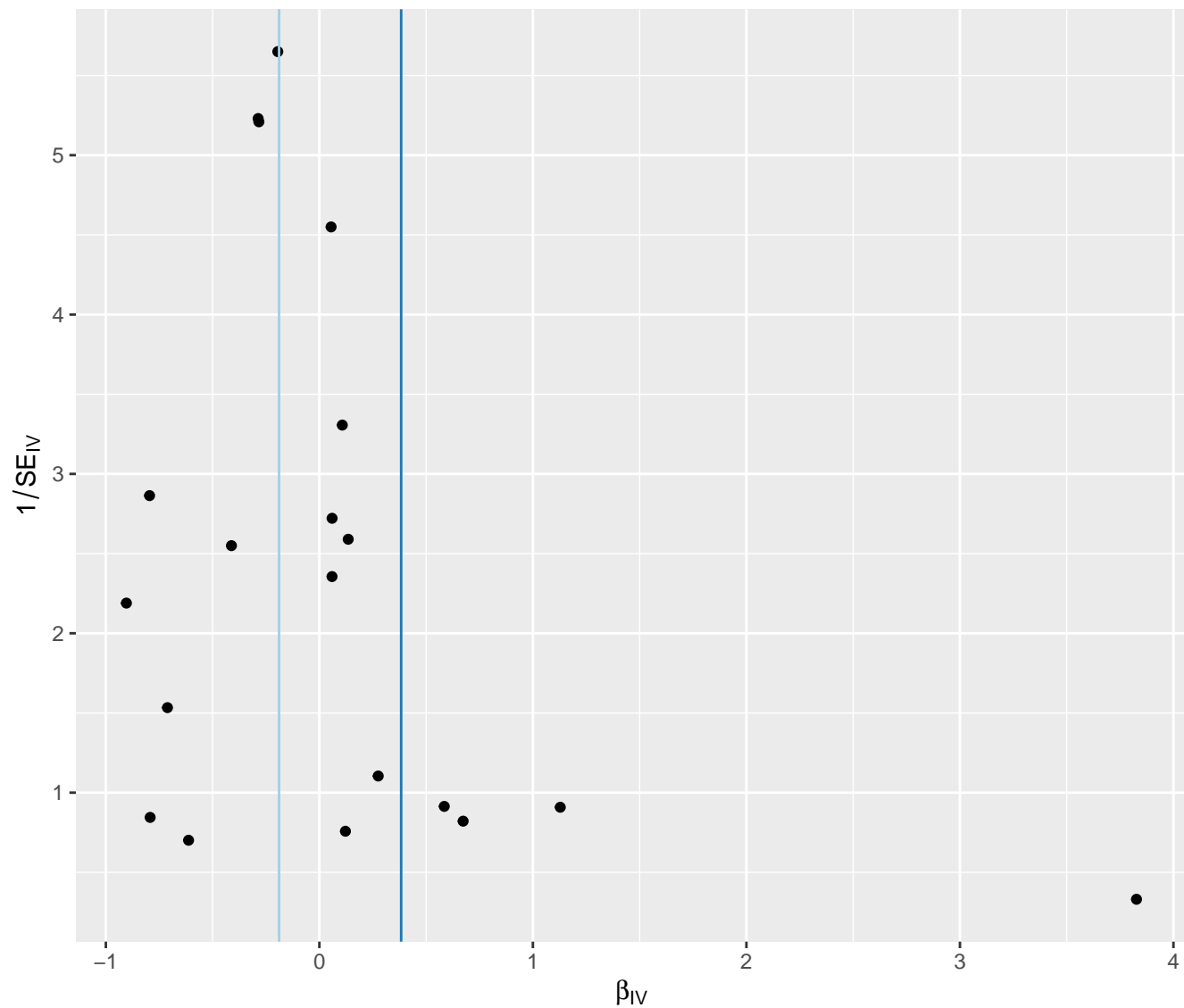

Supplement: Supplementary file 3 [file Supplementaryfile3.zip › Supplementary files 3 funnel plot/tongue-pheno.1637.bbj-a-76.pdf]

# MR Method

- Inverse variance weighted
- MR Egger

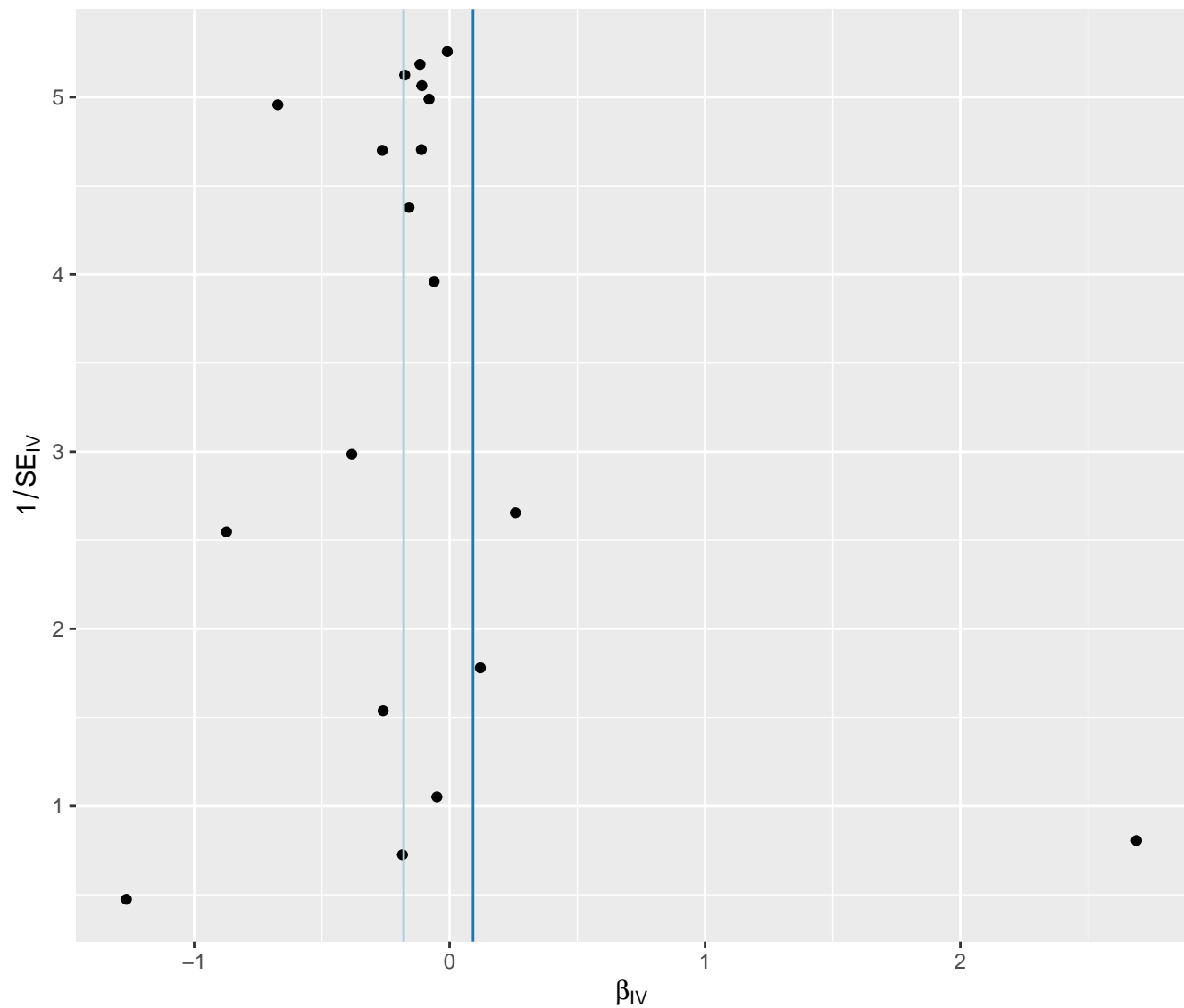

Supplement: Supplementary file 3 [file Supplementaryfile3.zip › Supplementary files 3 funnel plot/tongue-pheno.1653.bbj-a-76.pdf]

# MR Method

- Inverse variance weighted
- MR Egger

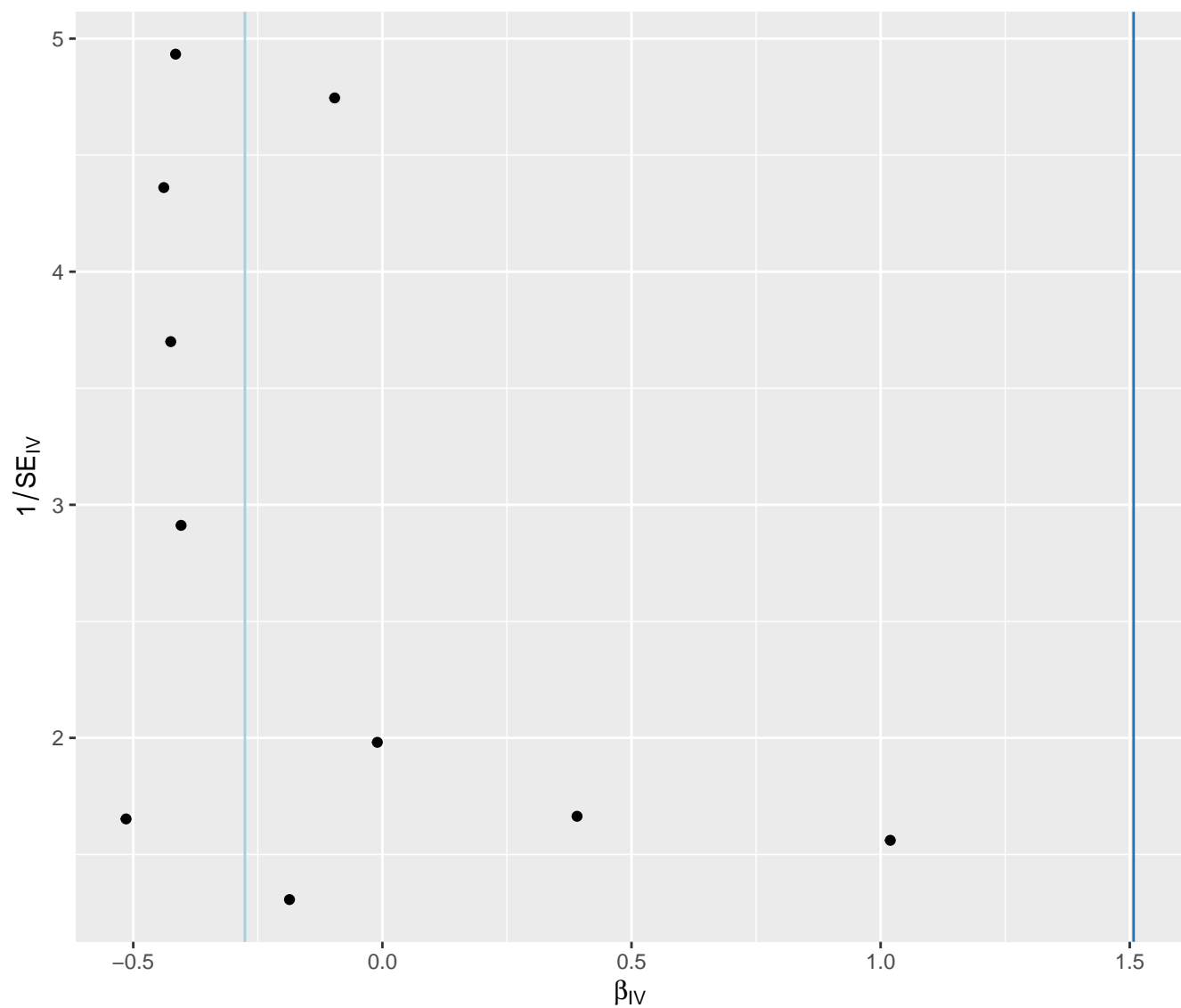

Supplement: Supplementary file 3 [file Supplementaryfile3.zip › Supplementary files 3 funnel plot/tongue-pheno.1781.bbj-a-76.pdf]

# MR Method

- Inverse variance weighted
- MR Egger

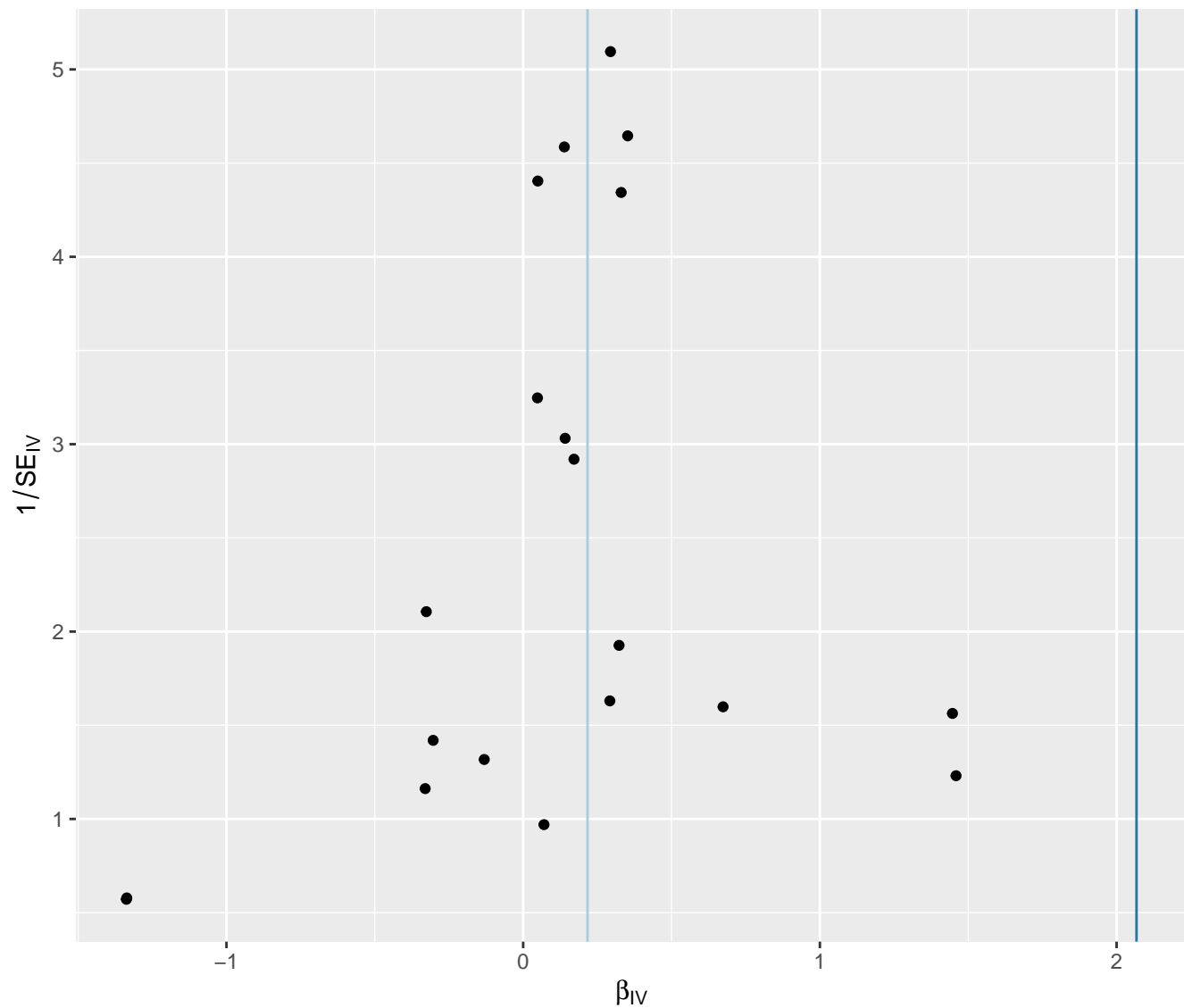

Supplement: Supplementary file 3 [file Supplementaryfile3.zip › Supplementary files 3 funnel plot/tongue-pheno.3410.bbj-a-76.pdf]

# MR Method

- Inverse variance weighted
- MR Egger

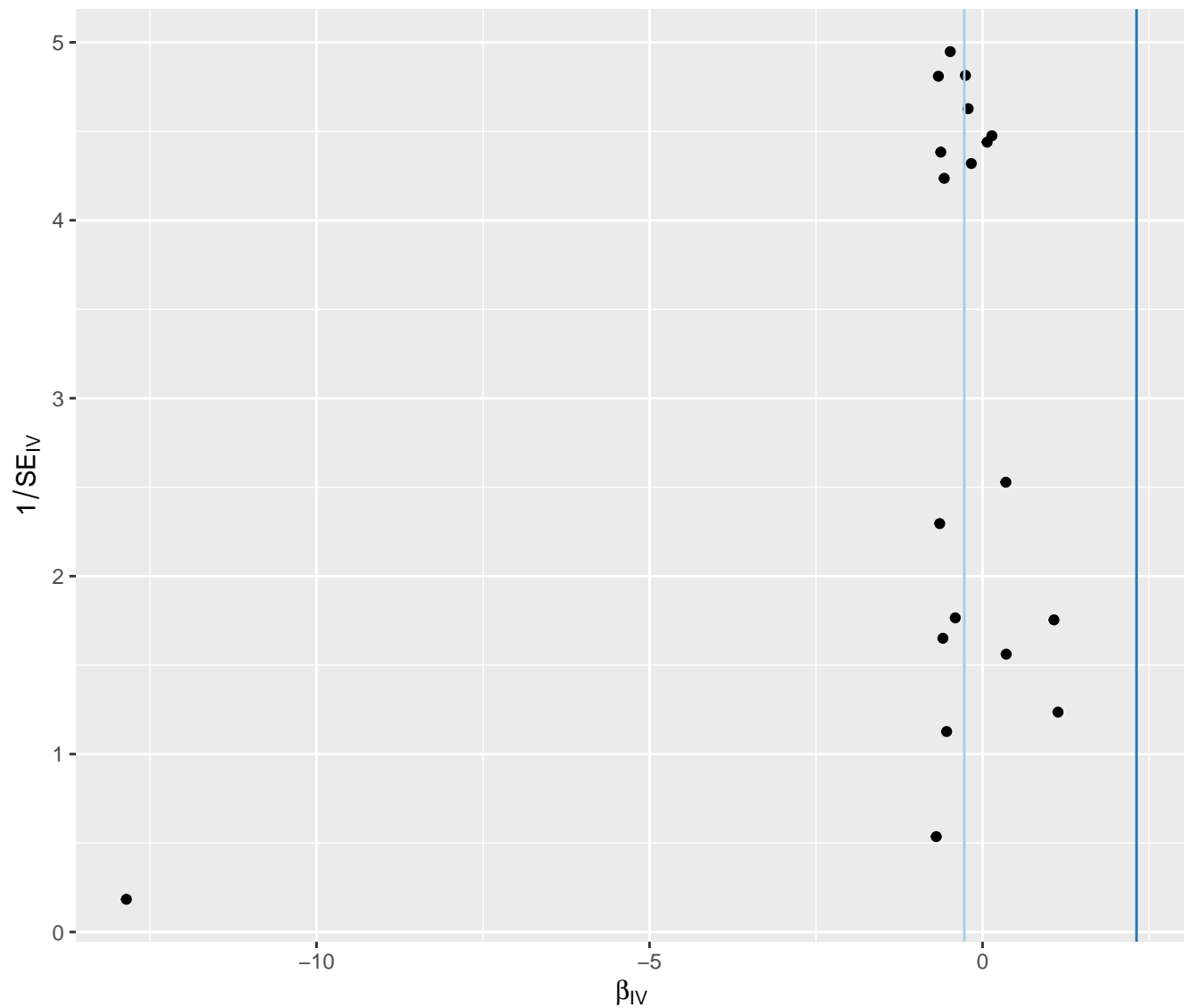

Supplement: Supplementary file 3 [file Supplementaryfile3.zip › Supplementary files 3 funnel plot/tongue-pheno.368.bbj-a-76.pdf]

# MR Method

- Inverse variance weighted
- MR Egger

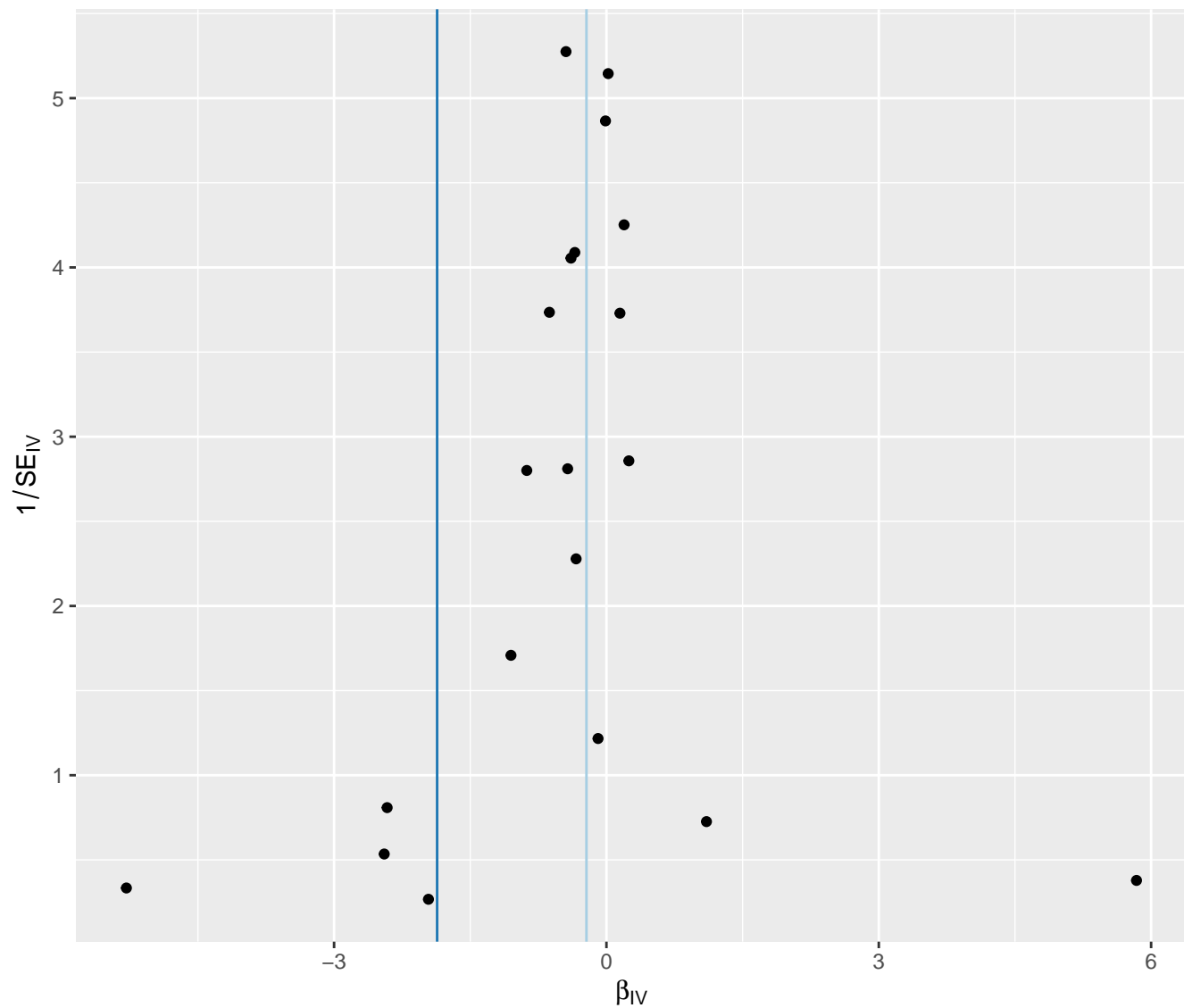

Supplement: Supplementary file 3 [file Supplementaryfile3.zip › Supplementary files 3 funnel plot/tongue-pheno.768.bbj-a-76.pdf]
