# Supplementary material for: Revealing the association between East Asian oral microbiome and colorectal cancer through Mendelian randomization and multi-omics analysis
Source: Front Cell Infect Microbiol. 2024 Sep 17;14:1452392. doi: 10.3389/fcimb.2024.1452392 (PMC11443854; doi:10.3389/fcimb.2024.1452392)
Supplement: Supplementary file 2 [file Supplementaryfile2.zip › Supplementary files 2 leave-one-out plot/tongue-pheno.768.bbj-a-76.pdf]

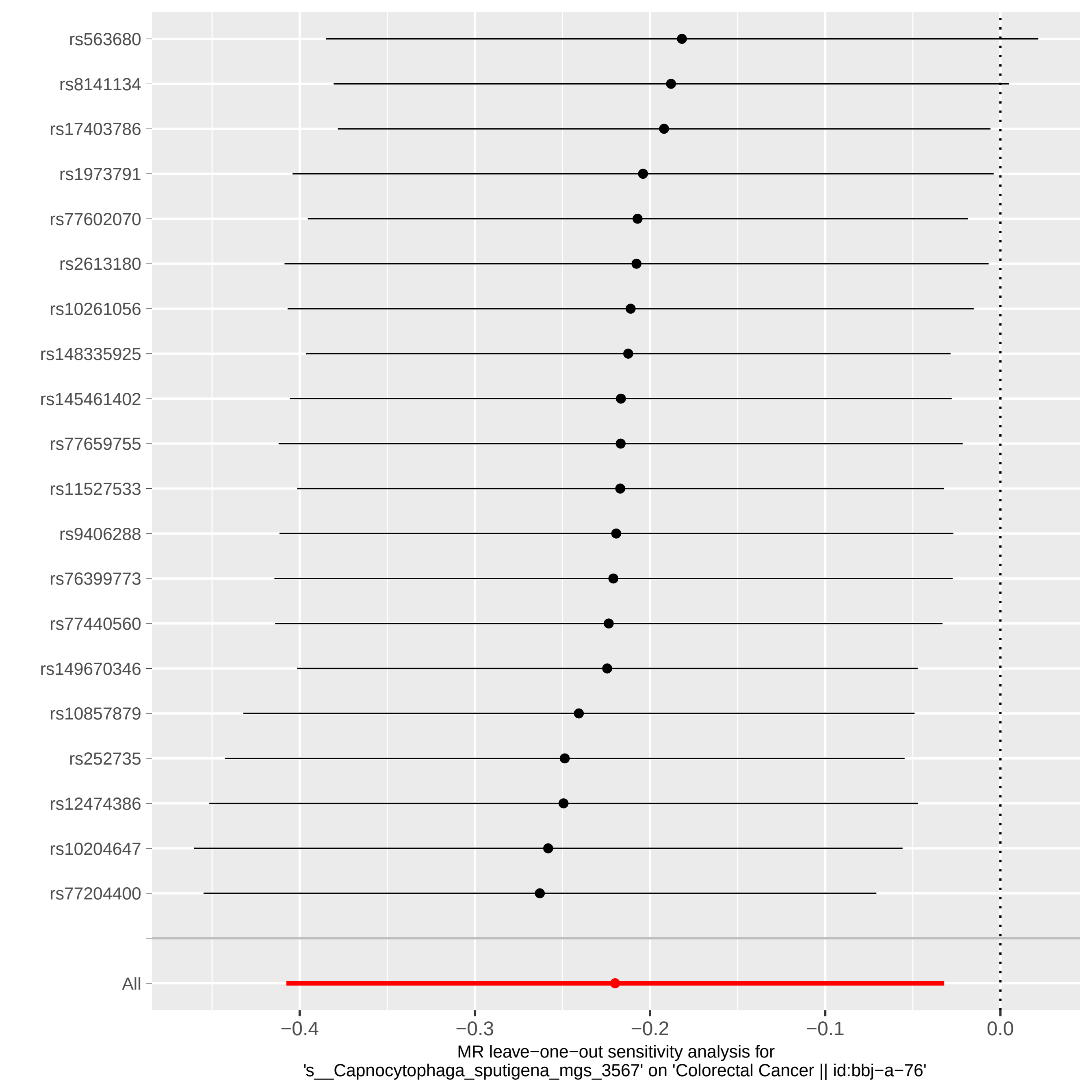

rs563680
rs8141134
rs17403786
rs1973791
rs77602070
rs2613180
rs10261056
rs148335925
rs145461402
rs77659755
rs11527533
rs9406288
rs76399773
rs77440560
rs149670346
rs10857879
rs252735
rs12474386
rs10204647
rs77204400
All
−0.4
−0.3
−0.2
−0.1
0.0
MR leave−one−out sensitivity analysis for
's__Capnocytophaga_sputigena_mgs_3567' on 'Colorectal Cancer || id:bbj−a−76'
